# Bis(oxiranes) Containing Cyclooctane Core: Synthesis and Reactivity towards NaN_3_

**DOI:** 10.3390/molecules27206889

**Published:** 2022-10-14

**Authors:** Kseniya N. Sedenkova, Olga V. Ryzhikova, Svetlana A. Stepanova, Alexei D. Averin, Sergei V. Kositov, Yuri K. Grishin, Igor P. Gloriozov, Elena B. Averina

**Affiliations:** Department of Chemistry, Lomonosov Moscow State University, Leninskie Gory 1–3, 119991 Moscow, Russia

**Keywords:** oxiranes, cyclooctanes, nucleophilic ring opening, domino reactions, azides, polyols, oxabicyclo[3.3.1]nonanes, polyfunctional compounds

## Abstract

Reactions of oxirane ring opening provide a powerful tool for regio- and stereoselective synthesis of polyfunctional and heterocyclic compounds, widely used in organic chemistry and drug design. Cyclooctane, alongside other medium-sized rings, is of interest as a novel molecular platform for the construction of target-oriented leads. Additionally, cyclooctane derivatives are well known to be prone to transannular reactions, which makes them a promising object in the search for novel approaches to polycyclic structures. In the present work, a series of cyclooctanediones was studied in Corey-Chaykovsky reactions, and novel spirocyclic bis(oxiranes) containing cyclooctane core, namely, 1,5-dioxadispiro[2.0.2.6]dodecane and 1,8-dioxadispiro[2.3.2.3]dodecane, were synthesized. Ring opening of the obtained bis(oxiranes) upon treatment with sodium azide was investigated, and it was found that the reaction path is determined by the reciprocal orientation of oxygen atoms in the oxirane moieties. Diastereomers of the bis(oxiranes) with *cis*-orientation underwent independent ring opening, supplying corresponding diazidodiols, while in the case of stereoisomers with *trans*-orientation, domino-like reactions occurred, including intramolecular nucleophilic attack and the formation of a novel three- or six-membered O-containing ring. Summarily, a straightforward approach to polyfunctional compounds containing cyclooctane or oxabicyclo[3.3.1]nonane cores, employing bis(oxiranes), was elaborated.

## 1. Introduction

**Figure 1:**
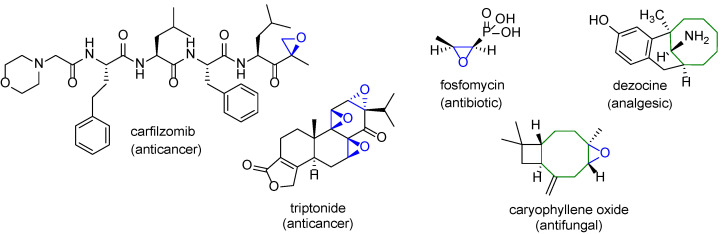
Examples of marketed drugs and bioactive compounds containing oxirane or cyclooctane rings.

Transformations of strained electron-deficient oxirane rings represent a powerful tool in drug design and organic synthesis. Oxirane rings occur in a number of medicinal drugs and bioactive natural compounds (Figure 1) and are widely used for the construction of novel drug candidates, particularly as an alkylating agent [1,2,3,4,5]. Synthetic approaches towards such drugs as atazanavir (HYV protease inhibitor), linezolid (antibiotic), diltiazem (antihypertensive drug), and a number of others include transformations of oxirane moiety [6]. Reactions of oxirane ring opening are widely used as a regio- and stereoselective approach to polyfunctional and heterocyclic compounds, and novel reactions and synthetic procedures employing oxiranes are still being developed [7,8,9,10,11,12,13,14]. The presence of two or more oxirane moieties in a molecule creates the opportunity for a straightforward synthesis of polyfunctional compounds, and for the use of such a molecule as a linker in the construction of multivalent ligands.

Cyclooctane, alongside other medium rings, is characterized by an optimal balance of conformational rigidity and flexibility and is of interest as a novel molecular platform for the design of target-oriented leads [15,16,17,18]. On the other hand, the synthetic application of ring-closure reactions to medium rings is often limited because of the entropy factor disfavoring ring closure. Therefore, the search for simple preparative approaches to the functionalization of already existing cyclooctane moiety poses an important problem [19,20]. Additionally, a number of transannular reactions can proceed due to cyclooctane conformational transitions, including those starting from oxirane ring-opening processes [21,22].

**Scheme 1:**
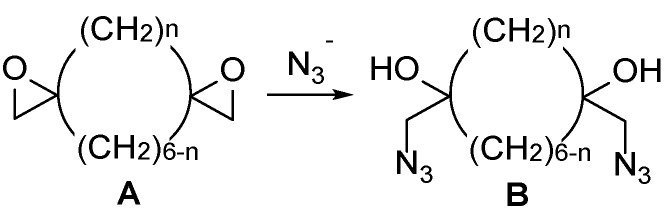
Ring opening of bis(oxiranes) **A**, containing cyclooctane core.

This work is therefore aimed at the synthesis of novel bis(oxiranes) **A**, containing cyclooctane core, the investigation of the reactions with azide anion, and the preparation of polyfunctional compounds **B** starting from the bis(oxiranes) (Scheme 1).

## 2. Results and discussion

### 2.1. Synthesis of Bis(oxiranes) via Corey-Chaykovsky Reaction of Cyclooctanediones

**Scheme 2:**
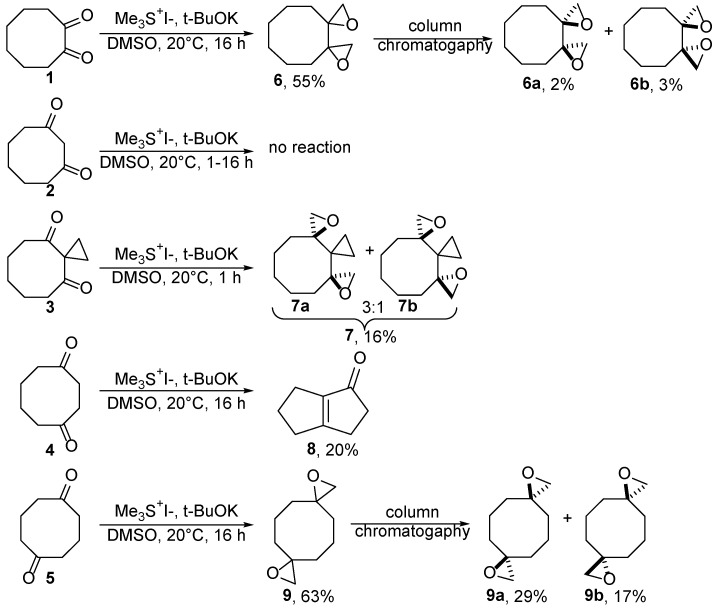
Investigation of diketones **1**–**5** in Corey-Chaykovsky reaction.

In order to obtain previously unknown bis(oxiranes), cyclooctanediones **1**–**5** were investigated in a Corey-Chaykovsky reaction using sulfur ylide derived from trimethylsulfonium iodide and potassium *tert*-butoxide (Scheme 2).

Bis(oxirane) **6** was obtained from cyclooctane-1,2-dione (**1**) as a mixture of diastereomers in good yield (Scheme 2). In order to study the difference in the reactivity of stereomers of compound **6**, individual diastereomer **6a** (*meso* form) and **6b** (as racemate) were isolated via preparative column chromatography.

The interaction of cyclooctane-1,3-dione (**2**) and sulfur ylide produced no bis(oxirane), which is probably due to the tendency of 1,3-diketone to produce enolate under basic conditions. As such, 1,3-diketone **3**, containing a spirocyclopropane moiety between the carbonyl groups, was employed in a Corey-Chaykovsky reaction, yielding bis(oxirane) **7** as a mixture of *meso* form **7a** and racemate **7b** in ratio 3:1 (Scheme 2). The reaction proceeded in low yield and a decrease in the reaction time down to 1 h was required in order to prevent the decomposition of the products. The lability of compounds **7a**,**b** prevented their isolation via column chromatography, and full description of NMR spectra could be accomplished only for the isomer **7a** prevailing in the reaction mixture.

Cyclooctane-1,4-dione (**4**), in the presence of potassium *tert*-butoxide and sulfur ylide, which may also act as a base, underwent a well-known [23,24] intramolecular condensation, producing bicyclic ketone **8** instead of the corresponding bis(oxirane) (Scheme 2).

Finally, cyclooctane-1,5-dione (**5)** was found to smoothly react with sulfur ylide, producing bis(oxirane) **9** in good yield (Scheme 2). Individual diastereomers **9a** and **9b** were isolated via column chromatography.

The relative configuration of compounds **6a,b** was determined using a calculation of ^13^C NMR chemical shifts. The assignments of configuration for **7a,b** and **9a,b** were made on the basis of NMR spectra, taking into account differences in the symmetry of molecules (see Supplementary Materials for details).

### 2.2. Ring Opening of Bis(oxiranes) with Sodium Azide

In order to compare the reactivity of bis(oxiranes) with different reciprocal positions of three-membered rings, compounds **6a,b** and **9a,b** were investigated by the treatment with a well-known nucleophile: sodium azide. It should be mentioned that organic azides are of the utmost interest as versatile intermediates in organic synthesis and can be found in a variety of pharmaceuticals and biologically active compounds, such as Zidovudine, Azidamfenicol, Azidocillin, and others [25,26,27,28].

**Scheme 3:**
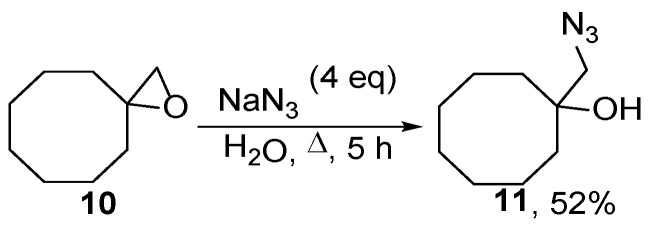
Ring opening of oxirane **10** with sodium azide.

The conditions of the ring opening of oxiranes containing spriroannelated cyclooctane moiety were probed for model oxaspirodecane **10**. It was found that the reaction of compound **10,** with a four-fold excess of sodium azide in water under reflux, produces azidoalcohol **11** as a sole product (Scheme 3).

**Scheme 4:**
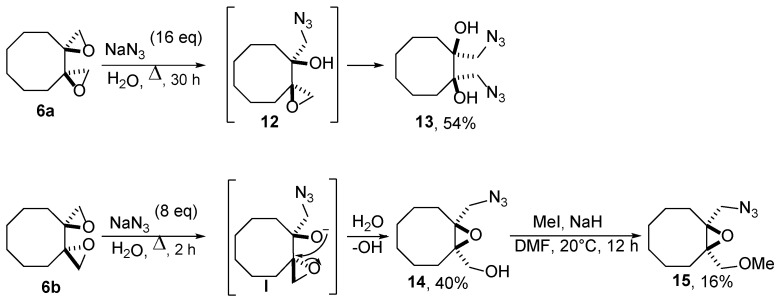
Ring opening of bis(oxiranes) **6a,b** with sodium azide.

Under the same conditions, compound **6a** interacted with sodium azide, producing predominantly product **12**, resulting from the opening of one of two oxirane rings, which was obtained as a poorly separable mixture with diazidodiol **13** (see Section 3.3 and Supplementary Materials). To obtain diazidodiol **13** as the sole product, an additional optimization of reaction conditions was conducted (see Supplementary Materials). Varying solvents, reaction times and reagents ratios demonstrated that for the full conversion of compound **6a** into diazidodiol **13,** 16-fold excess of nucleophilic agent and reflux in water for 30 h are required (Scheme 4).

Bis(oxirane) **6b**, on the contrary, smoothly reacted with sodium azide, producing oxirane **14** as a sole product and no products of independent ring opening similar to compounds **12** or **13** were observed (Scheme 4). Thus, in the reaction of **6b** with nucleophile, a fairly rare reaction pathway, described for bis(oxiranes) containing neighboring oxirane moieties [29,30], was observed. In this case, anion **I**, formed after the opening of first oxirane ring, underwent intramolecular nucleophilic attack of the oxygen atom to form a new oxirane moiety. Such reaction is highly improbable for diastereomer **6a** because in the case of oxirane **12** (or corresponding anion), a nucleophilic attack of oxygen must proceed “from the front”.

The presence of a hydroxyl group in compound **14** was additionally confirmed via the methylation reaction. Treatment of oxirane **14** with an excess of methyl iodide in the presence of NaH produced methyl ether **15** as the sole product (Scheme 4).

**Scheme 5:**
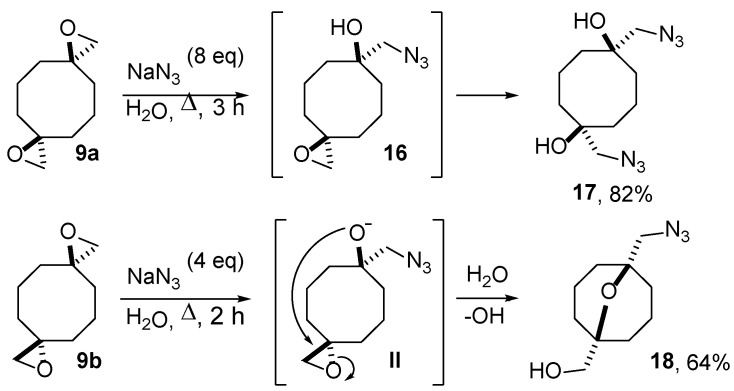
Ring opening of bis(oxiranes) **9a,b** with sodium azide.

The interaction of two diastereomers of bis(oxirane) **9** with sodium azide also proceeded via two different pathways. It was found that the ring opening of both oxirane moieties in compound **9a** occurs upon treatment with eight-fold excess of nucleophile under reflux for 3 h, producing diazidodiol **17** as the only product in high yield (Scheme 5). When the reaction time was shorter, a mixture of compounds **16** and **17** was obtained, with diazidodiol **17** prevailing (see Section 3.3 and Supplementary Materials).

The reaction of bis(oxirane) **9b**, containing *trans*-oriented oxygen atoms, with sodium azide required shorter time (2 h) and lower excess of nucleophile (4 eq), and again proceeded in an unexpected way, producing oxabicyclononane **18** in good yield (Scheme 5). The formation of oxabicyclononane **18** presumably resulted from the ring opening of an oxirane moiety, producing anion **II**, and subsequent intramolecular nucleophilic attack of oxygen on the second oxirane ring. It should be noted that no examples of the formation of tetrahydropyran moiety via domino ring opening of bis(oxiranes) has been found in earlier research. This reaction opens the way to hardly accessible oxabicyclononane derivatives, which, like bicyclononanes [31,32] and azabicyclononanes [33], represent promising 3D scaffolds for drug design.

Thus, it was demonstrated that diastereomers of bis(oxiranes) with cyclooctane cores possess different reactivity towards azide anion. Compounds **6a** and **9a**, containing *cis*-oriented oxygen atoms, are less reactive and undergo independent ring opening of oxirane moieties, whereas compounds **6b** and **9b**, containing oxygen atoms in *trans*-position, undergo relatively fast domino-type ring opening of oxirane rings and generate products of intramolecular nucleophilic attack **14,18**.

## 3. Materials and Methods

### 3.1. General Remarks

^1^H and ^13^C NMR spectra were recorded on a 400 MHz spectrometer Agilent 400-MR (400.0 and 100.6 MHz for ^1^H and ^13^C, respectively) at r.t. in CDCl_3_, while chemical shifts δ were measured with reference to the solvent (CDCl_3_, δ_H_ = 7.26 ppm, δ_C_ = 77.16 ppm). When necessary, assignments of signals in NMR spectra were made using 2D techniques (see Supplementary Materials). Accurate mass measurements (HRMS) were obtained on Bruker micrOTOF II with electrospray ionization (ESI). Analytical thin-layer chromatography was carried out with silica gel plates supported on aluminum (Macherey-Nagel, ALUGRAM^®^ Xtra SIL G/UV_254_), with inspection using a UV lamp (254 nm). Column chromatography was performed on silica gel (Macherey-Nagel, Silica 60, 0.015–0.04 mm). Cyclooctanediones **1** [34], **2** [35], **3** [36], **4** and **5** [37] (A mixture of 1,4-diketone (**4**) and 1,5-diketone (**5**) in ratio ~1:1 was obtained when the procedure described in ref. [37] was reproduced, though in the article 1,5-diketone (**5**) is described to be the only product; diketones **4** and **5** were separated via column chromatography), trimethylsulfonium iodide [38], and oxirane **10** [39] were obtained via the described methods. All other starting materials were commercially available. All reagents except commercial products of satisfactory quality were purified according to the literature procedures prior to use.

### 3.2. Synthesis of Bis(oxiranes) (General Method)

To the solution of trimethylsulfonium iodide (6.6 g, 32.4 mmol) in 60 mL of dry DMSO, the solution of corresponding cyclooctanedione (1.4 g, 10 mmol) in 5 mL of dry DMSO was added dropwise at stirring under argon. Then the solution of potassium *tert*-butoxide (3.36 g, 30 mmol) in 40 mL of dry DMSO was added dropwise. The reaction mixture was stirred for 16 h at r.t., then it was poured into icy water (60 mL) and extracted with pentane (3 × 20 mL). Combined organic layers were quickly dried over MgSO_4_; the solvent was evaporated under reduced pressure. The products were isolated via preparative column chromatography (SiO_2_).

(3*R*,4*S*)-1,5-Dioxadispiro[2.0.2.6]dodecane **(6a)**.

Yield 2% (34 mg), yellowish liquid, R*f* = 0.38 (CH_2_Cl_2_:light petrol 3:1).

^1^H NMR (400 MHz, CDCl_3_, 25 °C): *δ=* 1.51–1.85 (m, 10H, 6CH_2_), 1.85–1.96 (m, 2H, 2CH_2_), 2.63 (d, ^2^*J* = 5.3, 2H, 2CH_2_O), 2.85 (dd, ^2^*J* = 5.3, ^3^*J* = 0.9, 2H, 2CH_2_O); ^13^C NMR (101 MHz, CDCl_3_, 25 °C): *δ* *=* 22.8 (2CH_2_^β^), 25.9 (2CH_2_^γ^), 33.8 (2CH_2_^α^), 53.1 (2CH_2_O), 60.3 (2C_spiro_).

HRMS (ESI^+^, 70 eV, *m*/*z*): calculated for C_10_H_16_O_2_ [M+H]^+^: 169.1223; found: 169.1229.

(3*R*,4*R*)/(3*S*,4*S*)-1,5-Dioxadispiro[2.0.2.6]dodecane **(6b)**.

Yield 3% (50 mg), yellowish liquid, R*f* = 0.18 (CH_2_Cl_2_: light petrol 3:1).

^1^H NMR (400 MHz, CDCl_3_, 25 °C): *δ=* 1.43–1.67 (m, 4H, 4CH_2_), 1.69–1.84 (m, 6H, 6CH_2_), 1.90–2.01 (m, 2H, 2CH_2_), 2.59 (d, ^2^*J* = 5.7, 2H, 2CH_2_O), 2.87 (d, ^2^*J* = 5.7, 2H, 2CH_2_O); ^13^C NMR (101 MHz, CDCl_3_, 25 °C): *δ* *=* 25.1 (2CH_2_^β^), 25.4 (2CH_2_^γ^), 32.3 (2CH_2_^α^), 52.3 (2CH_2_O), 58.7 (2 C_spiro_).

HRMS (ESI^+^, 70 eV, *m*/*z*): calculated for C_10_H_16_O_2_ [M+H]^+^: 169.1223; found: 169.1229.

1,8-Dioxadispiro[2.0.2.0.2.5]tetradecane **(7)**.

Yield 16% (310 mg), obtained as a mixture of diastereomers **7a:7b** 3:1, colorless liquid, R*f* = 0.27 (CH_2_Cl_2_)

**7a:**^1^H NMR (400 MHz, CDCl_3_, 25 °C): *δ=* 0.39–0.45 (m, 2H, CH_2_, cy-Pr), 0.65–0.71 (m, 2H, CH_2_, cy-Pr), 1.38–1.46 (m, 2H, C^10^H_2_, C^14^H_2_), 1.52–1.71 (m, 2H, C^11^H_2_, C^13^H_2_ + 2H, C^12^H_2_), 1.89–1.96 (m, 2H, C^11^H_2_, C^13^H_2_), 1.99–2.07 (m, 2H, C^10^H_2_, C^14^H_2_), 2.61 (s, 4H, 2CH_2_O); ^13^C NMR (101 MHz, CDCl_3_, 25 °C): *δ* *=* 6.5 (CH_2_, cy-Pr), 8.8 (CH_2_, cy-Pr), 22.4 (C^11^H_2_, C^13^H_2_), 26.6 (C^4^), 26.7 (C^12^H_2_), 34.1 (C^10^H_2_, C^14^H_2_), 54.2 (2CH_2_O), 59.0 (C^3^,C^7^).

**7b:**^1^H NMR (400 MHz, CDCl_3_, 25 °C): *δ=* 0.40–0.47 (m, 4H, CH_2_, cy-Pr), 1.47–1.56 (m, 2H, C^10^H_2_, C^14^H_2_), 1.60–1.66 (m, 2H, C^12^H_2_), 1.71–1.80 (m, 4H, C^11^H_2_, C^13^H_2_), 2.0–2.06 (m, 2H, C^10^H_2_, C^14^H_2_); ^13^C NMR (101 MHz, CDCl_3_, 25 °C): *δ* *=* 7.3 (2C, CH_2_, cy-Pr), 24.5 (C^11^H_2_, C^13^H_2_), 25.5 (C^12^H_2_), 25.9 (C^4^), 34.7 (C^10^H_2_, C^14^H_2_), 54.6 (2CH_2_O), 58.99 (C^3^,C^7^).

HRMS (ESI^+^, 70 eV, *m*/*z*): calculated for C_12_H_18_O_2_ [M+H]^+^: 195.1380; found: 195.1384.

3,4,5,6-Tetrahydropentalen-1(2*H*)-one (**8**) [23].

Yield 20% (244 mg), colorless oil, R*f* = 0.19 (light petrol:EtOAc 10:3).

^1^H NMR (400 MHz, CDCl_3_, 25 °C): *δ=* 2.25–2.38 (m, 4H, 2CH_2_), 2.43–2.56 (m, 4H, 2CH_2_), 2.66–2.76 (m, 2H, CH_2_); ^13^C NMR (101 MHz, CDCl_3_, 25 °C): *δ* *=* 24.5 (CH_2_), 25.7 (CH_2_), 27.9 (CH_2_), 32.1 (CH_2_), 41.2 (CH_2_), 149.0 (C), 187.4 (C), 204.0 (C=O).

(3*s*,7*s*)-1,8-Dioxadispiro[2.3.2.3]dodecane **(9a)**.

Yield 29% (487 mg), yellowish liquid, R*f* = 0.36 (light petrol:EtOAc 3:1).

^1^H NMR (400 MHz, CDCl_3_, 25 °C): *δ=* 1.47 (ddd, 4H, ^2^*J* = 14.4, ^3^*J* = 9.0, ^3^*J* = 3.6, 4CH_2_^α^), 1.55–1.66 (m, 2H, 2CH_2_^β^), 1.79–1.92 (m, 2H, 2CH_2_^β^), 2.01 (ddd, 4H, ^2^*J* = 14.4, ^3^*J* = 8.8, ^3^*J* = 3.4, 4CH_2_^α^), 2.63 (s, 4H, 2CH_2_O); ^13^C NMR (101 MHz, CDCl_3_, 25 °C): *δ* *=* 22.4 (2CH_2_^β^), 34.8 (4CH_2_^α^), 54.9 (2CH_2_O), 59.6 (2 C_spiro_).

HRMS (ESI^+^, 70 eV, *m*/*z*): calculated for C_10_H_16_O_2_ [M+Na]^+^: 191.1043; found: 191.1042.

(3*r*,7*r*)-1,8-Dioxadispiro[2.3.2.3]dodecane **(9b)**.

Yield 17% (286 mg), yellowish liquid, R*f* = 0.49 (light petrol:EtOAc 3:1).

^1^H NMR (400 MHz, CDCl_3_, 25 °C): *δ=* 1.62–1.85 (m, 12H, 6CH_2_), 2.67 (s, 4H, 2CH_2_O); ^13^C NMR (101 MHz, CDCl_3_, 25 °C): *δ* *=* 21.7 (2CH_2_^β^), 34.6 (4CH_2_^α^), 55.6 (2CH_2_O), 59.1 (2 C_spiro_).

HRMS (ESI^+^, 70 eV, *m*/*z*): calculated for C_10_H_16_O_2_ [M+H]^+^: 169.1223; found: 169.1228.

### 3.3. Ring Opening of Oxiranes upon Treatment with Sodium Azide (General Method)

To the solution of sodium azide (2–32 mmol) in water (2 mL), the corresponding oxirane (1 mmol) was added. The reaction mixture was stirred under reflux for 2–30 h, cooled down to r.t. and extracted with ethyl acetate (3 × 3 mL). The organic layers were combined; the solvent was evaporated under reduced pressure. The products were isolated via preparative column chromatography (SiO_2_).

1-(Azidomethyl)cyclooctanol **(11)**.

Obtained from oxirane **10** and sodium azide (0.26 g, 4 mmol). Reaction time 5 h. Yield 52% (95 mg), colorless oil, R*f* = 0.32 (light petrol:EtOAc 10:1).

^1^H NMR (400 MHz, CDCl_3_, 25 °C): *δ=* 1.35–1.72 (m, 12H, 7CH_2_), 1.72–1.83 (m, 2H, 2CH_2_), 3.26 (s, 2H, CH_2_N_3_); ^13^C NMR (101 MHz, CDCl_3_, 25 °C): *δ* *=* 22.1 (2CH_2_), 24.9 (CH_2_), 28.2 (2CH_2_), 33.9 (2CH_2_), 60.8 (CH_2_N_3_), 75.3 (C).

HRMS (ESI^+^, 70 eV, *m*/*z*): calculated for C_9_H_17_N_3_O [M+Na]^+^: 206.1264; found: 206.1253.

(3*R*,4*S*)/(3*S*,4*R*)-4-(Azidomethyl)-1-oxaspiro[2.7]decan-4-ol (**12**).

Obtained from bis(oxirane) **6a** and sodium azide (0.52 g, 8 mmol). Reaction time 3 h. Yield 16% (33 mg), colorless oil, R*f* = 0.55 (light petrol:EtOAc 6:1).

^1^H NMR (400 MHz, CDCl_3_, 25 °C): 1.34–1.95 (m, 12H, 6CH_2_), 2.47 (s, 1H, OH), 2.61 (d, 1H, ^2^*J* = 4.9, CH_2_O), 3.08 (d, 1H, ^2^*J* = 4.9, CH_2_O), 3.25 (s, 2H, CH_2_N_3_); ^13^C NMR (101 MHz, CDCl_3_, 25 °C): 21.3 (CH_2_), 24.9 (CH_2_), 25.0 (CH_2_), 25.7 (CH_2_), 30.3 (CH_2_), 31.6 (CH_2_), 53.5 (CH_2_O, ^1^*J*_CH_ = 173), 58.1 (CH_2_N_3_, ^1^*J*_CH_ = 141), 61.0 (C), 73.9 (C).

HRMS (ESI^+^, 70 eV, *m*/*z*): calculated for C_10_H_1__7_N_3_O_2_ [M+Na]^+^: 234.1213; found: 234.1220.

(*1R,2S*)/(*1S,2R*)-1,2-Bis(azidomethyl)cyclooctane-1,2-diol **(13)**.

Obtained from bis(oxirane) **6a** and sodium azide (1.04 g, 16 mmol). Reaction time 30 h. Yield 54% (136 mg), yellow oil, R*f* = 0.76 (light petrol:EtOAc 4:1).

^1^H NMR (400 MHz, CDCl_3_, 25 °C): *δ=* 1.41–1.54 (m, 4H, 2CH_2_), 1.55–1.70 (m, 4H, 2CH_2_), 1.74–1.90 (m, 2H, 2CH_2_), 1.94–2.04 (m, 2H, 2CH_2_), 2.87 (s, 2H, 2 OH), 3.29 (d, 2H, ^2^*J* = 12.4, 2CH_2_N_3_), 3.51 (d, 2H, ^2^*J* = 12.4, 2CH_2_N_3_); ^13^C NMR (101 MHz, CDCl_3_, 25 °C): *δ* *=* 21.6 (2CH_2_), 28.1 (2CH_2_), 32.6 (2CH_2_), 57.3 (2CH_2_N_3_), 77.3 (2 C).

HRMS (ESI^+^, 70 eV, *m*/*z*): calculated for C_10_H_18_N_6_O_2_ [M+Na]^+^: 277.1383; found: 277.1387.

((1*R*,8*S*)/(1*S*,8*R*)-8-(Azidomethyl)-9-oxabicyclo[6.1.0]nonan-1-yl)methanol **(14)**.

Obtained from bis(oxirane) **6b** and sodium azide (0.52 g, 8 mmol). Reaction time 2 h. Yield 40% (84 mg), yellow oil, R*f* = 0.10 (light petrol:EtOAc 4:1).

^1^H NMR (400 MHz, CDCl_3_, 25 °C): *δ=* 1.40–1.70 (m, 10H, 6CH_2_), 1.90 (br.m, 1H, OH), 2.24–2.37 (m, 2H, 2CH_2_), 3.52 (d, 1H, ^2^*J* = 13.5, CH_2_N_3_), 3.62 (d, 1H, ^2^*J* = 13.5, CH_2_N_3_), 3.72 (dd, 1H, ^2^*J* = 12.2, ^3^*J* = 4.6, CH_2_O), 3.86 (dd, 1H, ^2^*J* = 12.2, ^3^*J* = 5.4, CH_2_O); ^13^C NMR (101 MHz, CDCl_3_, 25 °C): *δ* *=* 25.1 (CH_2_), 25.4 (CH_2_), 26.49 (CH_2_), 26.51 (CH_2_), 29.57 (CH_2_), 29.63 (CH_2_), 52.3 (CH_2_N_3_), 62.5 (CH_2_O), 66.0 (C), 66.3 (C).

HRMS (ESI^+^, 70 eV, *m*/*z*): calculated for C_10_H_19_N_3_O_3_ [M+Na]^+^ 252.1319; found: 252.1315.

(3s,7*s*)-7-(Azidomethyl)-1-oxaspiro[2.7]decan-7-ol **(16)**.

Obtained from bis(oxirane) **9a** and sodium azide (0.26 g, 4 mmol). Reaction time 2 h. Yield 14% (30 mg), yellowish liquid, R*f* = 0.27 (light petrol:EtOAc 3:1).

^1^H NMR (400 MHz, CDCl_3_, 25 °C): *δ=* 1.41–1.53 (m, 2H, 2CH_2_), 1.60–1.74 (m, 4H, 4CH_2_), 1.74–1.91 (m, 6H, 6CH_2_), 2.62 (s, 2H, CH_2_O), 3.26 (s, 2H, CH_2_N_3_); ^13^C NMR (101 MHz, CDCl_3_, 25 °C): *δ* *=* 19.2 (2CH_2_), 34.1 (2CH_2_), 35.7 (2CH_2_), 55.4 (CH_2_O), 58.9 (C_spiro_), 60.9 (CH_2_N_3_), 75.1 (C).

HRMS (ESI^+^, 70 eV, *m*/*z*): calculated for C_10_H_17_N_3_O_2_ [M+Na]^+^: 234.1213; found: 234.1215.

(1*s*,5*s*)-1,5-Bis(azidomethyl)cyclooctane-1,5-diol **(17)**.

Obtained from bis(oxirane) **9a** and sodium azide (0.52 g, 8 mmol). Reaction time 6 h. Yield 82% (208 mg), yellowish liquid, R*f* = 0.17 (light petrol:EtOAc 3:1).

^1^H NMR (400 MHz, CDCl_3_, 25 °C): *δ=* 1.45–1.62 (m, 6H, 6CH_2_), 1.81–1.96 (m, 6H, 6CH_2_), 2.28 (br.s, 2H, 2 OH), 3.21 (s, 4H, 2CH_2_N_3_); ^13^C NMR (101 MHz, CDCl_3_, 25 °C): *δ* *=* 17.9 (2CH_2_), 36.4 (4CH_2_), 62.6 (2CH_2_N_3_), 74.1 (2 C).

HRMS (ESI^+^, 70 eV, *m*/*z*): calculated for C_10_H_18_N_6_O_2_ [M+H]^+^: 255.1564; found: 255.1570.

[5-(Azidomethyl)-9-oxabicyclo[3.3.1]non-1-yl]methanol **(18)**.

Obtained from bis(oxirane) **9b** and sodium azide (0.26 g, 4 mmol). Reaction time 2 h. Yield 64% (136 mg), yellowish liquid, R*f* =0.37 (CH_2_Cl_2_).

^1^H NMR (400 MHz, CDCl_3_, 25 °C): *δ=* 1.31–1.48 (m, 4H, C^2^H_2_, C^4^H_2_, C^6^H_2_, C^8^H_2_), 1.59–1.76 (m, 6H, C^2^H_2,_ C^3^H_2_, C^4^H_2_, C^6^H_2_, C^7^H_2_, C^8^H_2,_), 1.93–2.13 (m, 3H, C^3^H_2_, C^7^H_2_, OH), 3.03 (s, 2H, CH_2_N_3_), 3.34 (d, 2H, ^3^*J* = 6.4, CH_2_OH); ^13^C NMR (101 MHz, CDCl_3_, 25 °C): *δ* *=* 18.4 (C^3^H_2_, C^7^H_2_), 29.5 (C^2^H_2_, C^8^H_2_), 31.0 (C^4^H_2_, C^6^H_2_), 61.4 (CH_2_N_3_), 71.4 (CH_2_OH), 72.9 (C^1^), 73.9 (C^5^).

HRMS (ESI^+^, 70 eV, *m*/*z*): calculated for C_10_H_17_N_3_O_2_ [M+H]^+^: 212.1394; found: 212.1388.

### 3.4. Synthesis of (1R,8S)/(1S,8R)-1-(Azidomethyl)-8-(methoxymethyl)-9-oxabicyclo[6.1.0]nonane (**15**)

To the solution of alcohol **14** (0.21 g, 1 mmol) and methyl iodide (0.72 g, 0.33 mL, 5.7 mmol) in dry DMF (12 mL), NaH (60% suspension in oil; 0.14 g, 3.6 mmol) was added. The reaction mixture was stirred for 12 h at r.t., quenched with saturated aqueous NH_4_Cl (10 mL), and extracted with EtOAc (3 × 5 mL). Combined organic layers were dried over MgSO_4_; the solvent was evaporated under reduced pressure. The product was isolated via preparative column chromatography (SiO_2_).

Yield 16% (36 mg), brown oil, R*f* = 0.38 (CH_2_Cl_2_:light petrol 1:1).

^1^H NMR (400 MHz, CDCl_3_, 25 °C): *δ=* 1.36–1.66 (m, 10H, 6CH_2_), 2.23–2.35 (m, 2H, 2CH_2_), 3.36 (s, 3H, CH_3_), 3.431 (d, 1H, *^2^J* = 11.1, CH_2_O), 3.438 (d, 1H, ^2^*J* = 13.4, CH_2_N_3_), 3.53 (d, 1H, *^2^J* = 13.4, CH_2_N_3_), 3.73 (d, 1H, *^2^J* = 11.1, CH_2_O); ^13^C NMR (101 MHz, CDCl_3_, 25 °C): *δ* *=* 25.2 (CH_2_), 25.5 (CH_2_), 26.2 (CH_2_), 26.7 (CH_2_), 29.2 (CH_2_), 29.9 (CH_2_), 52.8 (CH_2_N_3_), 59.3 (CH_3_O), 64.6 (C), 65.1 (C), 74.2 (CH_2_O).

HRMS (ESI^+^, 70 eV, *m*/*z*): calculated for C_11_H_19_N_3_O_2_ [M+Na]^+^: 248.1369; found: 248.1369.

## 4. Conclusions

To summarize, novel bis(oxiranes), containing cyclooctane core, were synthesized and investigated upon treatment with sodium azide. Configuration of bis(oxiranes) was found to drastically influence on their reactivity towards azide anion. A novel pathway of the reaction of 1,3-bis(oxiranes) with a nucleophile, producing oxabicyclononane moiety, was found. Preparative approaches towards a series of novel cyclooctane and oxabicyclononane derivatives, containing azido and hydroxy groups, starting from spirocyclic oxiranes and employing simple and convenient methods, were realized.

## Data Availability

Not applicable.

## Supplementary Materials

The following supporting information can be downloaded at: https://www.mdpi.com/article/10.3390/molecules27206889/s1, assignment of relative configuration of diastereomers of bis(oxiranes); optimization of conditions of the ring opening of **6a** and **9a**; copies of NMR spectra of the novel compounds [40,41,42,43,44,45].

## Sample Availability

Samples of all the described compounds are available from the authors.
