# Supplementary material for: Bis(oxiranes) Containing Cyclooctane Core: Synthesis and Reactivity towards NaN3
Source: Molecules. 2022 Oct 14;27(20):6889. doi: 10.3390/molecules27206889 (PMC9607513; doi:10.3390/molecules27206889)
Supplement: Supplementary file 1 [file molecules-27-06889-s001.zip › molecules-1968970-supplementary.pdf]

Bis(oxiranes) containing cyclooctane core: synthesis and reactivity towards NaN_3

Kseniya N. Sedenkova *, Olga V. Ryzhikova, Svetlana A. Stepanova, Alexei D. Averin *, Sergei V. Kositov, Yuri K. Grishin, Igor P. Gloriozov and Elena B. Averina

Supplementary information

1. **Assignment of relative configuration of diastereomers of bis(oxiranes) 2**
2. **Optimization of reactions conditions 10**
3. **Copies of NMR spectra 11**

1. Assignment of relative configuration of diastereomers of bis(oxiranes)

1.1. Bis(oxiranes) 6a and 6b

The geometries of molecules were fully optimized by means of density functional theory (DFT) calculations. The PBE functional [40] and electron basis sets L1 were used, where L1 stands for double set size. The numbers of contracted and primitive functions used in L1 are respectively {2,1}/{6,2} for H, {3,2,1}/{10,7,3} for C, and O [41,42]. Stationary points on the potential energy surface (PES) were identified by analyzing Hessians. The thermodynamic functions (Gibbs energies, G) at 298.15 K were calculated using an approximation of restricted rotator and harmonic oscillator. The ^{13}C NMR spectra were calculated using gaugeincluding atomic orbitals (GIAO) [43,44] in the complete electronic L1 basis. All calculations were performed using the MBC100k cluster at the Joint Supercomputer Center (JSCC) (Moscow, Russia) with the use of the PRIRODA04 program written by Laikov [45].

The relative energies and ^{13}C NMR chemical shifts of the most stable conformations of bis(oxirane) **6** diastereomers were calculated using DFT method (Figure S1, Table S2, S7). The most stable conformations of *meso*-diastereomer **6a** were found to be “crown” (G=0 kcal·mol^{-1}), twisted “boat-boat” (G=0.1 kcal·mol^{-1}) and “chair-boat” (G=1.6 kcal·mol^{-1}). For **6b** the most stable conformations were found to be “boat-boat” (G=0.5 kcal·mol^{-1}) and two “chair-boat” (G=0.8 kcal·mol^{-1} and G=3.0 kcal·mol^{-1}) with different orientations of oxygen atoms.

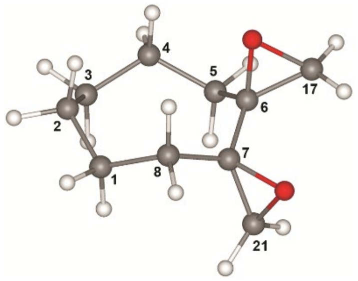

6a-1
“crown”
G=0 kcal·mol^{-1}

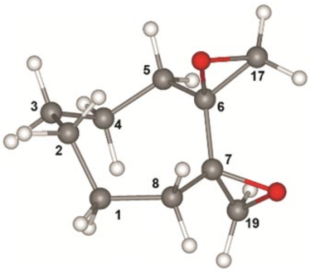

6a-2
twisted “boat-boat”
G=0.1 kcal·mol^{-1}

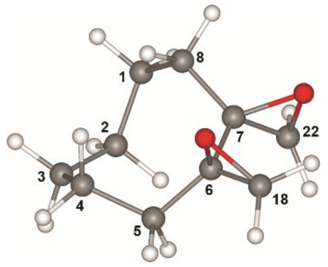

6a-3
“chair-boat”
G=1.6 kcal·mol^{-1}

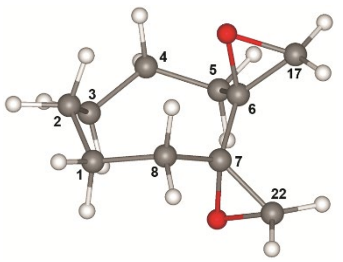

6b-1
“boat-boat”
G=0.5 kcal·mol^{-1}

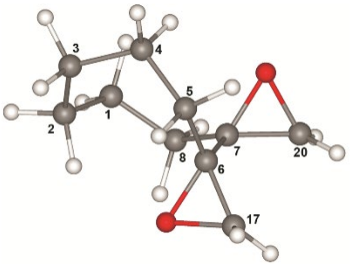

6b-2
“chair-boat”
G=0.8 kcal·mol^{-1}

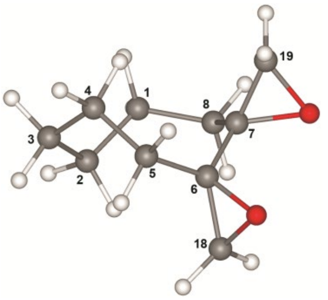

6b-3
“chair-boat”
G=3.0 kcal·mol^{-1}

Figure S1. The most stable conformations of diastereomers of bis(oxirane) **6**

The relative content of conformers was estimated for **6a** and **6b** basing on the relative energies (Table S1).

Table S1. The relative content of conformers of **6a** and **6b**[a]

Conformer	**6a-1**	**6a-2**	**6a-3**	**6b-1**	**6b-2**	**6b-3**
G, kcal·mol^{-1}	0.0	0.1	1.6	0.0	0.3	2.5
Rel. content	0.51	0.43	0.06	0.62	0.37	0.01

[a] Calculated according to the equation $K=e^{(-\Delta G/RT)}$

^{13}C NMR chemical shifts were calculated for each conformer (Table S2), averaged for pairs of atoms 1-4, 2-3, 5-8, 6-7, 18(17)-21(19,20) and weight-average chemical shifts were calculated for **6a** and **6b**, taking into account the abundance of conformers (Tables S3,S4).

Table S2. Calculated ^{13}C NMR chemical shifts of conformers of **6a** and **6b**

C atoms	**6a-1**	**6a-2**	**6a-3**	**6b-1**	**6b-2**	**6b-3**
1	34.73	26.59	30.95	33.98	25.68	26.59
2	37.89	27.79	27.79	33.59	27.95	29.93
3	38.76	33.84	33.40	33.69	33.17	34.45
4	31.68	26.13	27.47	33.94	25.87	25.09
5	42.07	41.22	36.16	35.78	40.43	40.99
6	69.79	68.37	70.75	67.67	67.26	67.35
7	69.31	66.45	65.04	67.69	68.36	65.97
8	41.81	40.77	36.77	35.84	40.06	45.56
18(17)	65.01	60.34	59.85	64.86	63.10	61.62
21(19)(20)	55.35	57.09	54.31	64.82	62.15	55.83

Table S3. Averaged for atom pairs and weight-average ^{13}C NMR chemical shifts of conformers of **6a** and experimental ^{13}C NMR chemical shifts

C atoms	**6a-1**	**6a-2**	**6a-3**	weight-average	**6a** (exp)	**6b** (exp)
1,4	33.21	26.36	29.21	30.02	22.8	25.1
2,3	38.33	30.82	30.60	34.63	25.9	25.4
5,8	41.94	41.00	36.46	41.21	33.8	32.3
6,7	69.55	67.41	67.90	68.53	60.3	58.7
17,21	60.18	59.12	57.08	59.54	53.1	52.3

Table S4. Average for atom pairs and weight-average ^{13}C NMR chemical shifts of conformers of **6b** and experimental ^{13}C NMR chemical shifts

C atoms	**6b-1**	**6b-2**	**6b-3**	weight-average	**6a** (exp)	**6b** (exp)
1,4	33.96	25.78	25.84	30.85	22.8	25.1
2,3	33.64	30.56	32.19	32.49	25.9	25.4
5,8	35.81	40.25	43.28	37.53	33.8	32.3
6,7	67.68	67.81	66.66	67.72	60.3	58.7
17,21	64.84	62.63	58.73	63.96	53.1	52.3

Though the calculated ^{13}C NMR chemical shifts did not exactly coincide with experimental chemical shifts, the linear correlation method allowed to attribute the configuration of each of isolated isomers of bis(oxirane) **6** (Tables S5,S6).

Table S5. Linear correlation of ^{13}C NMR chemical shifts for **6a**

Coefficients	A	B	R^2	RMS
**6a** (weight-average)/ **6a** (experimental)	**0.987**	**8.181**	**0.996**	**1.01**
**6a** (weight-average)/ **6b** (experimental)	1.04	6.46	0.981	5.01

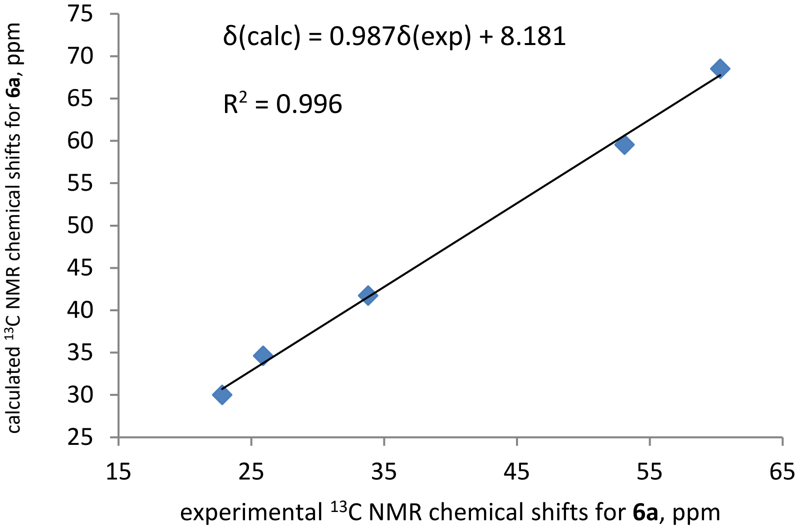

Table S6. Linear correlation of ^{13}C NMR chemical shifts for **6b**

Coefficients	A	B	R^2	RMS
**6b** (weight-average)/ **6a** (experimental)	1.06	4.87	0.977	7.42
**6b** (weight-average)/ **6b** (experimental)	**1.13**	**2.57**	**0.989**	**3.41**

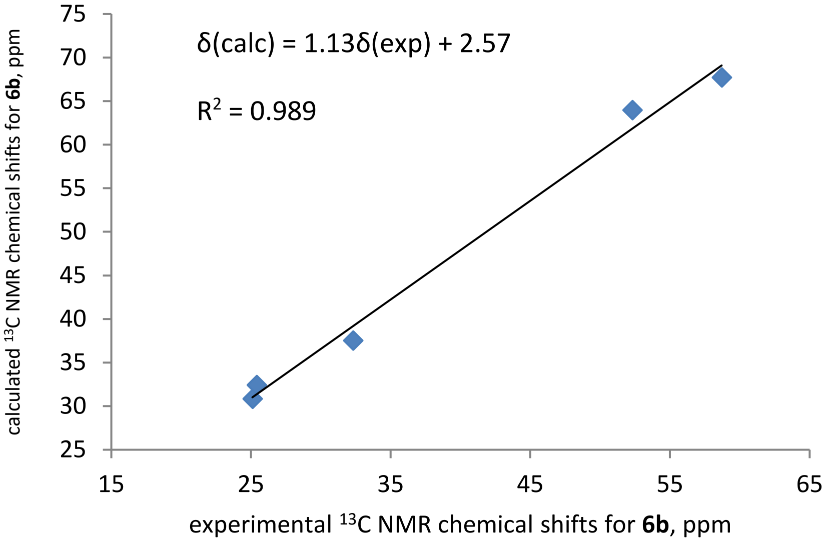

Table S7. DFT optimized structures of **6a** and **6b**

6a-1

cartesian

set=L1

6	1.79963260	0.15878287	-1.08597469
6	1.89093158	-1.25880544	-0.49018592
6	1.50555596	-1.45234631	0.98802497
6	0.01683008	-1.61566962	1.33844543
6	-0.83943121	-0.34070610	1.45503467
6	-1.40698903	0.20617975	0.14870925
6	-0.57105541	1.18305640	-0.65585175
6	0.43886476	0.62048797	-1.64046427
1	2.50731202	0.20091718	-1.93245090
1	2.17214286	0.89454685	-0.34807031
1	-0.03437108	-2.12204180	2.31816051
1	-0.47063328	-2.29496099	0.61620487
1	2.94349335	-1.57887240	-0.58875160
1	1.30427004	-1.95722427	-1.11664628
1	1.93810965	-0.62884520	1.58938181
1	2.01429230	-2.37044751	1.33266223
6	-2.87260224	0.26810271	-0.03247538
8	-2.10413356	-0.77295767	-0.66137079
1	-3.28498447	1.02800083	-0.70437603
1	-3.51822430	-0.05384196	0.79619450
6	-0.39129501	2.54625509	-0.11919515
8	-1.31479808	2.31600432	-1.19025760
1	0.51552293	3.10976587	-0.37966782
1	-0.83915828	2.80743946	0.85004135
1	-0.04657081	-0.20688666	-2.18825582
1	0.60976535	1.42878182	-2.37109306
1	-0.26587291	0.45616832	1.96201585
1	-1.69660383	-0.57088350	2.11021194

$end

Energy = -540.52504326

6a-2

cartesian

set=L1

6	1.72334779	-0.26165436	-1.11651828
6	1.34105429	-1.57593357	-0.39567061
6	1.33304690	-1.49448697	1.14240204
6	0.56443461	-0.31808711	1.76122226
6	-0.93957908	-0.24842665	1.45568188
6	-1.33828710	0.08994151	0.02243619
6	-0.48970804	1.09316714	-0.74420784
6	0.54432059	0.53397336	-1.70877541
1	2.39174556	-0.49794599	-1.96212312
1	2.31467576	0.38458767	-0.44129566
1	1.03571642	0.63387107	1.45764086
1	0.68234612	-0.37079843	2.85816679
1	2.05909105	-2.36492752	-0.67800274
1	0.35741970	-1.91631407	-0.75893116
1	2.37827463	-1.43119347	1.49824413
1	0.92808187	-2.44211512	1.54360447
6	-2.75826056	-0.03906826	-0.35869122
8	-1.80105937	-1.01996550	-0.79276272
6	-0.33786551	2.45630259	-0.19648571
8	-1.22343035	2.22094807	-1.29730211
1	0.57493998	3.02413067	-0.42513881
1	-0.82014705	2.71658140	0.75593352
1	-1.38776069	0.51931770	2.11333993
1	-1.42766430	-1.20697206	1.70797307
1	-0.00255799	-0.11752729	-2.41337933
1	0.93038933	1.38965964	-2.28820358
1	-3.15299040	0.62571921	-1.13502405
1	-3.47957416	-0.38278368	0.39586722

$end

Energy = -540.52718472

6a-3

cartesian

set=L1

6	1.66079907	-0.12806939	-1.36748532
6	1.39294258	-1.40568013	-0.55756574
6	1.66452634	-1.35106940	0.95327735
6	0.79675670	-0.39261515	1.79439088
6	-0.71941943	-0.41559614	1.48550674
6	-1.20512632	0.71134253	0.58321969
6	-0.72573790	0.77131462	-0.85566349
6	0.75355898	1.08545060	-1.06824215
1	1.55701345	-0.38606354	-2.43658069
1	2.71327691	0.18156876	-1.23233101
1	1.15750409	0.64532187	1.70929080
1	0.94053055	-0.66375445	2.85401944
1	2.01978765	-2.21069494	-0.98137615
1	0.34880029	-1.72453205	-0.72986606
1	2.72863683	-1.10355921	1.12406776
1	1.52378985	-2.37486834	1.34687783
8	-1.19747845	2.01058302	1.23002073
6	-2.46462270	1.40352855	0.91668695
1	-3.04868748	1.04942770	1.77725862
1	-3.02514660	1.88221129	0.10675970
8	-1.62551529	1.47257670	-1.75332664
6	-1.50038812	0.05214657	-1.88893580
1	-1.27805661	-0.31493965	2.43010385
1	-1.01201466	-1.39005945	1.05265305
1	0.80188804	1.78669327	-1.91762864
1	1.11391000	1.64334367	-0.18994552
1	-2.37869845	-0.53619155	-1.58746337
1	-0.99282932	-0.29781576	-2.79772280

$end

Energy = -540.5245888600

6b-1

cartesian

set=L1

6	1.80409115	0.03277034	-1.25762373
6	1.76322997	-1.24220672	-0.39292783
6	1.55966952	-1.04693816	1.13367062
6	0.16745816	-1.38810503	1.69918513
6	-0.91820241	-0.30423443	1.58392410
6	-1.29073128	0.06356109	0.15895424
6	-0.41558880	1.11156849	-0.54010237
6	0.45242387	0.62830859	-1.68788183
1	2.36237523	-0.19224347	-2.18370141
1	2.37994496	0.81239532	-0.72739024
1	0.27517480	-1.61807471	2.77410765
1	-0.20350910	-2.31099430	1.21833523
1	2.72893930	-1.75585719	-0.53822171
1	0.99299449	-1.93152354	-0.78518845
1	1.83050488	-0.01262403	1.41518512
1	2.28223924	-1.69914060	1.65338043
6	-2.68568050	-0.08735658	-0.30991341
8	-1.68515028	-1.04790951	-0.68274601
1	-3.05197383	0.55456927	-1.12297233
1	-3.45371030	-0.44431546	0.39000479
8	0.21600485	2.06054352	0.35430012
6	-0.85682453	2.52234358	-0.48147813
1	-0.12815527	-0.12318305	-2.24937605
1	0.64408603	1.47750806	-2.36573774
1	-0.58403247	0.61837879	2.08804447
1	-1.82397016	-0.65992716	2.10390140
1	-1.79024399	2.77239819	0.04146990
1	-0.57136352	3.21028869	-1.28920198

$end

Energy = -540.52480541

6b-2

cartesian

set=L1

6	1.79567815	-0.24308076	-1.02401611
6	1.45542962	-1.54148291	-0.25464791
6	1.42074360	-1.40708177	1.28124406
6	0.64309259	-0.21390742	1.85605810
6	-0.86325799	-0.15498451	1.55506193
6	-1.26515595	0.05999505	0.10160318
6	-0.44285103	1.05709652	-0.71077276
6	0.59583289	0.48536749	-1.66231559
1	2.48733466	-0.48486824	-1.84928700
1	2.34246764	0.46025438	-0.37033545
1	1.10280394	0.73135627	1.52398548
1	0.75800395	-0.23142701	2.95466145
1	2.20913425	-2.31027201	-0.49762108
1	0.49286677	-1.93678593	-0.61725117
1	2.46042587	-1.32501075	1.64931038
1	1.01856799	-2.34601776	1.70550710
6	-2.67156977	-0.19245851	-0.28122644
8	-1.64244408	-1.12387450	-0.64765485
8	-0.03568575	2.23164905	0.03487002
6	-1.02182798	2.38902601	-0.99608458
1	-1.29168786	0.67973636	2.13826186
1	-1.36295172	-1.08055155	1.89316505
1	0.06868462	-0.21133400	-2.33793509
1	0.97443901	1.31923175	-2.27809455
1	-3.11726509	0.37260767	-1.11025017
1	-3.37945190	-0.54167964	0.48356125
1	-2.03651350	2.62320270	-0.64835008
1	-0.69484292	2.93529403	-1.89144705

$end

Energy = -540.52609296

6b-3

cartesian

set=L1

6	1.72471573	-0.12326188	-0.99802466
6	1.34871280	-1.45201485	-0.30311868
6	1.28662451	-1.37454889	1.23304628
6	0.46903058	-0.21238902	1.81151095
6	-1.02637827	-0.19728135	1.46419109
6	-1.41340647	0.11566340	0.02101602
6	-0.57332660	1.14947739	-0.73152228
6	0.55072558	0.64703439	-1.62982269
1	2.43455012	-0.33194705	-1.81655992
1	2.26875047	0.53012967	-0.29164850
1	0.91954454	0.74849268	1.50557827
1	0.55571506	-0.24388246	2.91187117
1	2.08785517	-2.22682349	-0.56968491
1	0.38293031	-1.80916571	-0.70129338
1	2.31863877	-1.28724246	1.62074814
1	0.89070295	-2.33010248	1.62424841
8	-2.84149602	0.25590488	-0.12989814
6	-2.20085887	-0.85019875	-0.77420235
1	-2.40886133	-1.84388001	-0.35209498
1	-2.17173563	-0.80412740	-1.87170967
6	-0.53773577	2.53482041	-0.21751632
8	-1.33849775	2.20261270	-1.35726547
1	0.34745099	3.15656959	-0.41284819
1	-1.09584616	2.78614733	0.69464850
1	-1.51261506	0.58167978	2.07986343
1	-1.49401781	-1.15547894	1.75296142
1	0.08730801	0.00251962	-2.40021338
1	0.94152013	1.53129292	-2.16226017

$end

Energy = -540.5218889

1.2. Bis(oxiranes) 7a and 7b

Configuration of major meso-diastereomer **7a** and racemate **7b** was determined basing on the difference of symmetry of **7a** and **7b**. In structure **7a** cyclopropane CH_2-groups are not equivalent, and two peaks in ^{13}C NMR spectra at δ_C 6.5 ppm and δ_C 8.8 ppm correspond to carbon atoms of these groups. In structure **7b** corresponding carbon atoms are equivalent and give a signal with chemical shift δ_C 7.3 ppm (Figure S2).

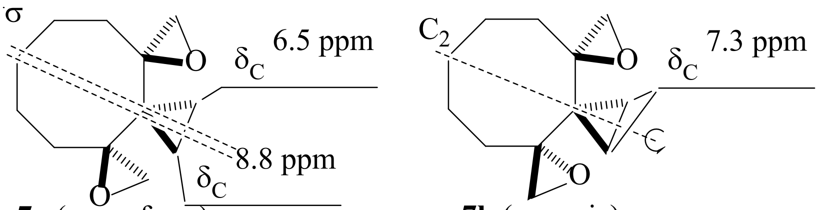

7a (meso form) **7b** (racemic)

Figure S2. Isomers of bis(oxirane) **7**: **7a** (meso form) and **7b** (racemic)

1.3. Bis(oxiranes) 9a and 9b

In diastereomer **9a** protons in each of CH_2-groups are not equivalent, as their chemical surroundings differ depending on the side of the cyclooctane ring. Difference of the chemical shifts of remote from oxirane moieties methylene protons exceeds 2 ppm. Two multiplets, at δ_H 1.55-1.66 ppm and δ_H 1.79-1.92 ppm, correspond to these two types of protons in ^1H NMR spectrum (Figure S3). Diastereomer **9b** possesses higher symmetry, two protons of CH_2-groups must be equivalent at room temperature. All protons of cyclooctane moiety give one narrow multiplet at δ_H 1.62-1.85 ppm (Figure S4).

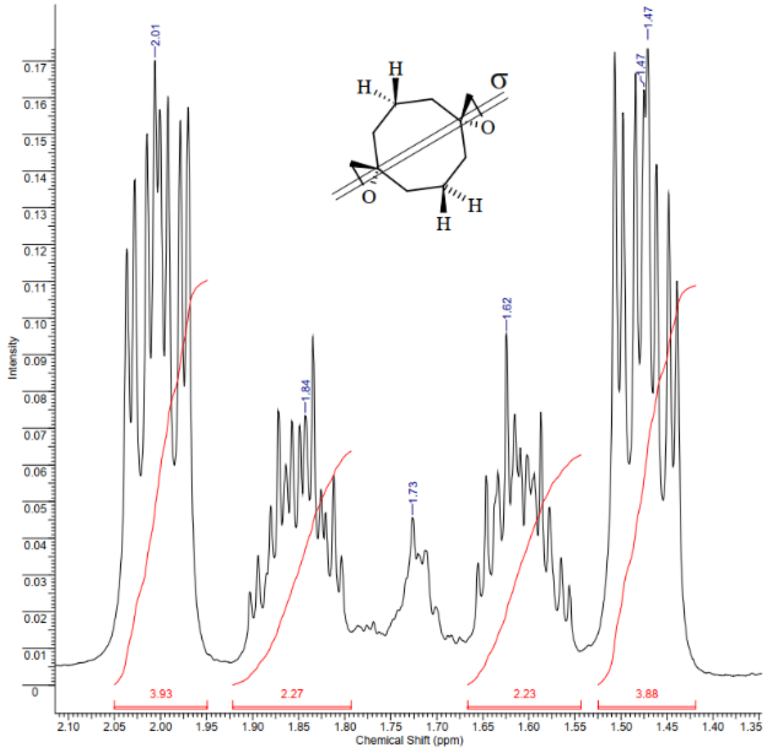

Figure S3. A characteristic region of ^1H NMR spectrum of **9a**

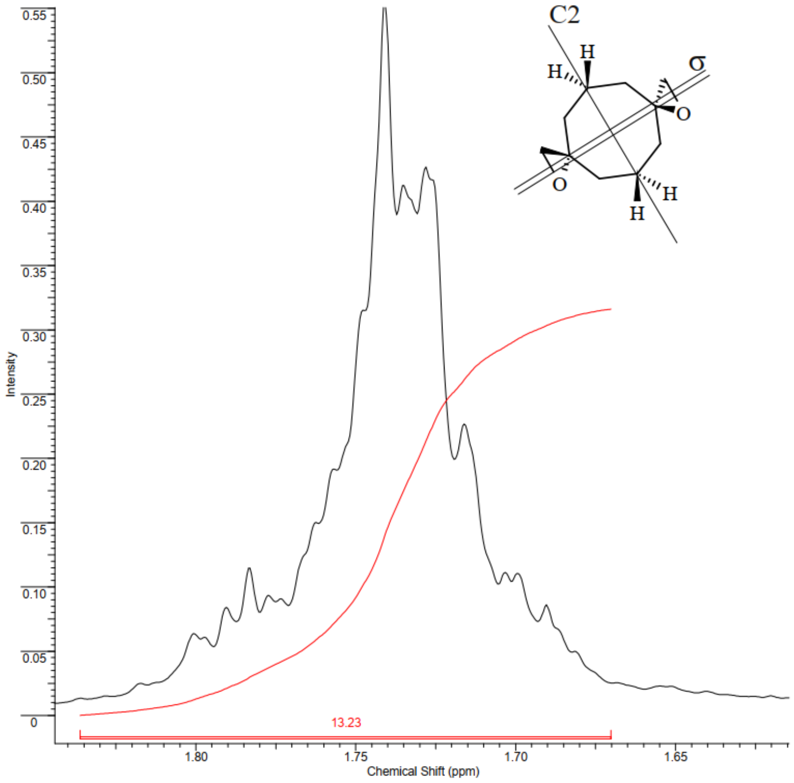

Figure S4. A characteristic region of ^{1}H NMR spectrum of **9b**

2. Optimization of reactions conditions

Table S8. Optimization of conditions of ring-opening of bis(oxirane) **6a**

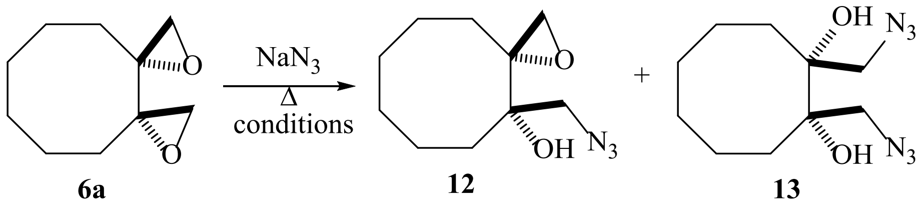

Entry	Solvent	Time, h	eq. NaN3	Products ratio 12:13 according to ^{1}H NMR
1	H_2O	3	8	4 : 1
2		6	16	1 : 1
3		12	16	1 : 4
4		30	16	0 : 1
5	H_2O-dioxane (1:1)	8	16	1 : 1
6		16	16	1 : 4
7	H_2O-CH_3CN (1:1)	8	16	1 : 0
8		16	16	1 : 1
9	H_2O-acetone (1:1)	8	16	1 : 0
10		16	16	1 : 0

Table S9. Optimization of conditions of ring-opening of bis(oxirane) **9a**

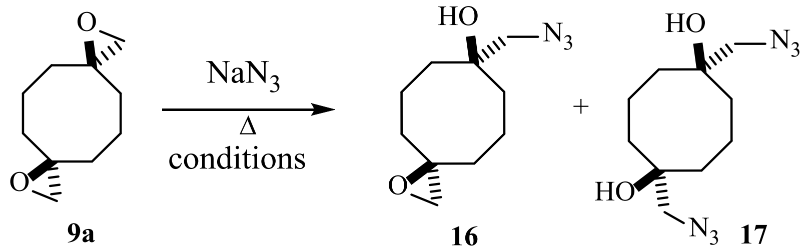

Entry	Solvent	Time, h	eq. NaN3	Products ratio 16:17 according to ^{1}H NMR
1	H_2O	2	4	0.5 : 1
2		3	4	0.1 : 1
3		4	4	0.1 : 1
4		5	4	0.1 : 1
5		3	8	0 : 1

3. Copies of NMR spectra

^{1}H NMR ($CDCl_3$) spectrum of compound **6a**

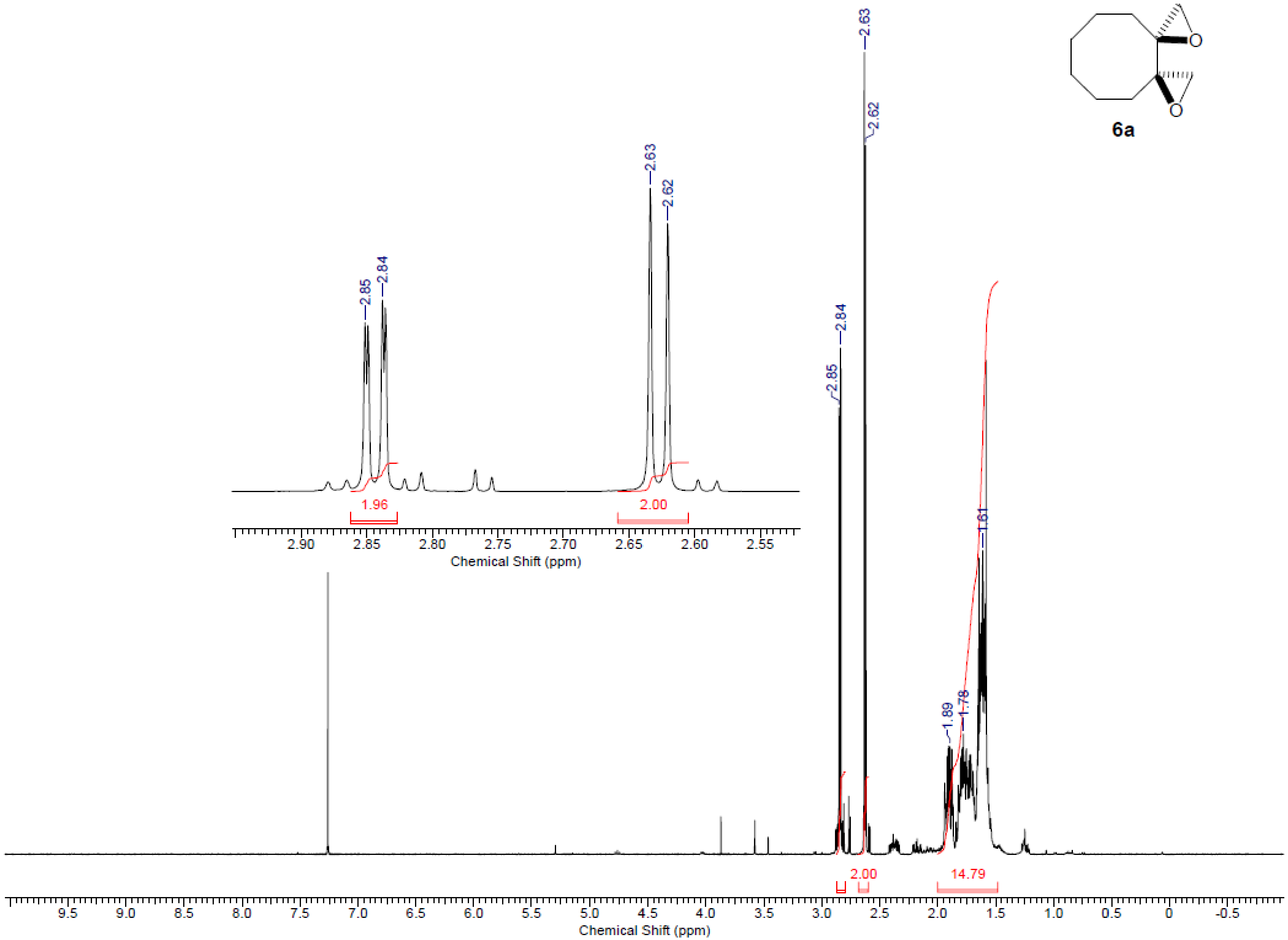

^{13}C NMR ($CDCl_3$) spectrum of compound **6a**

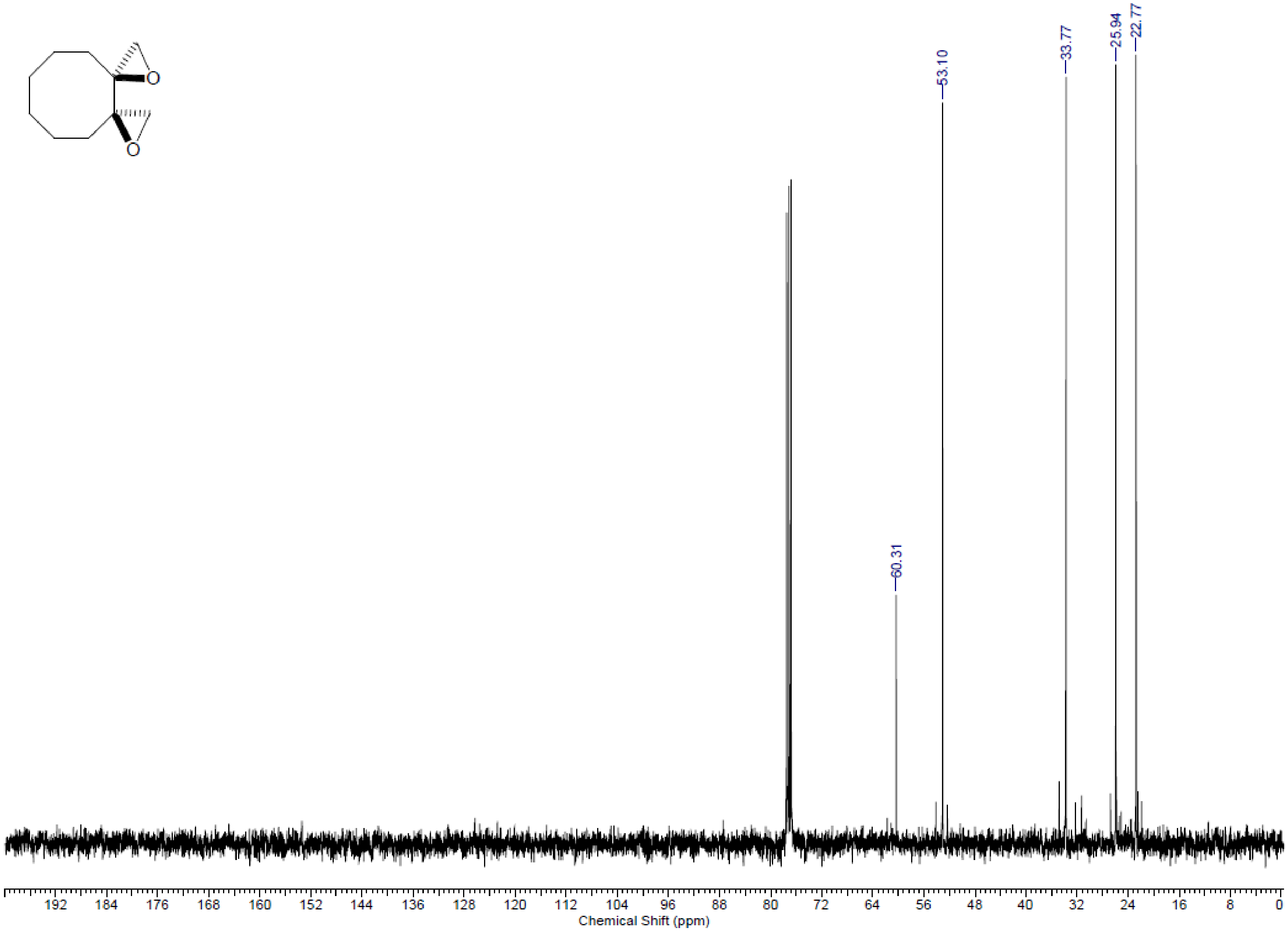

HSQC NMR ($CDCl_3$) spectrum of compound **6a**

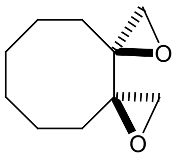

6a

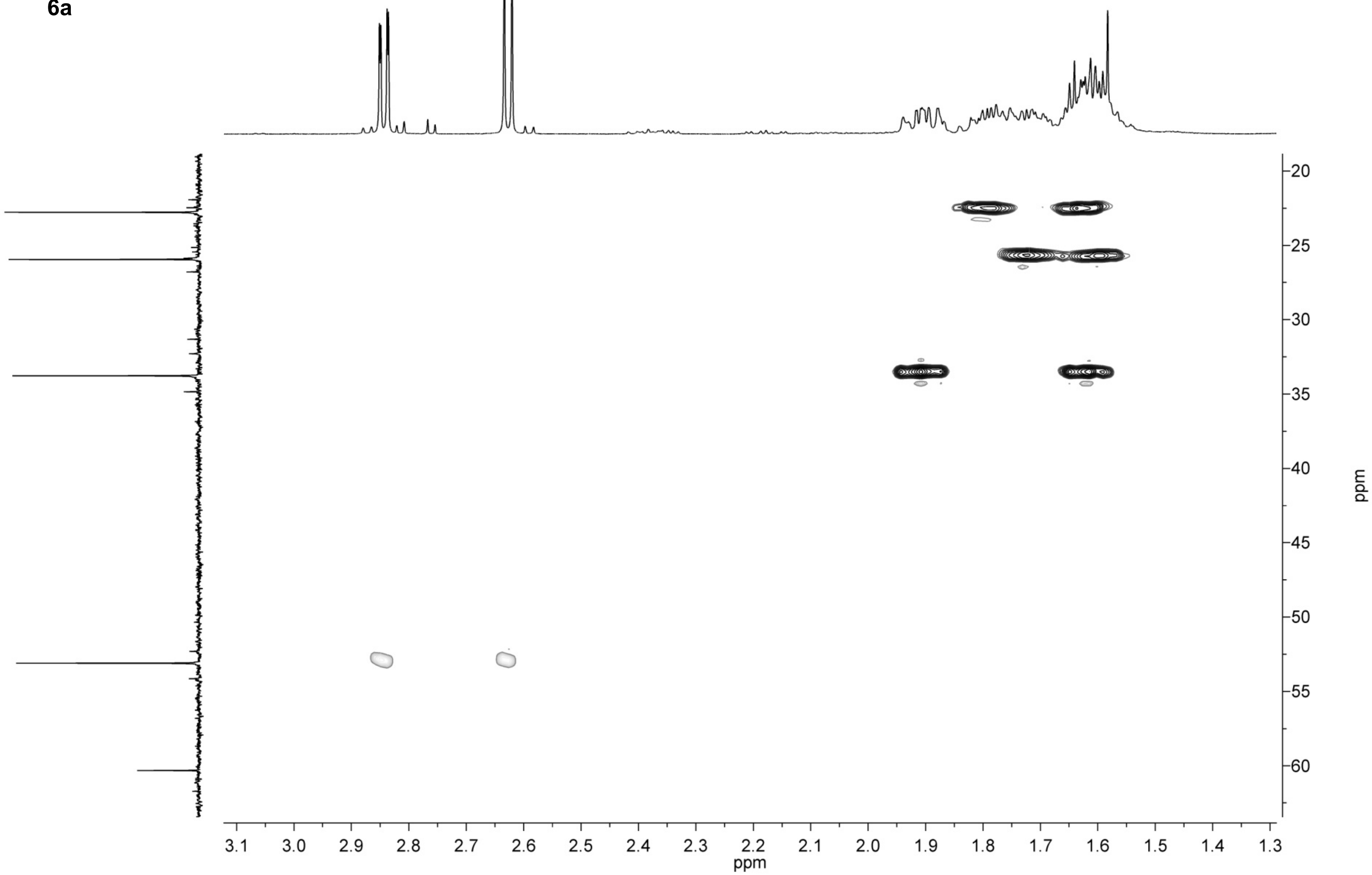

HMBC NMR ($CDCl_3$) spectrum of compound **6a**

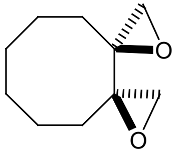

6a

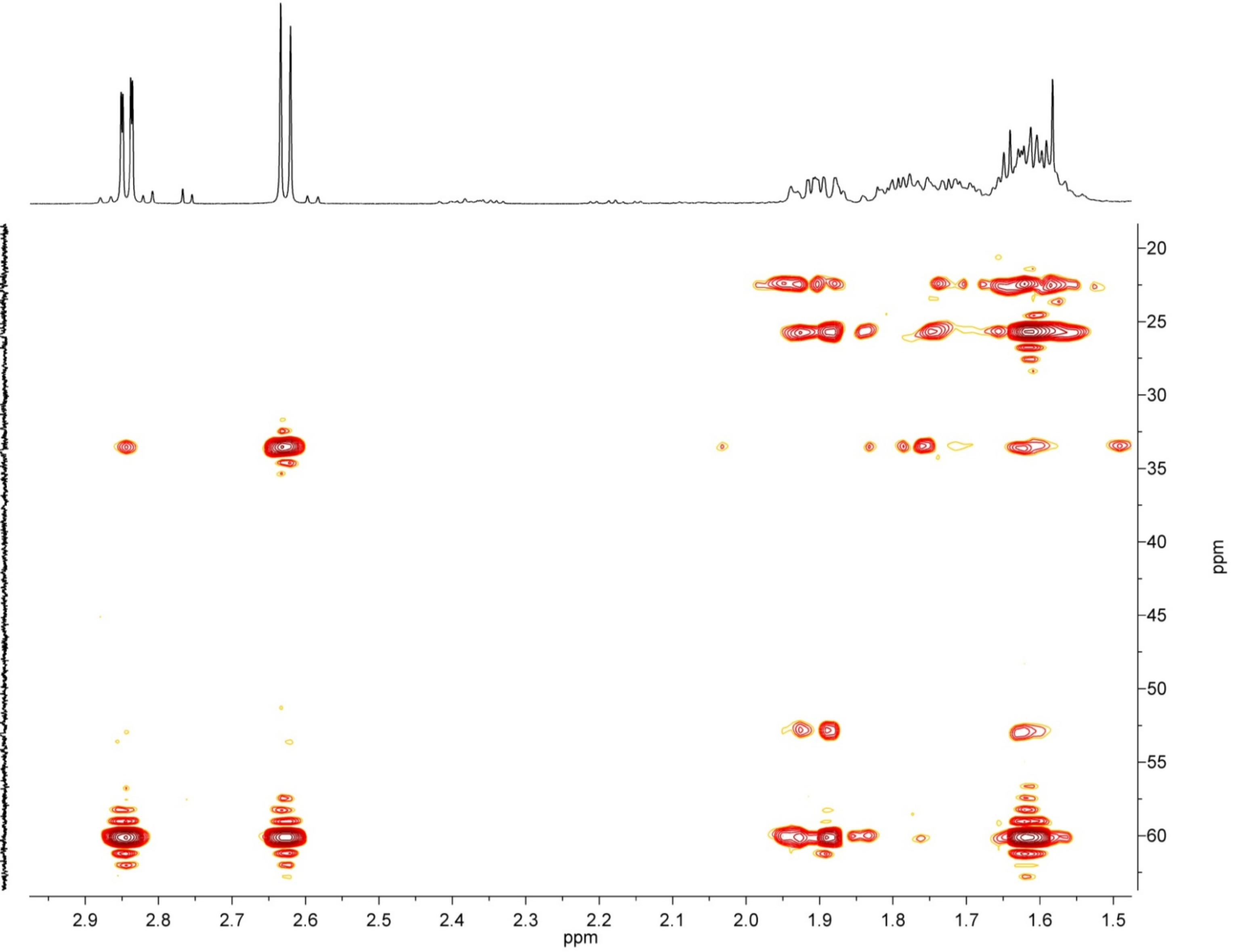

^1H NMR ($CDCl_3$) spectrum of compound **6b**

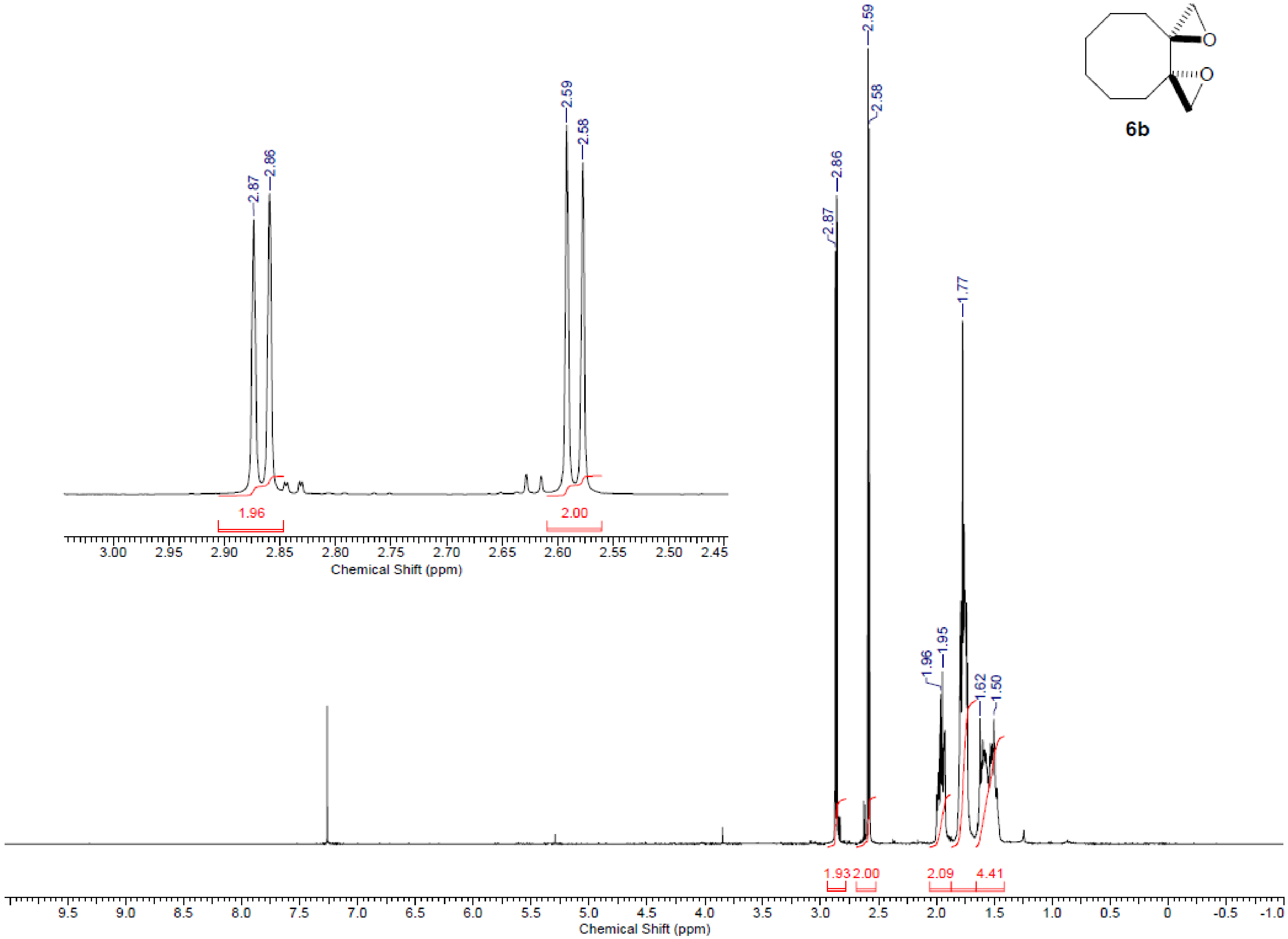

^{13}C NMR ($CDCl_3$) spectrum of compound **6b**

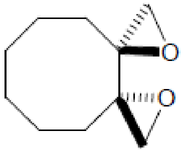

6b

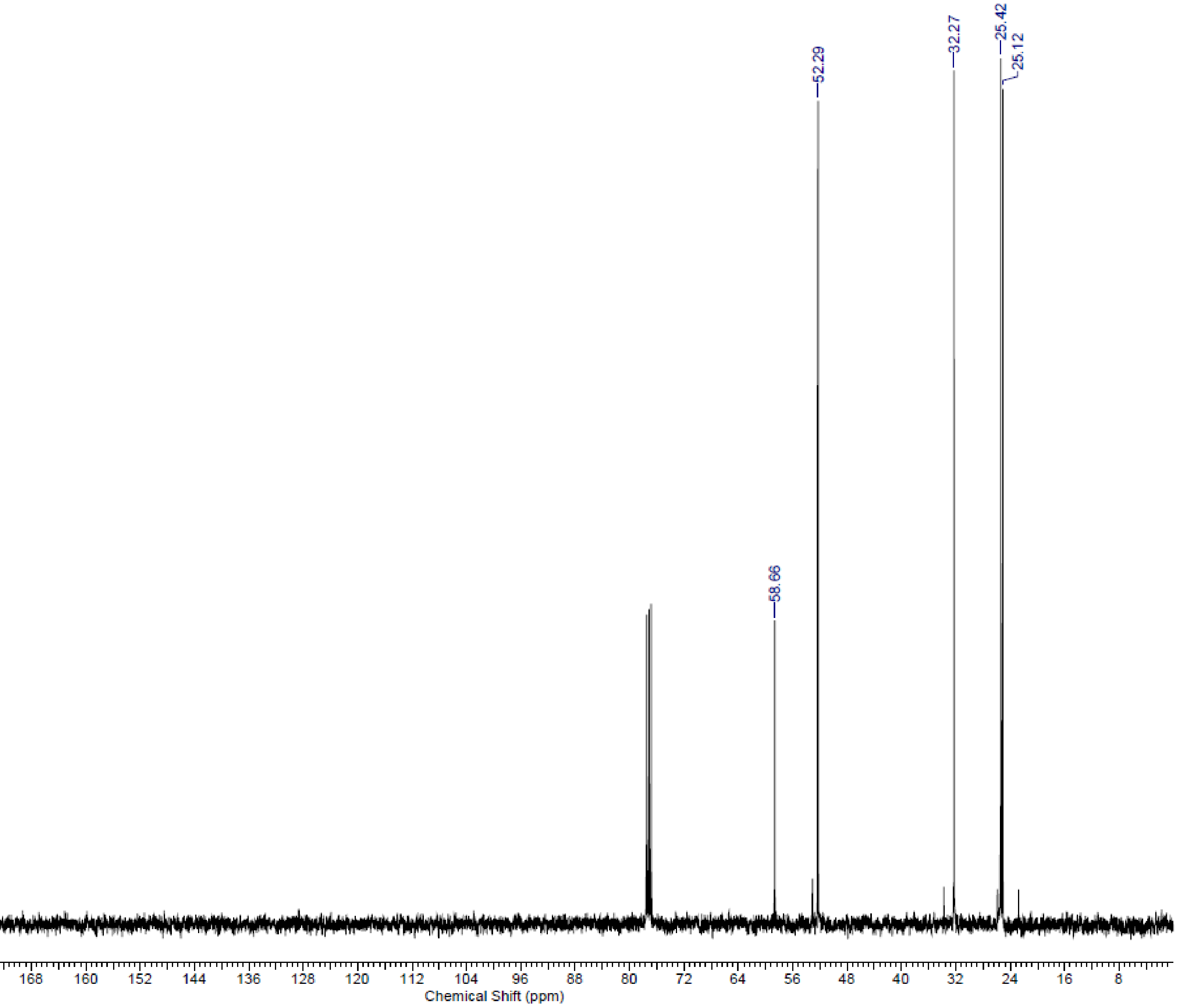

HSQC NMR ($CDCl_3$) spectrum of compound **6b**

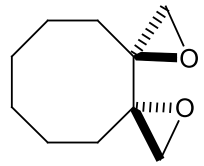

6b

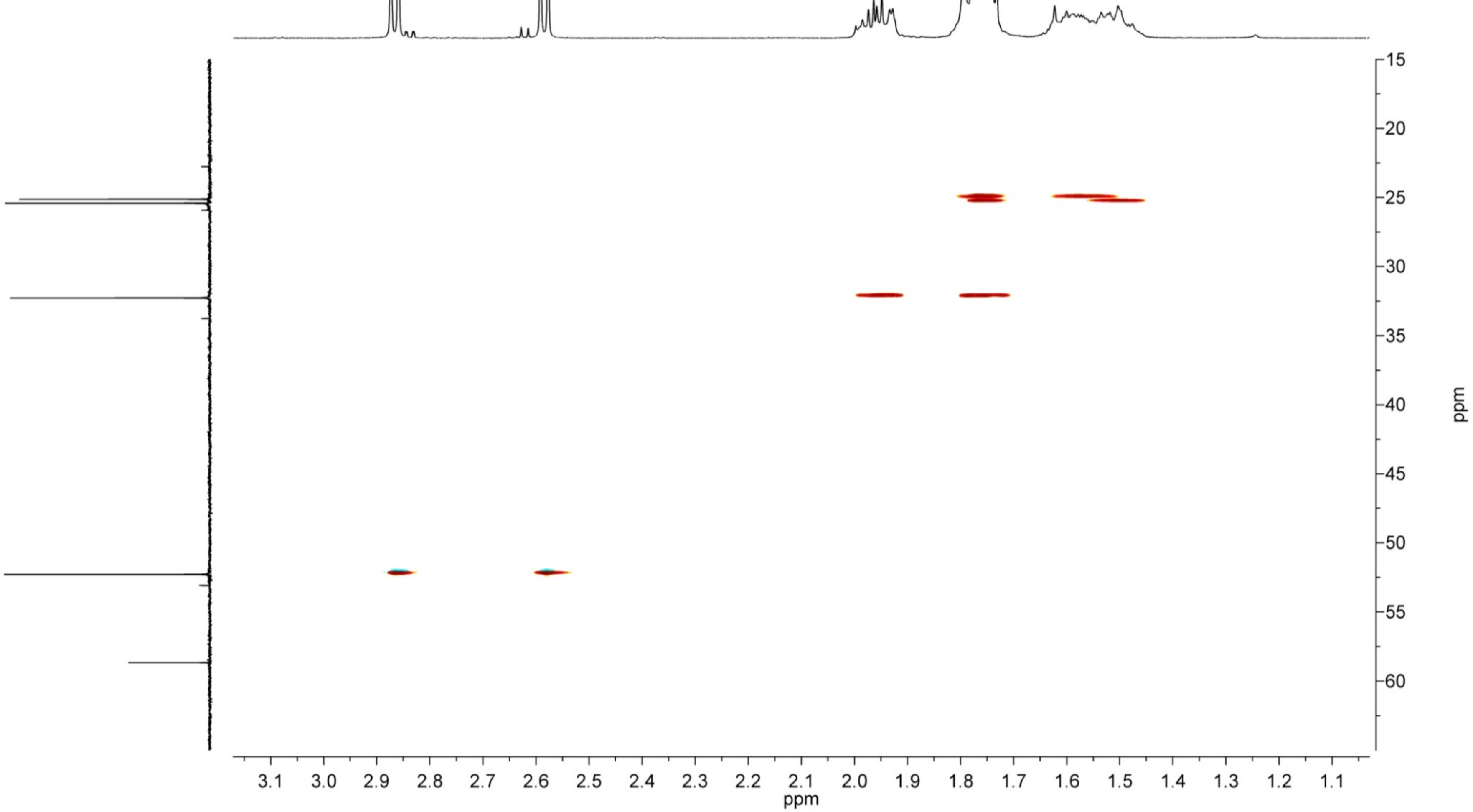

HMBC NMR ($CDCl_3$) spectrum of compound **6b**

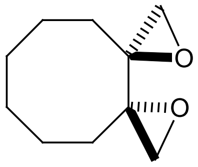

6b

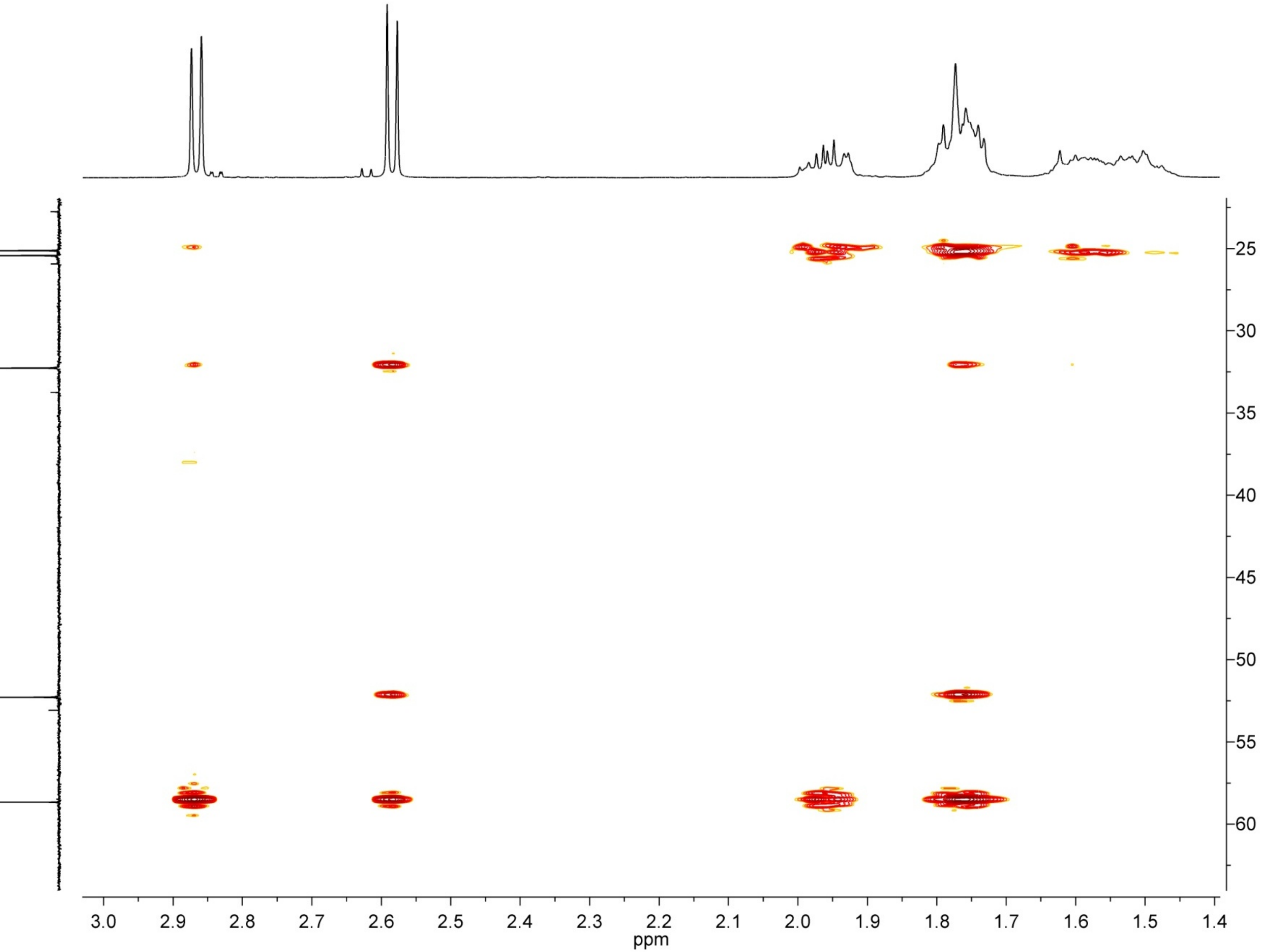

^{1}H NMR ($CDCl_3$) spectrum of compounds **7a,b** (reaction mixture)

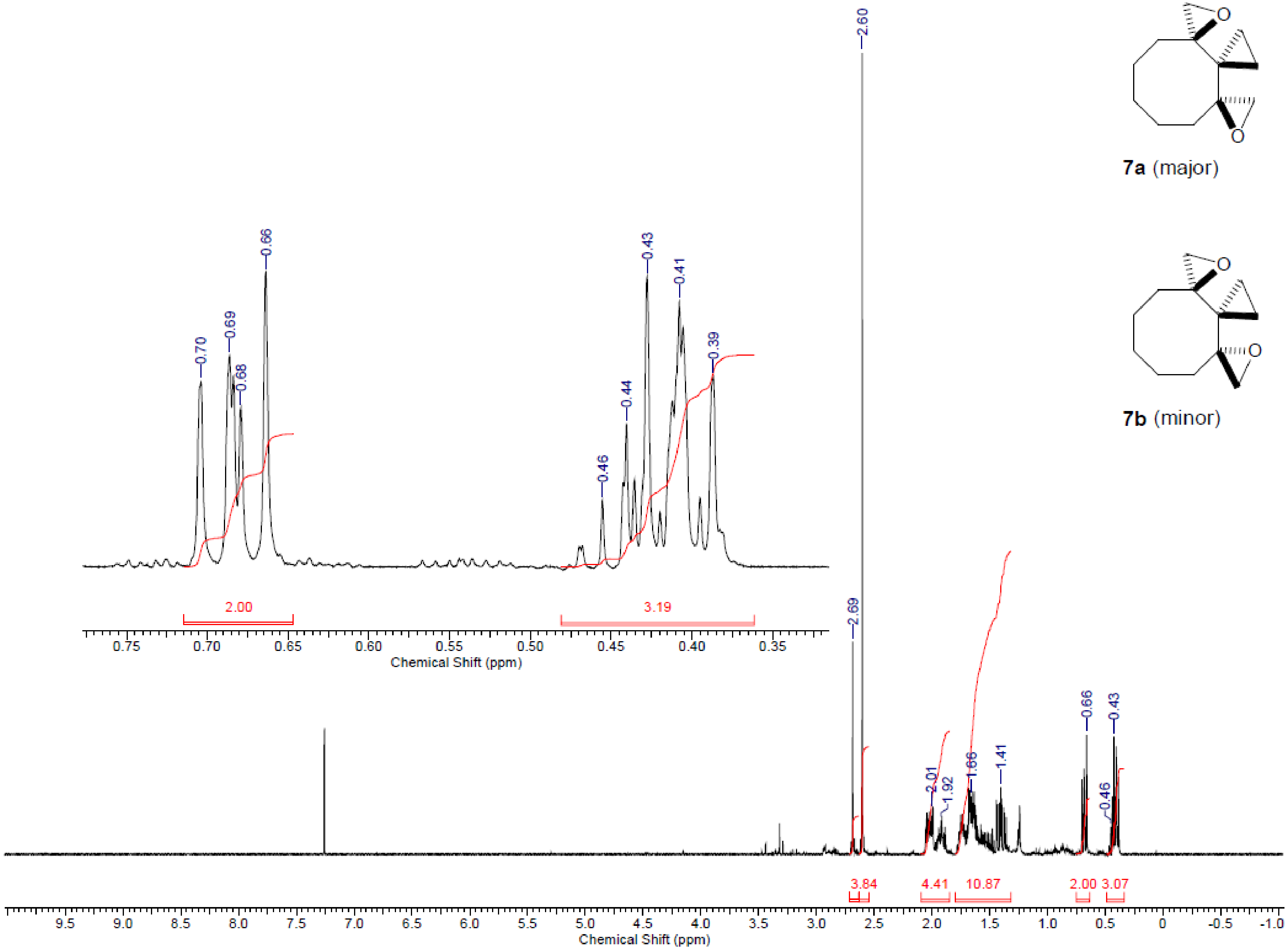

7a (major)

7b (minor)

^{13}C NMR ($CDCl_3$) spectrum of compounds **7a,b** (reaction mixture)

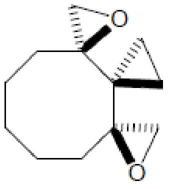

7a (major)

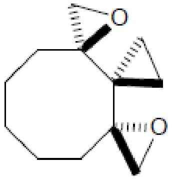

7b (minor)

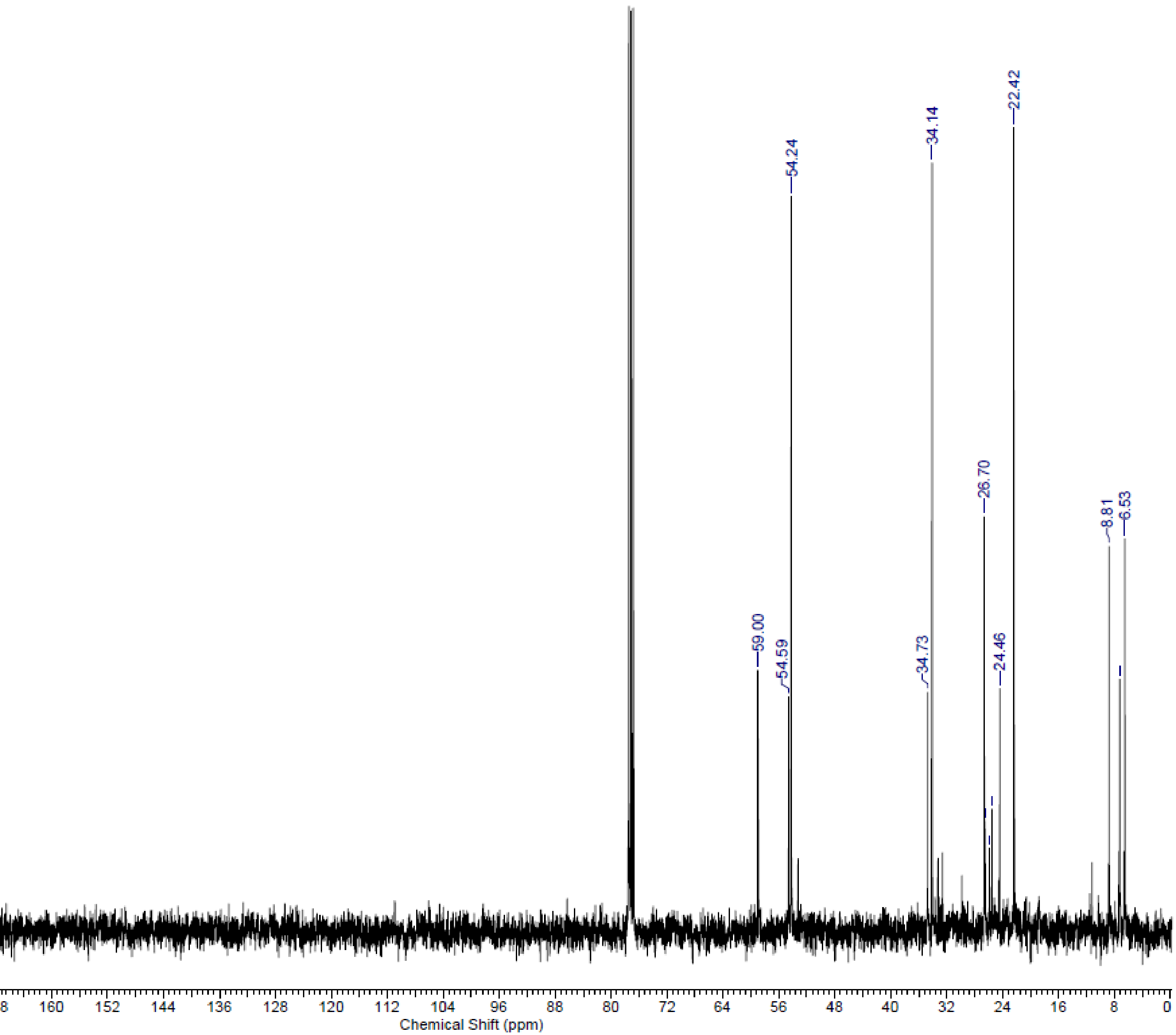

HSQC NMR ($CDCl_3$) spectrum of compounds **7a,b** (reaction mixture)

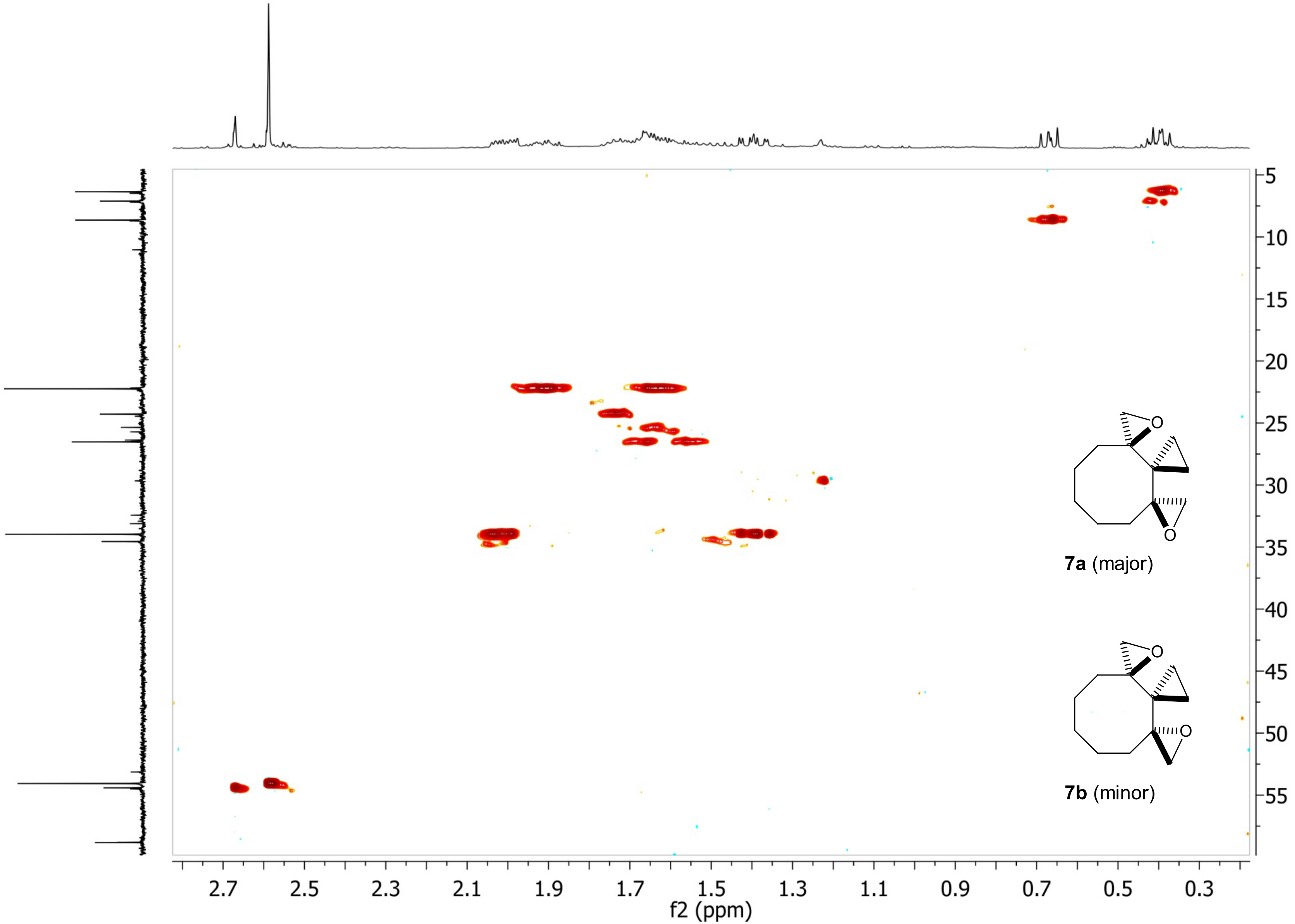

HMBC NMR (CDCl3) spectrum of compounds **7a,b** (reaction mixture)

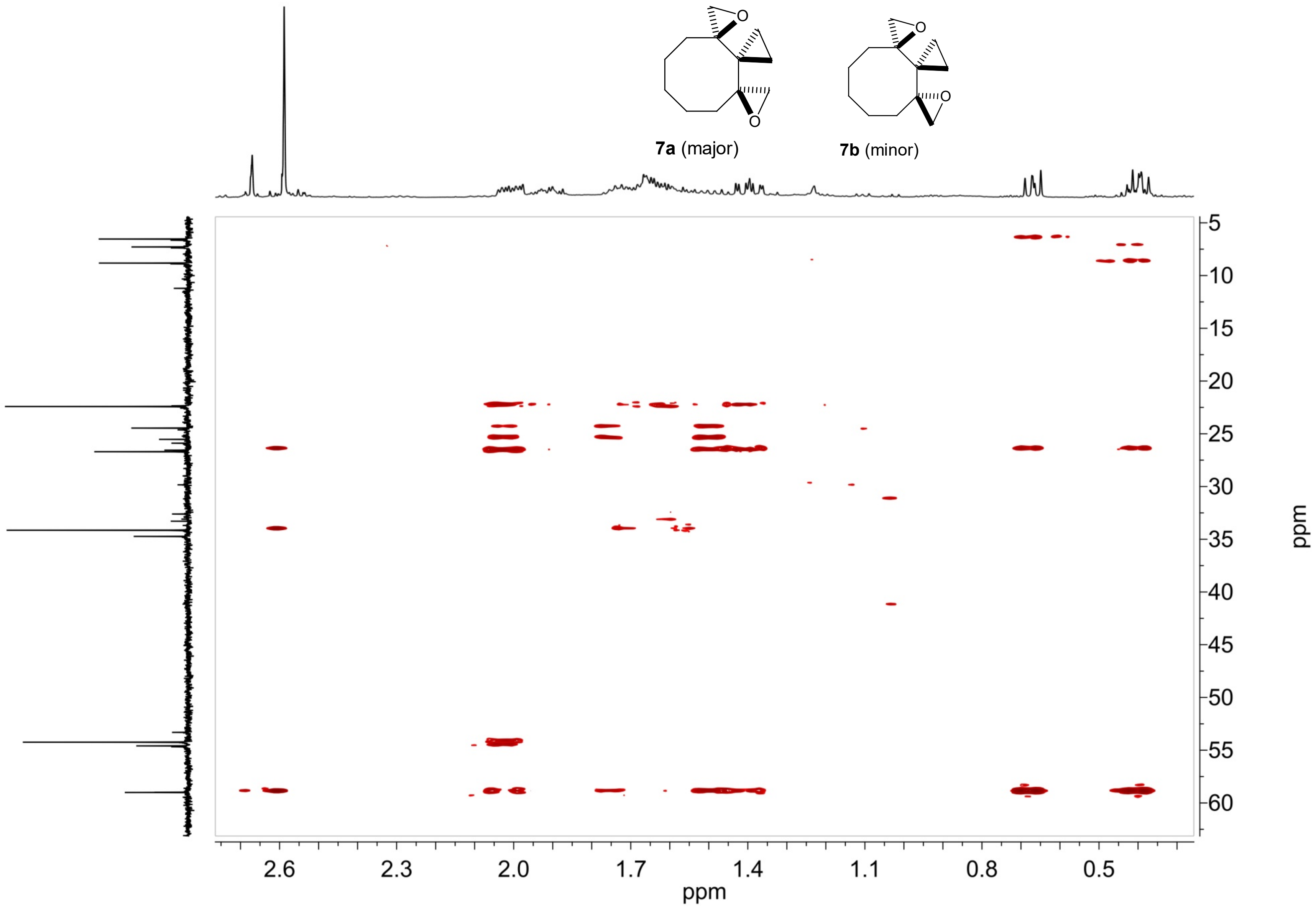

^{1}H NMR ($CDCl_3$) spectra of compound **8**

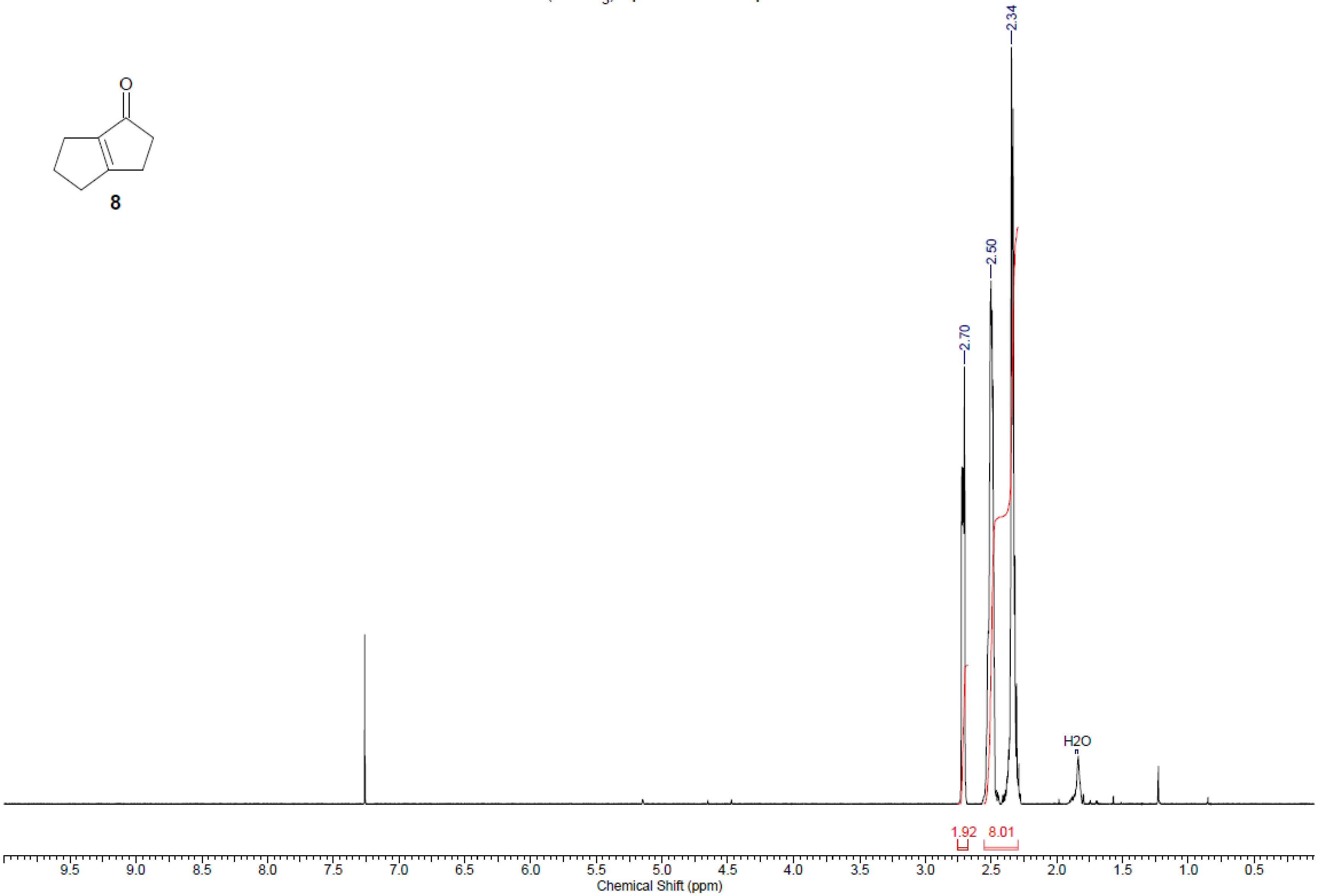

^{13}C NMR ($CDCl_3$) spectra of compound **8**

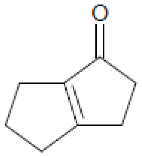

8

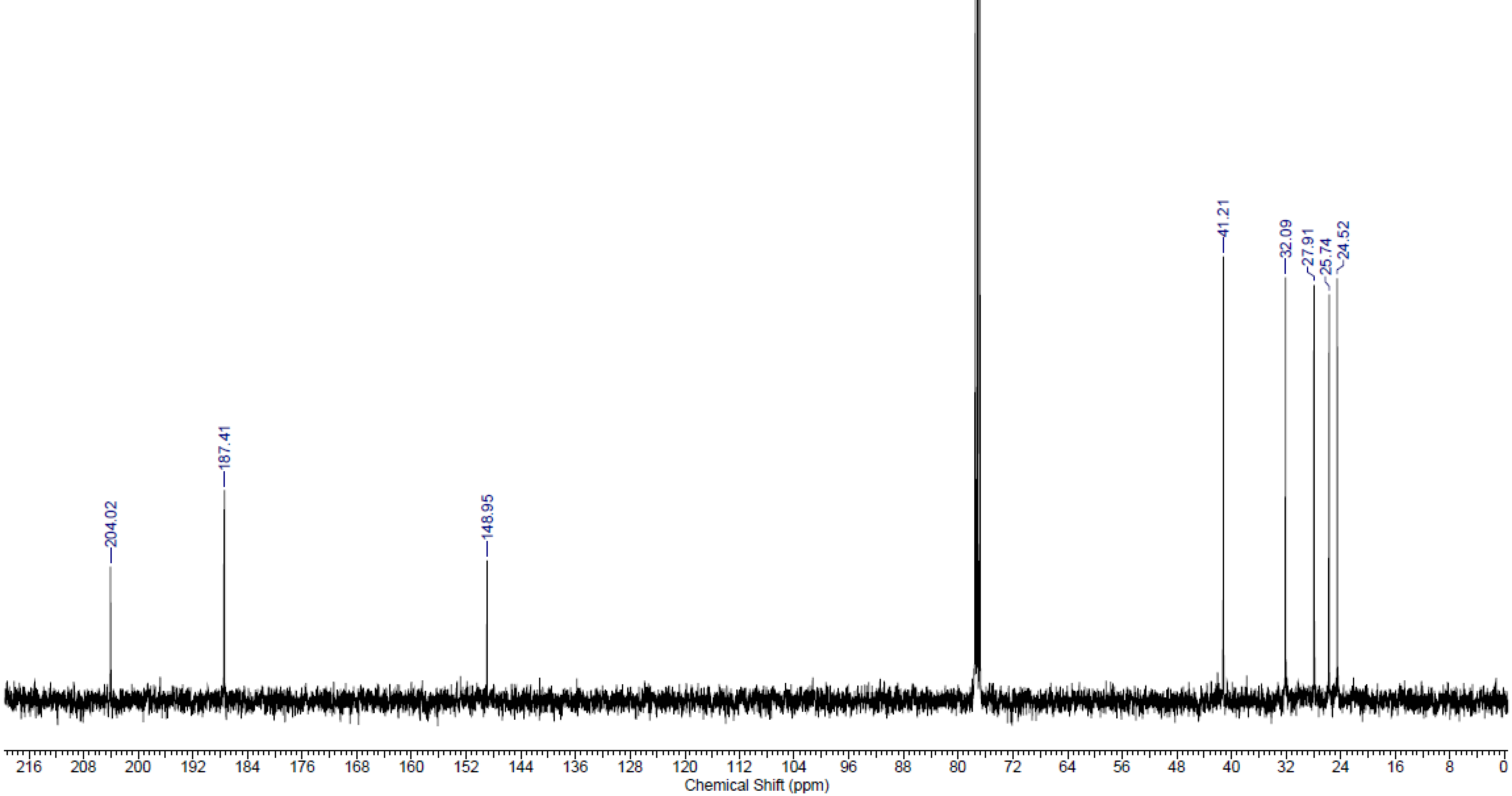

^{1}H NMR ($CDCl_3$) spectrum of compound **9a**

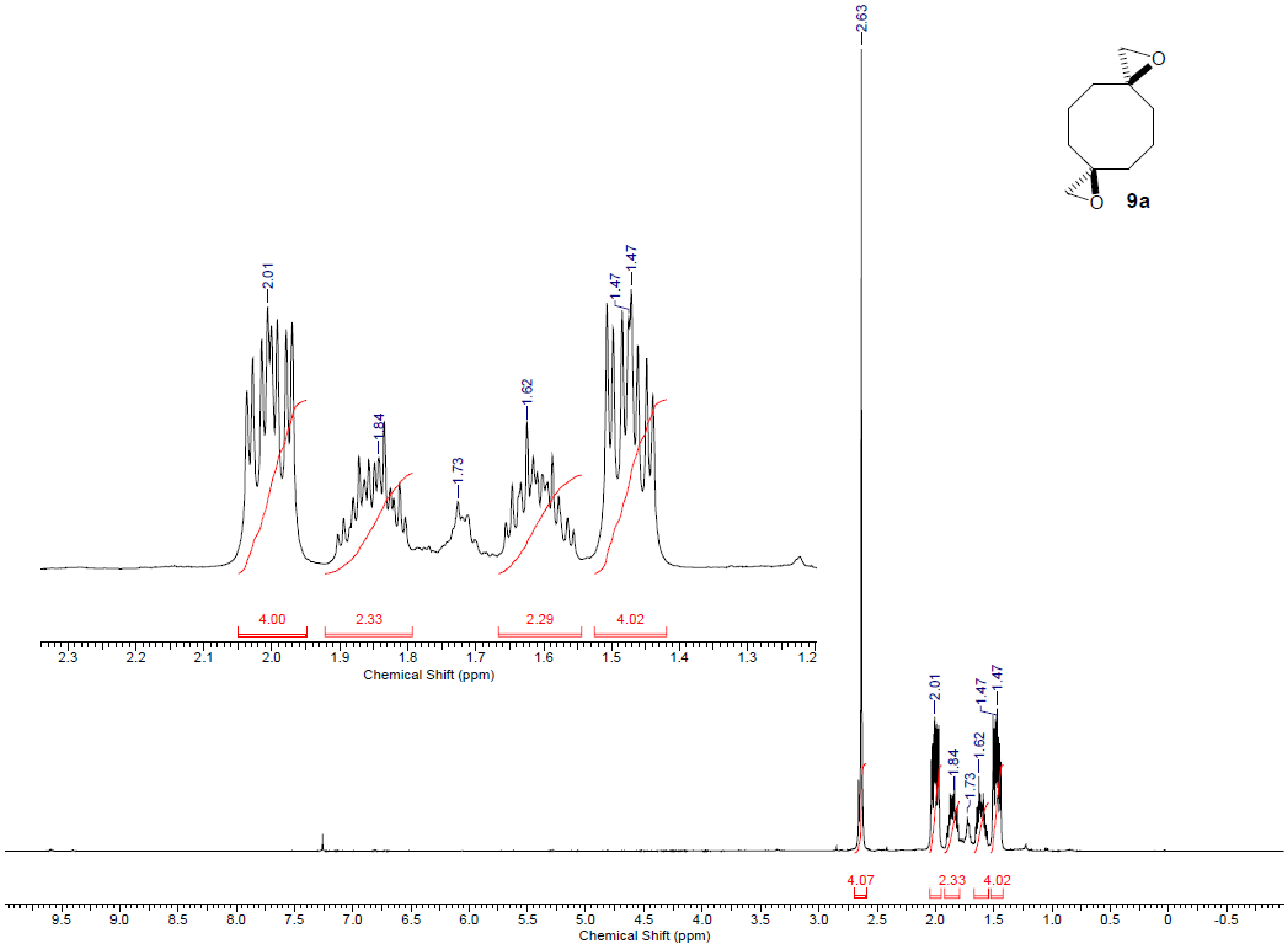

9a

^{13}C NMR ($CDCl_3$) spectrum of compound **9a**

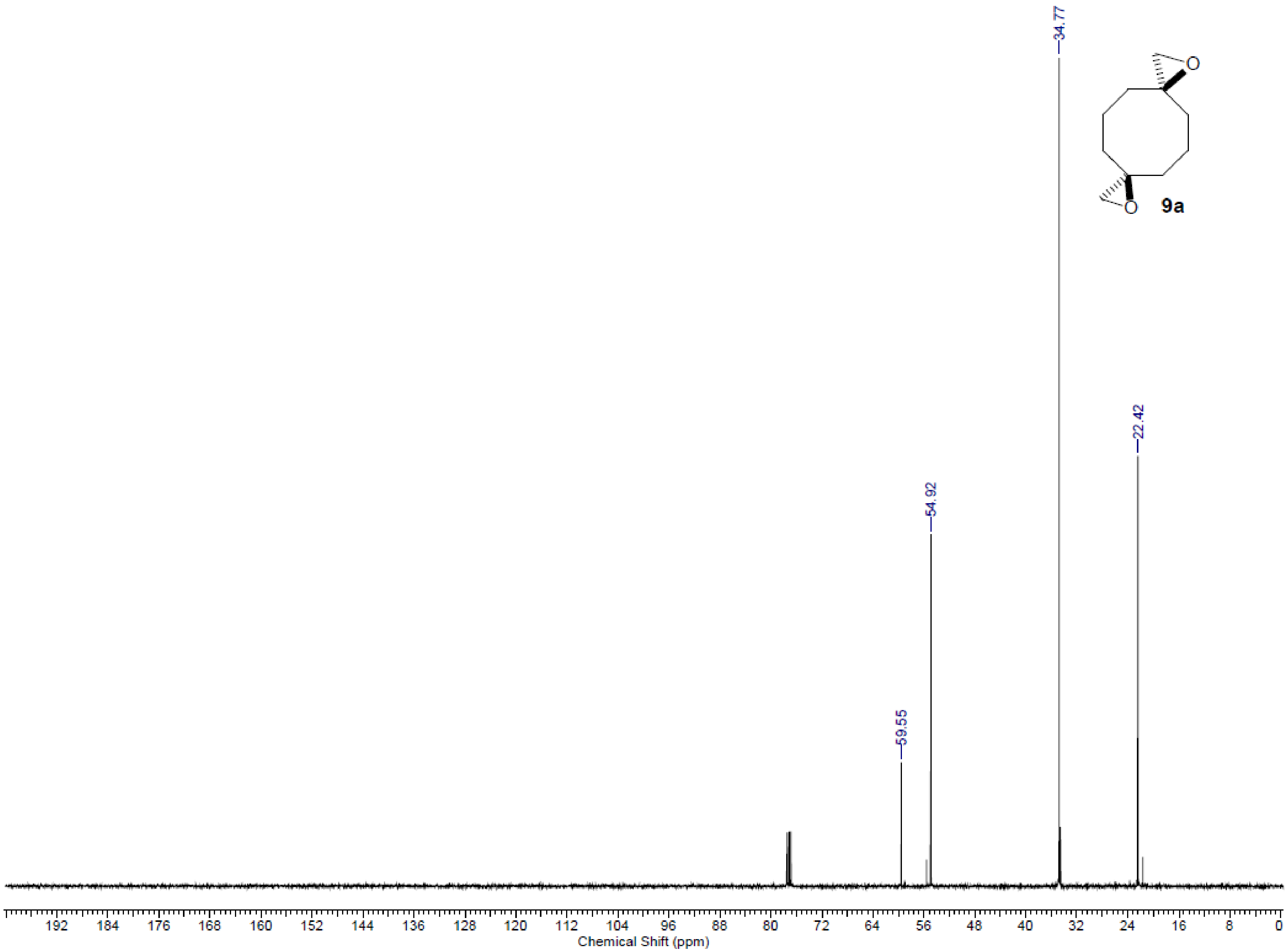

HSQC NMR ($CDCl_3$) spectrum of compound **9a**

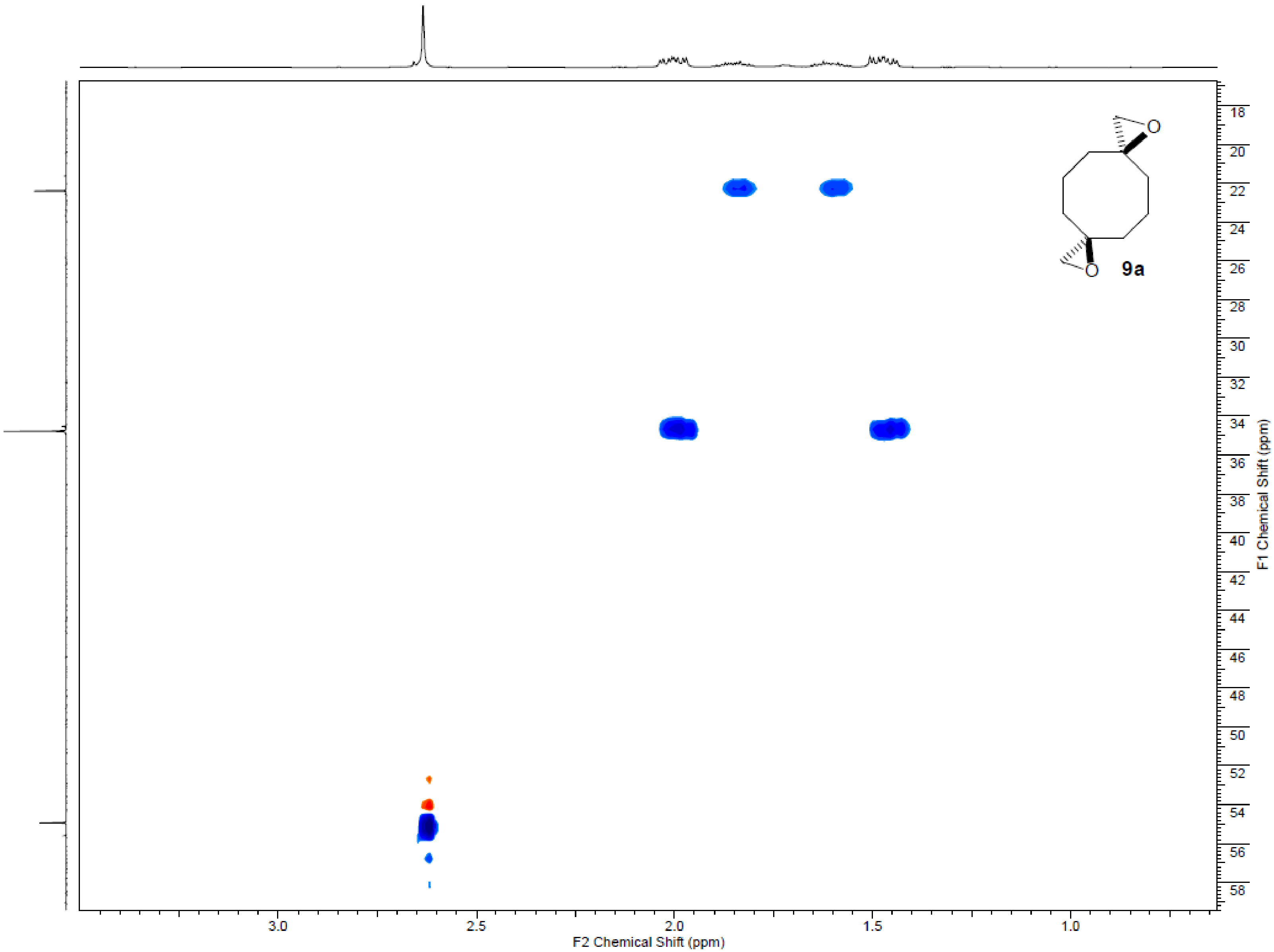

HMBC NMR ($CDCl_3$) spectrum of compound **9a**

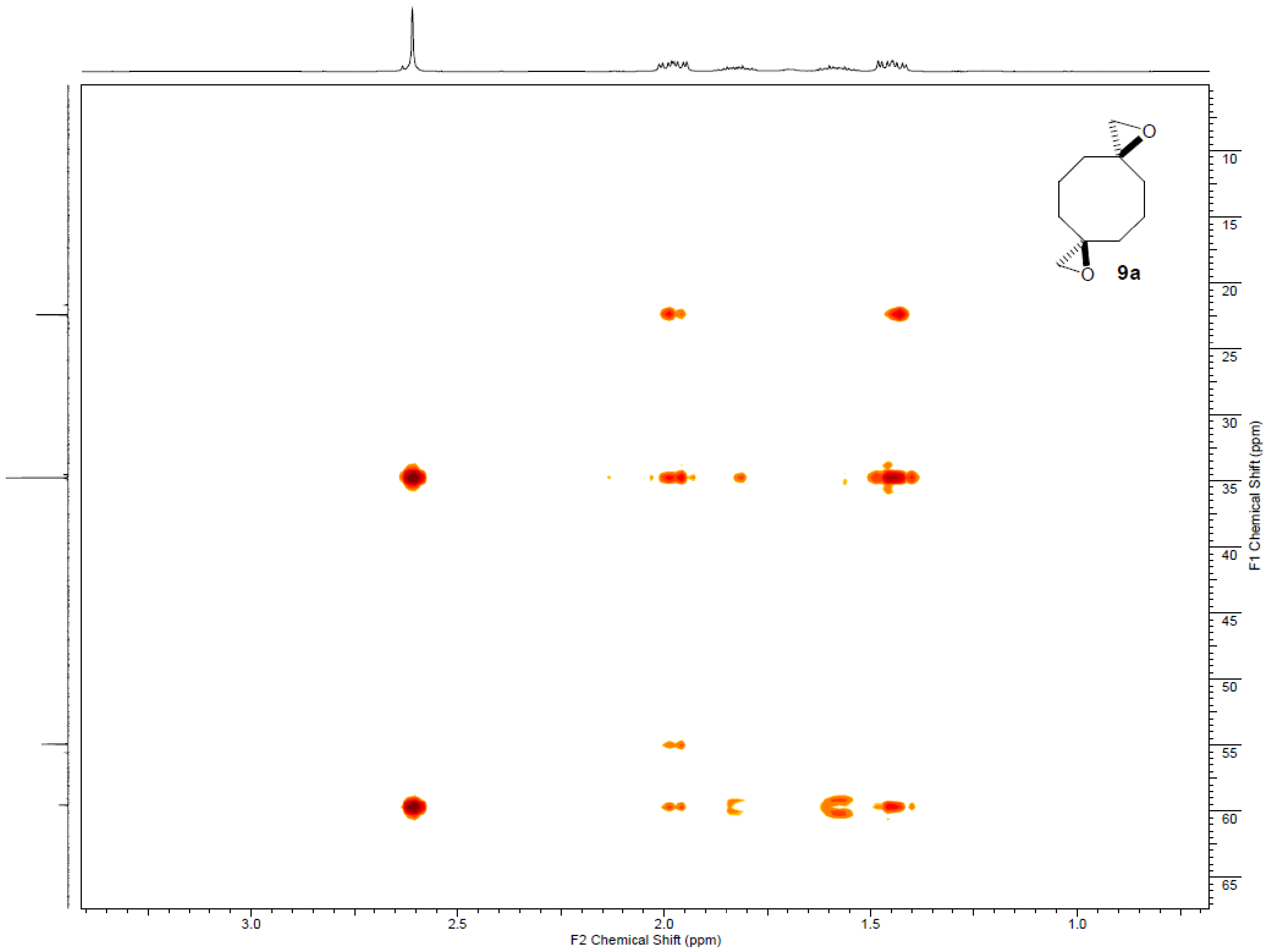

^{1}H NMR ($CDCl_3$) spectrum of compound **9b**

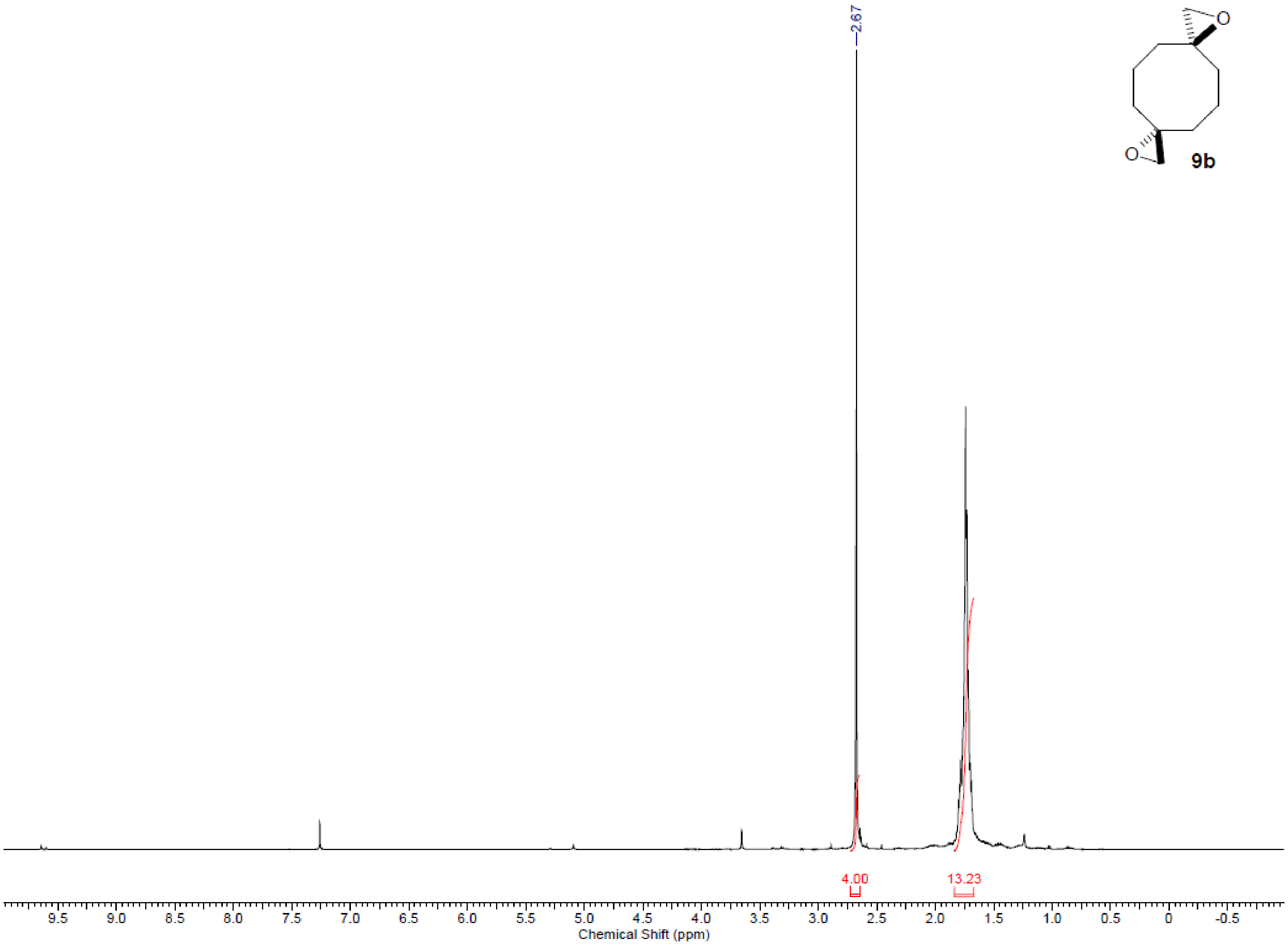

^{13}C NMR ($CDCl_3$) spectrum of compound **9b**

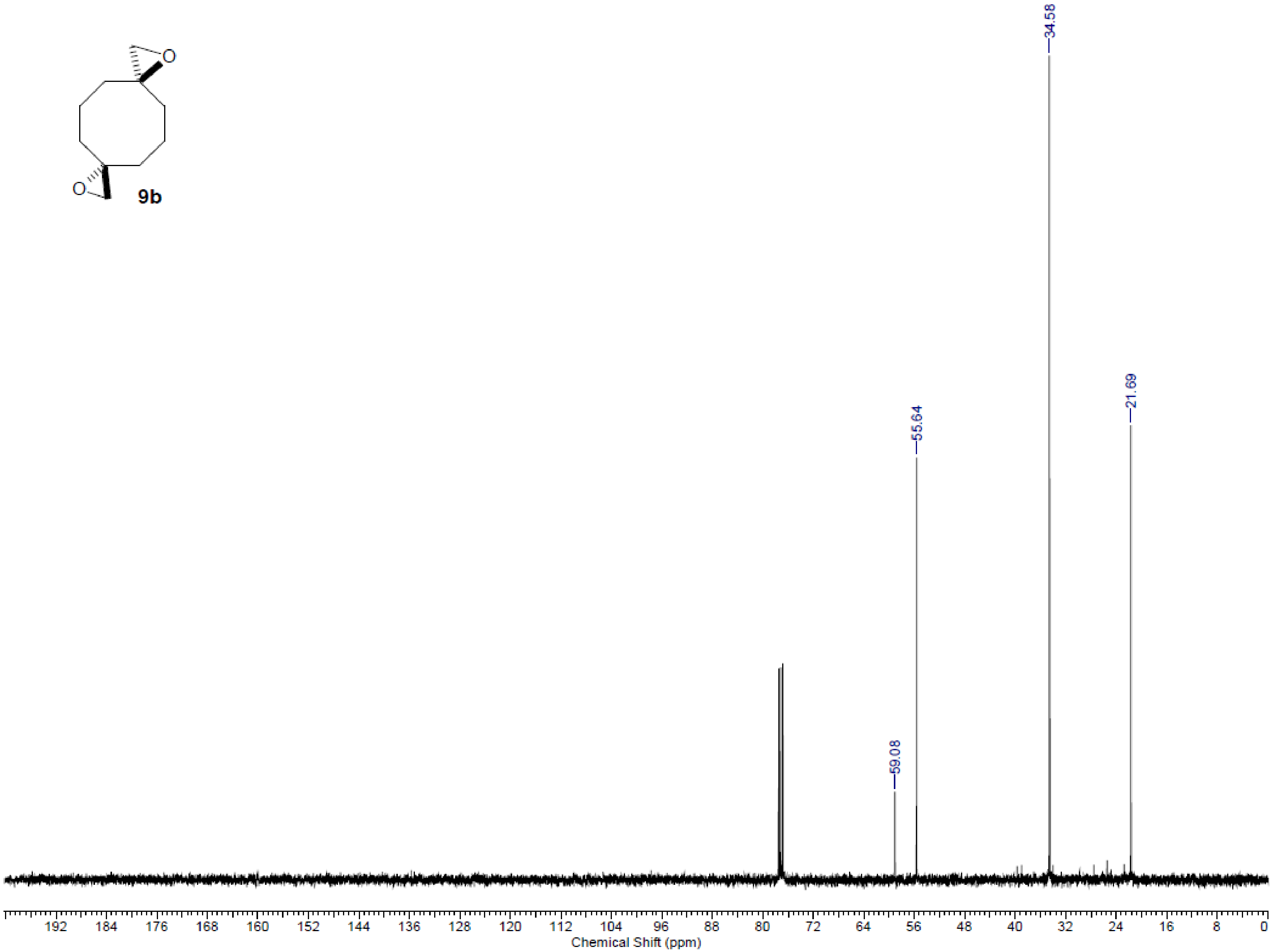

HSQC NMR ($CDCl_3$) spectrum of compound **9b**

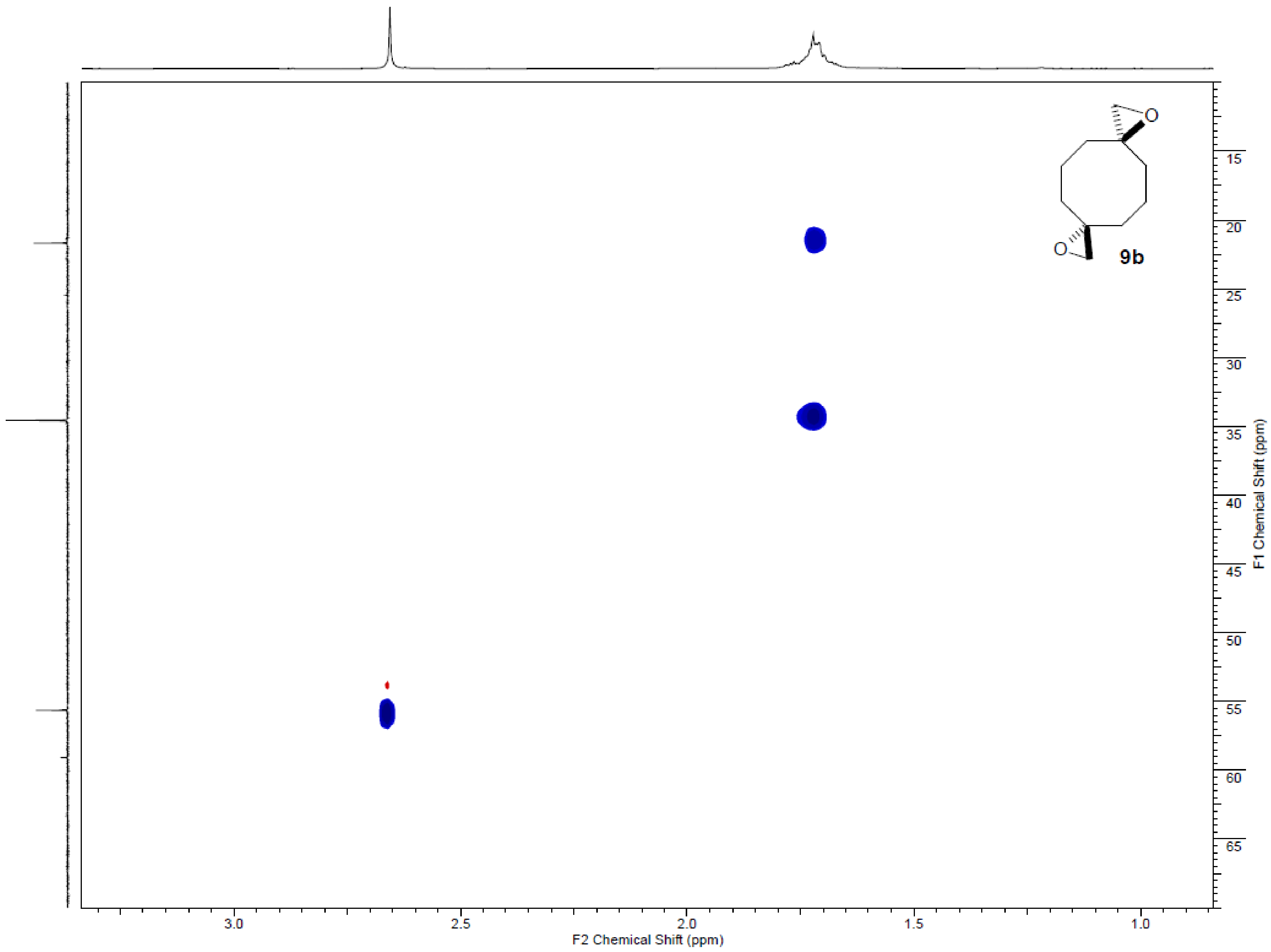

HMBC NMR ($CDCl_3$) spectrum of compound **9b**

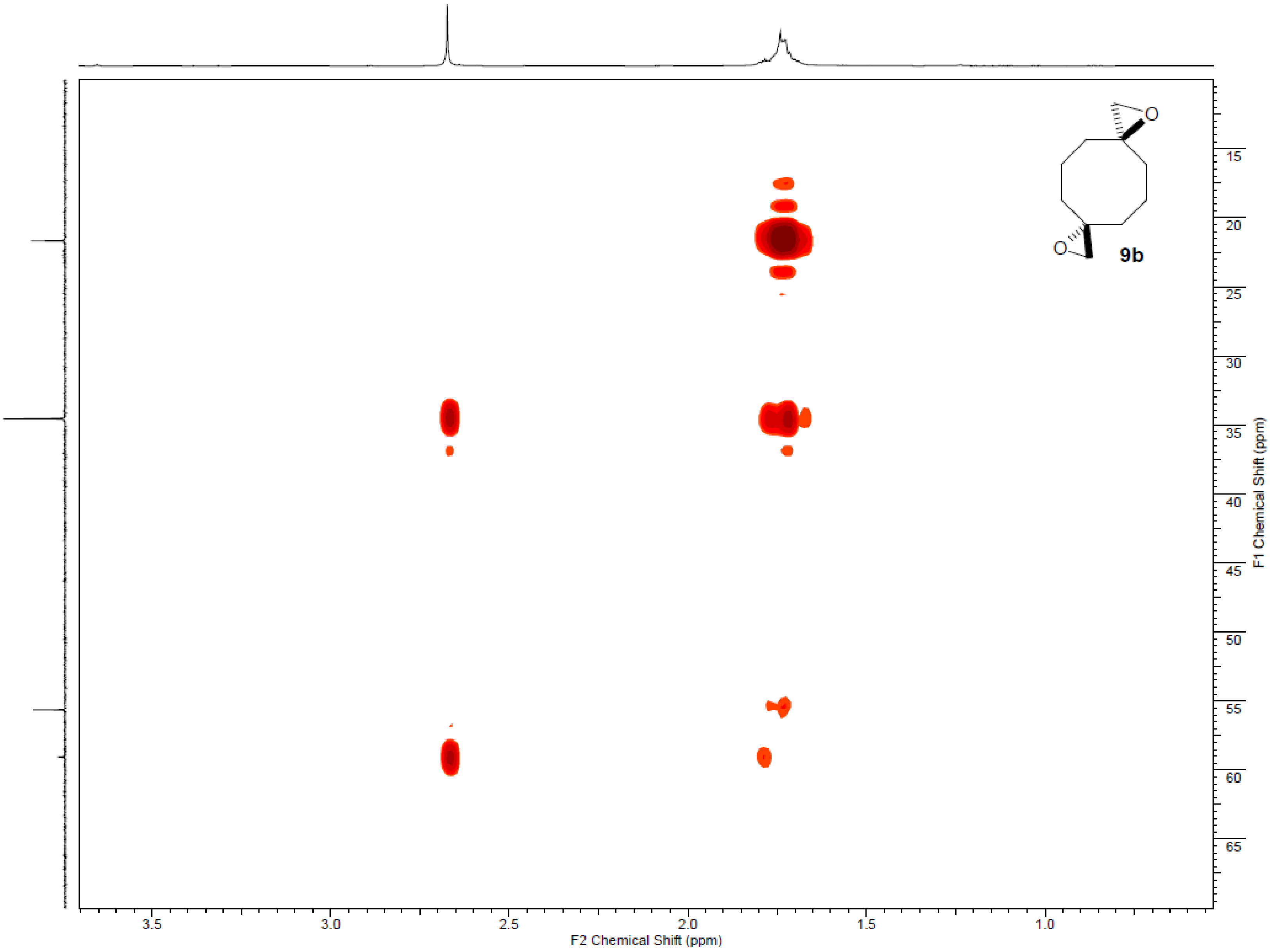

^{1}H NMR (CDCl$_3$) spectrum of compound **11**

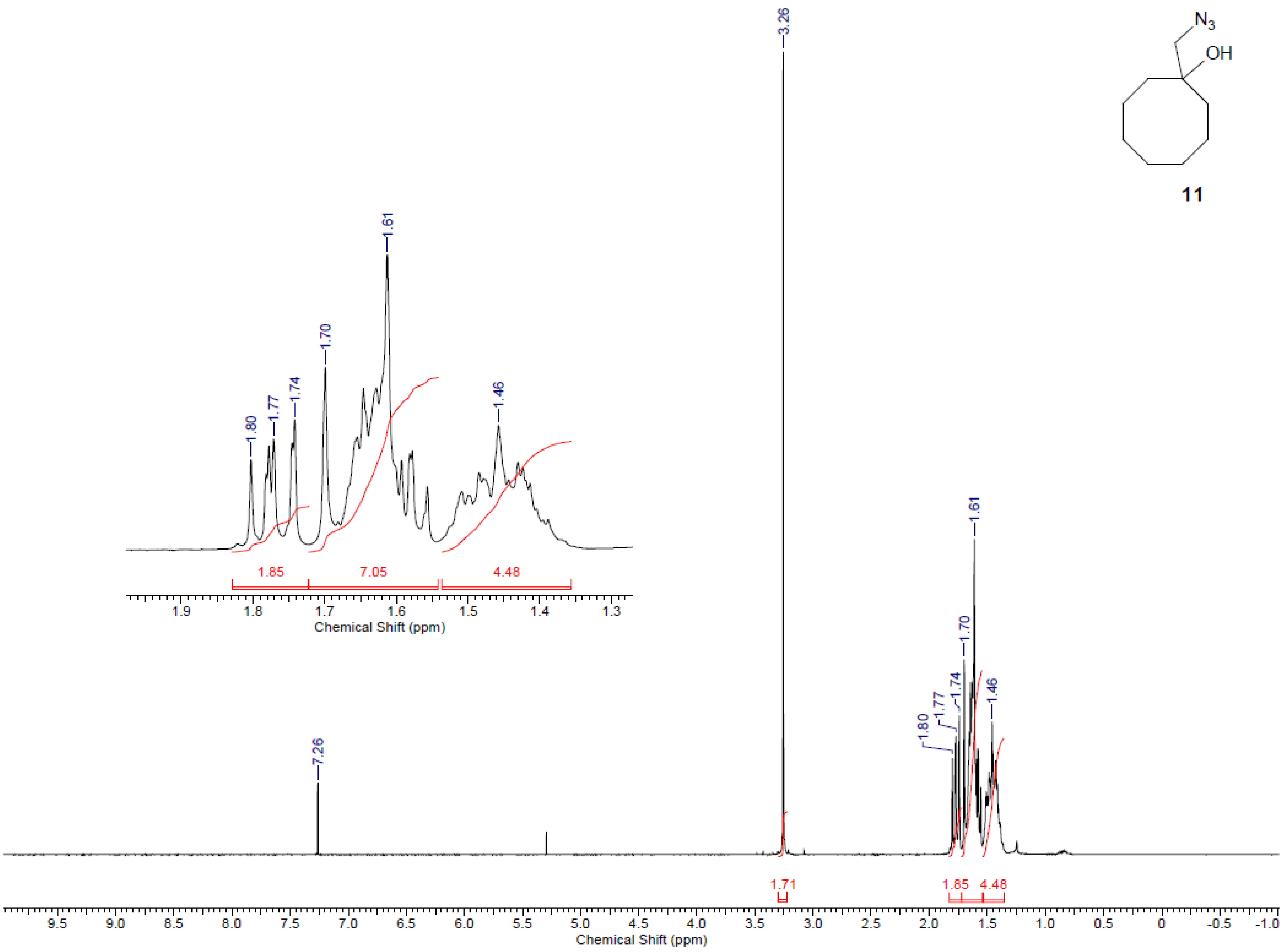

11

^{13}C NMR ($CDCl_3$) spectrum of compound **11**

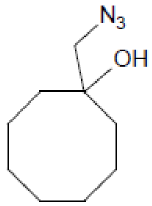

11

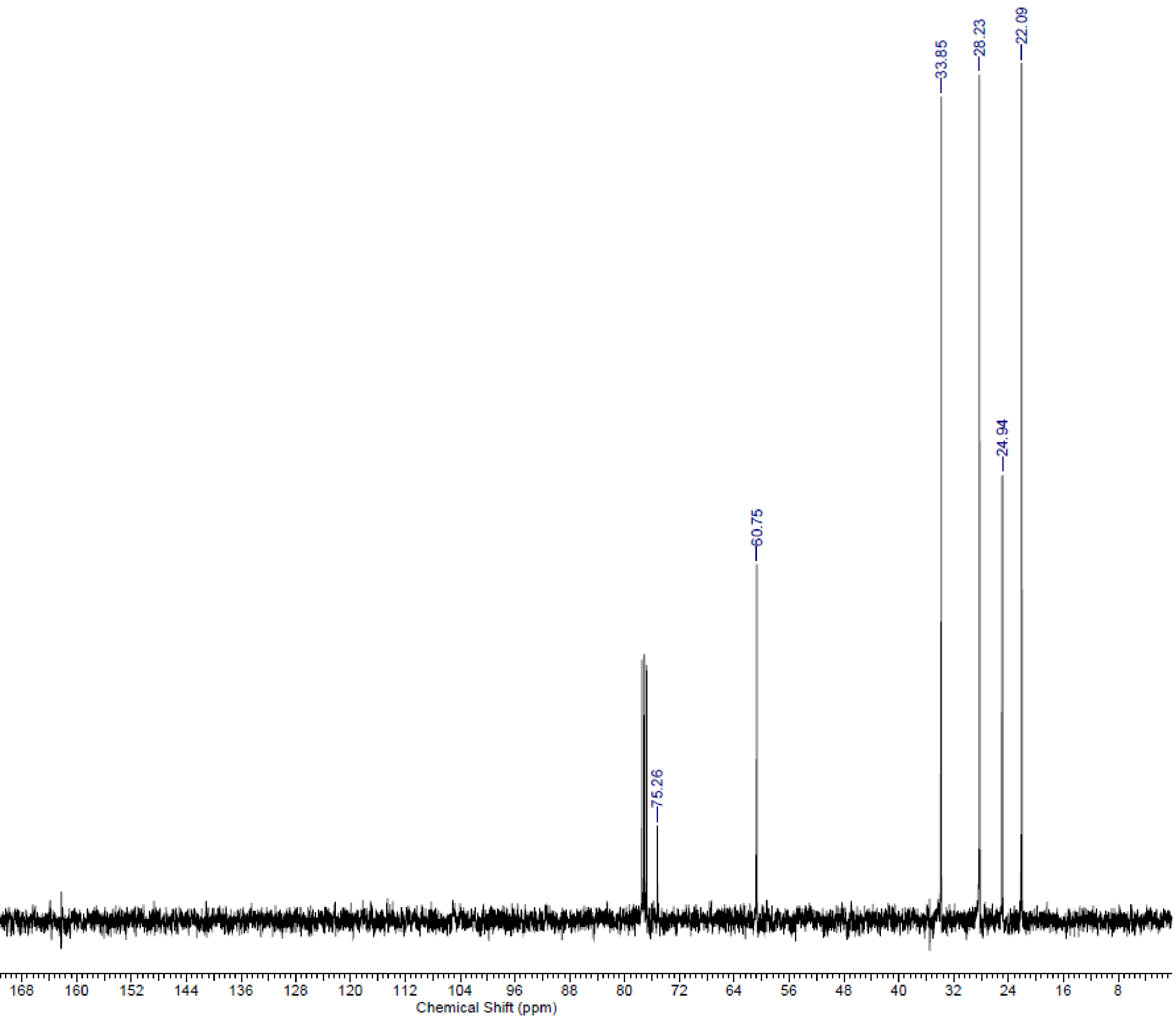

HSQC NMR ($CDCl_3$) spectrum of compound **11**

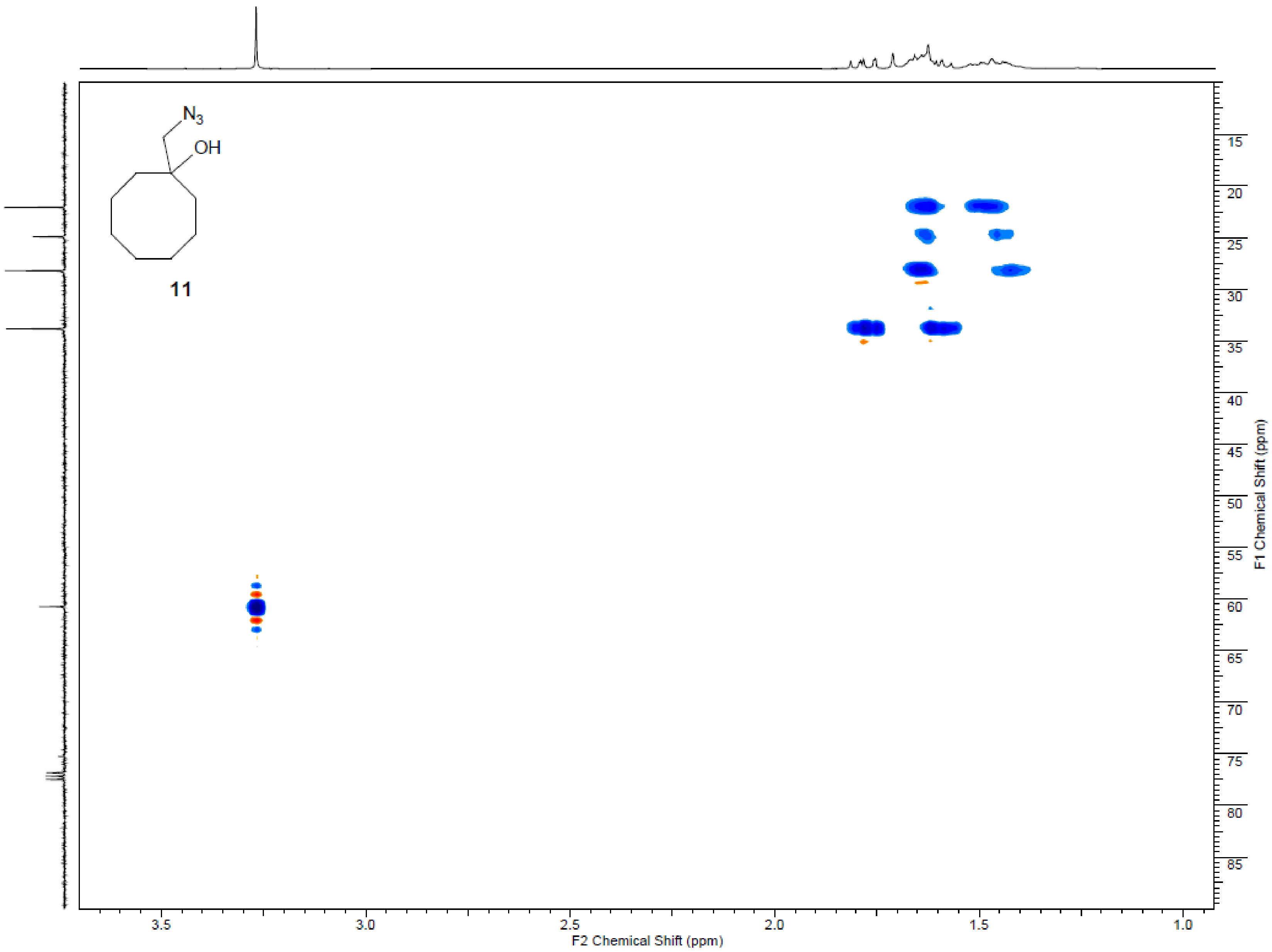

^{1}H NMR ($CDCl_3$) spectrum of compound **12**

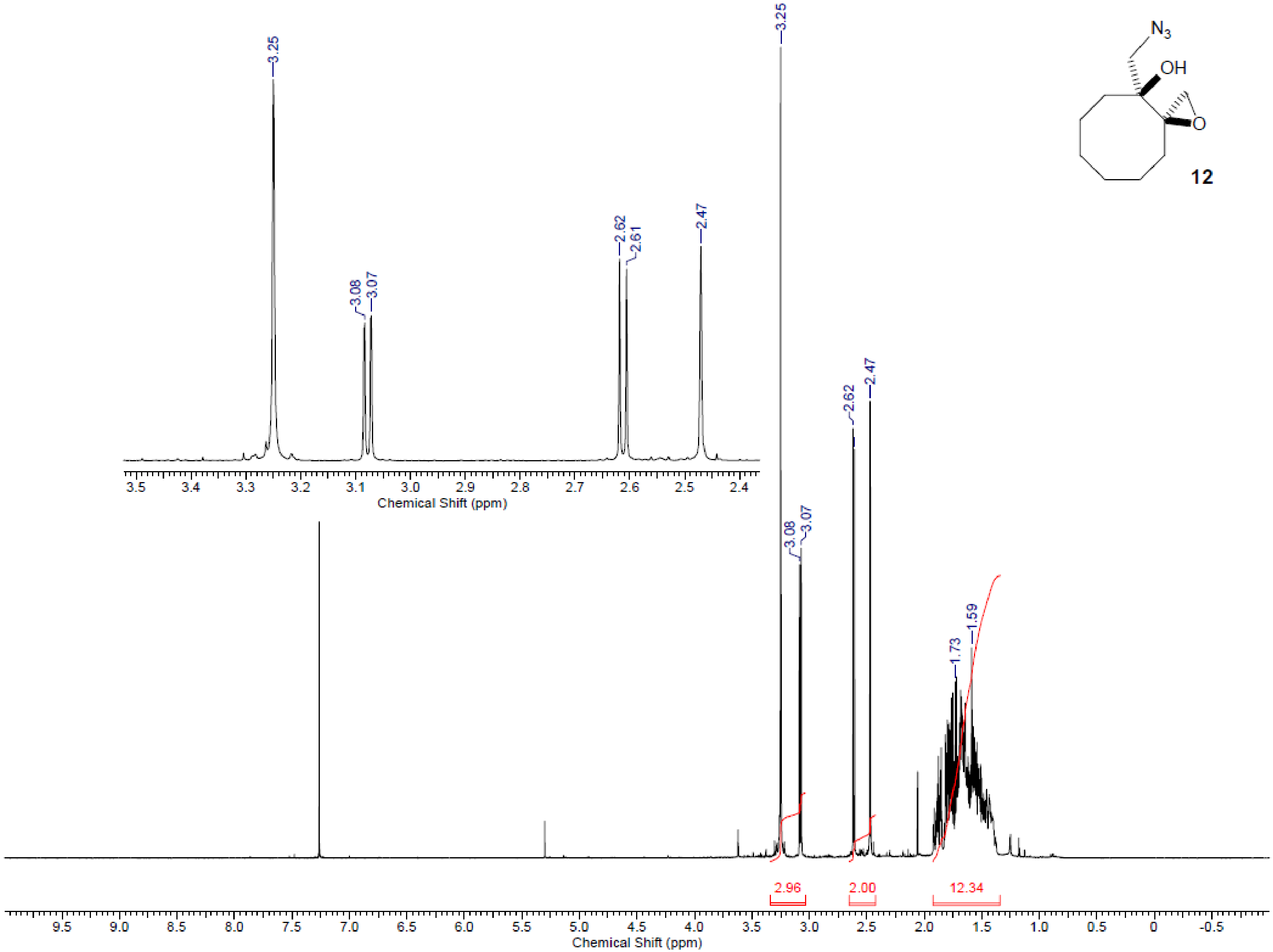

12

^{13}C NMR ($CDCl_3$) spectrum of compound **12**

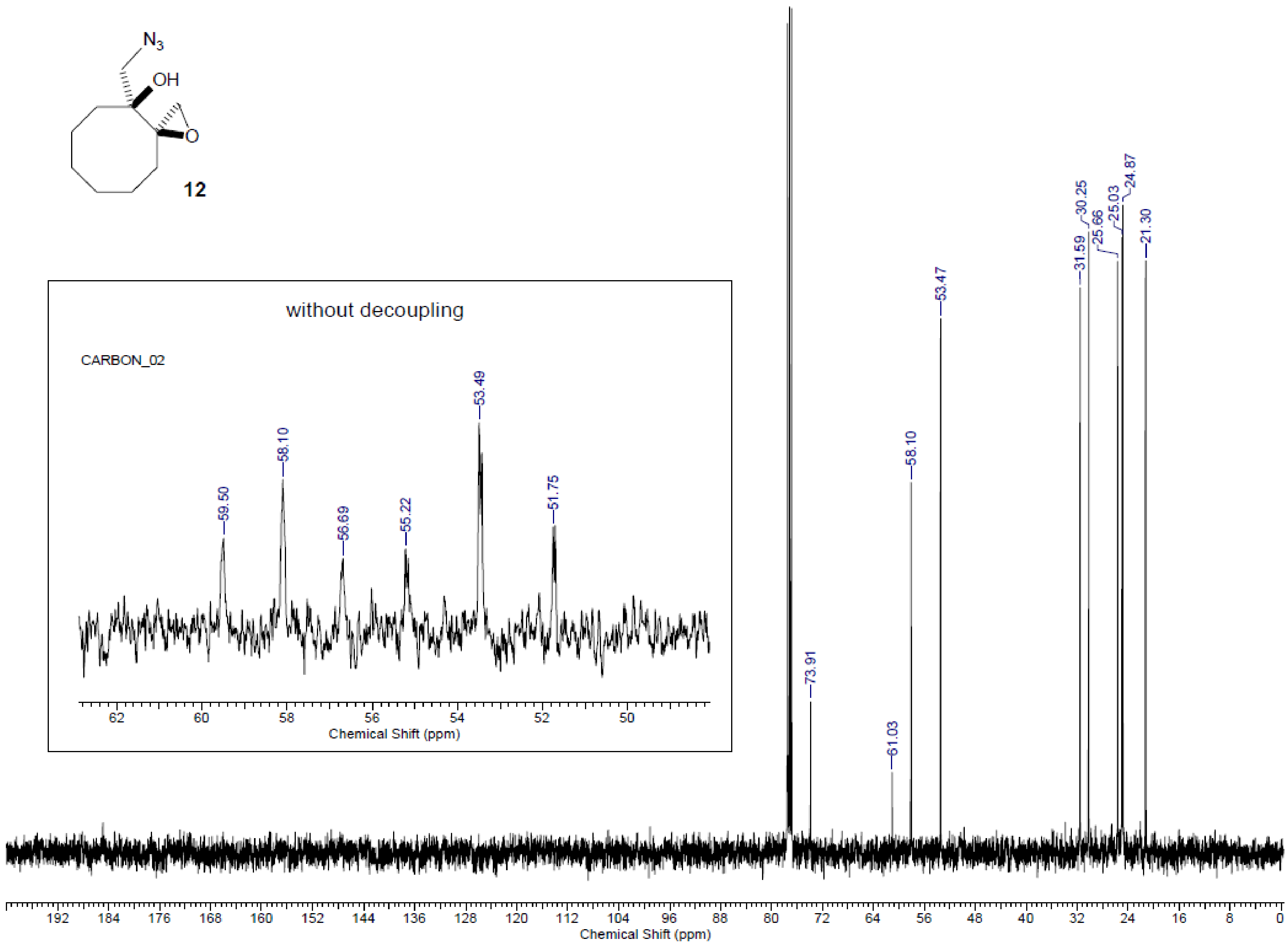

HSQC NMR ($CDCl_3$) spectrum of compound **12**

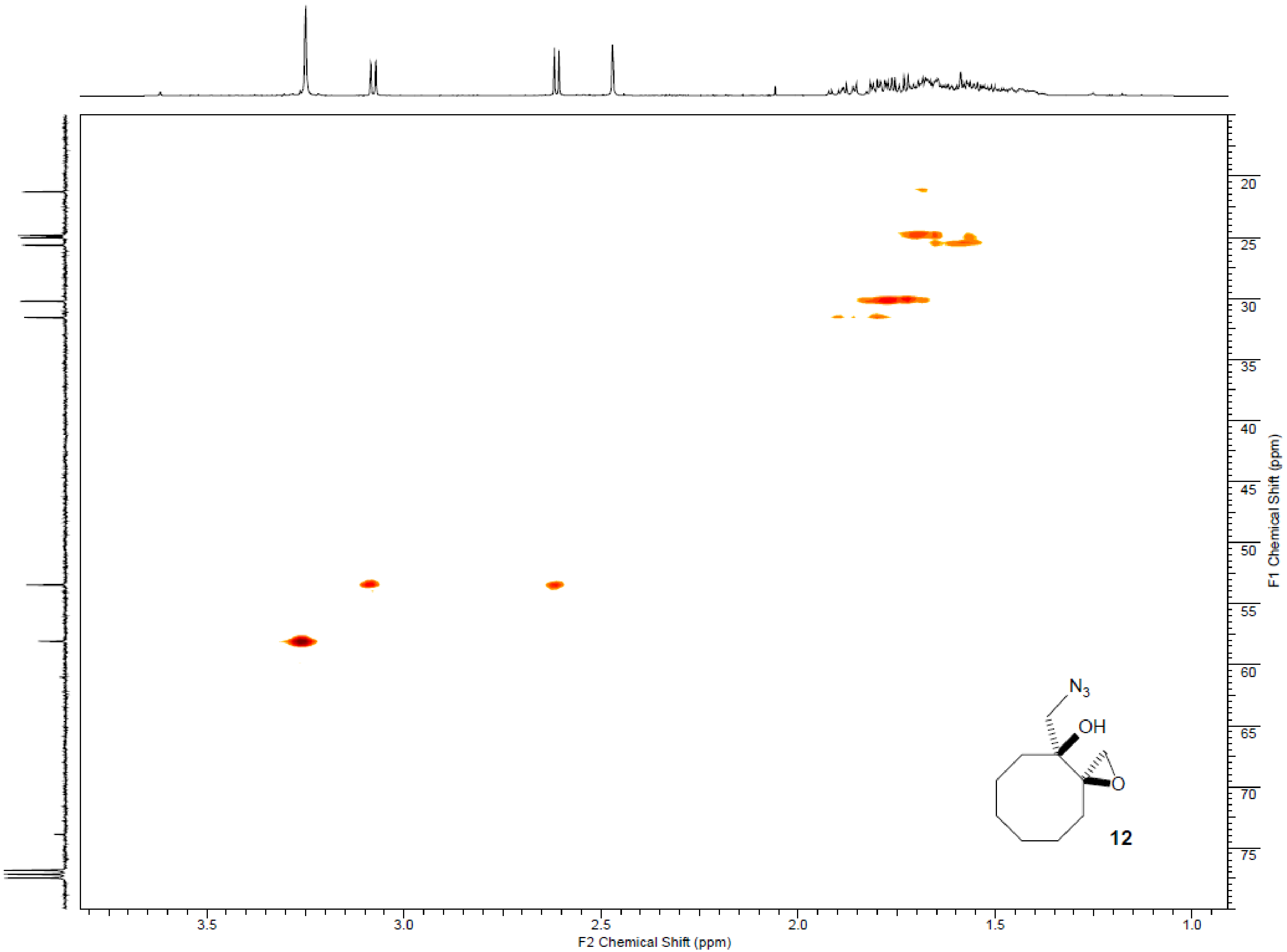

HMBC NMR ($CDCl_3$) spectrum of compound **12**

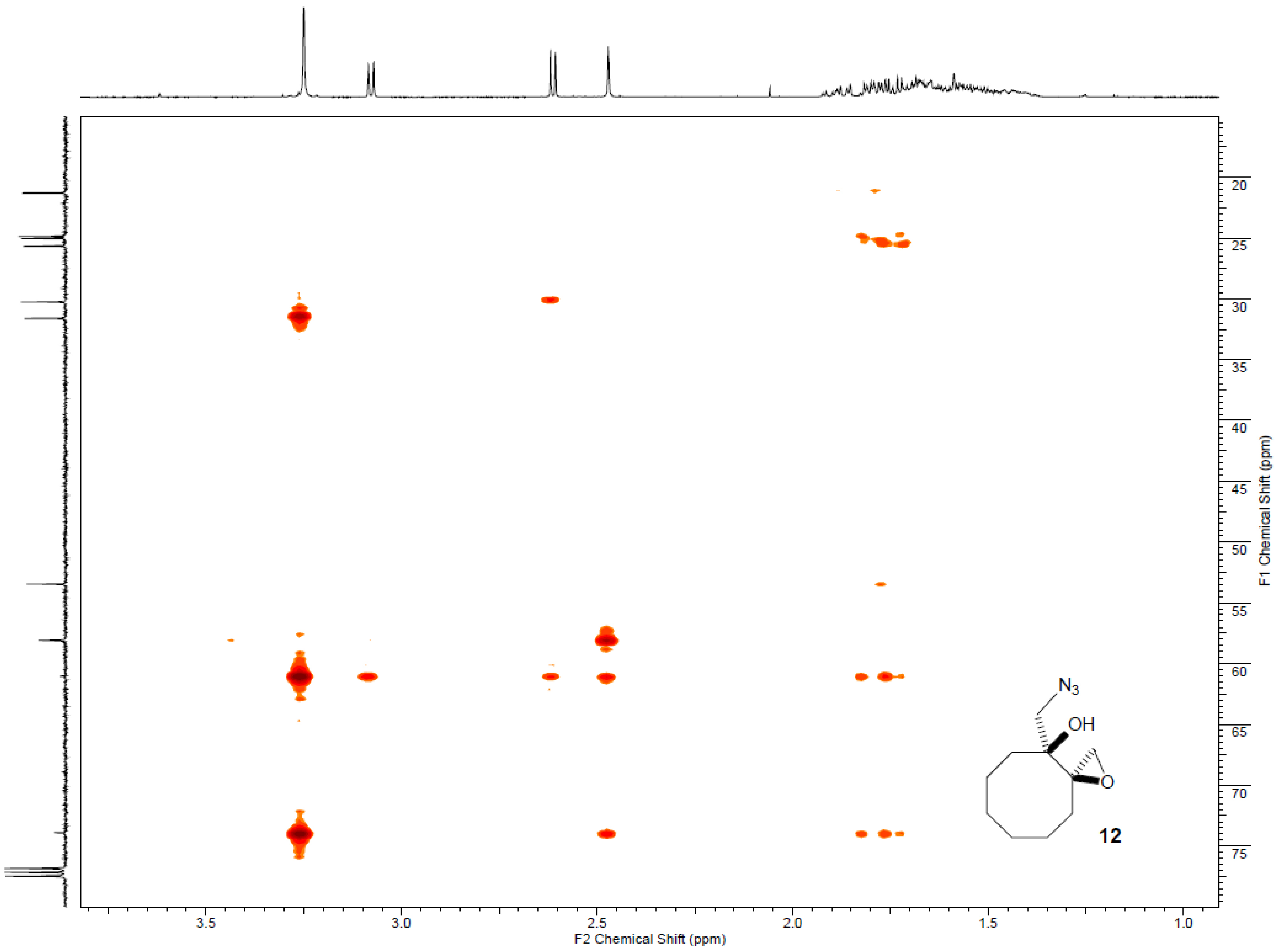

^{1}H NMR ($CDCl_3$) spectrum of compound **13**

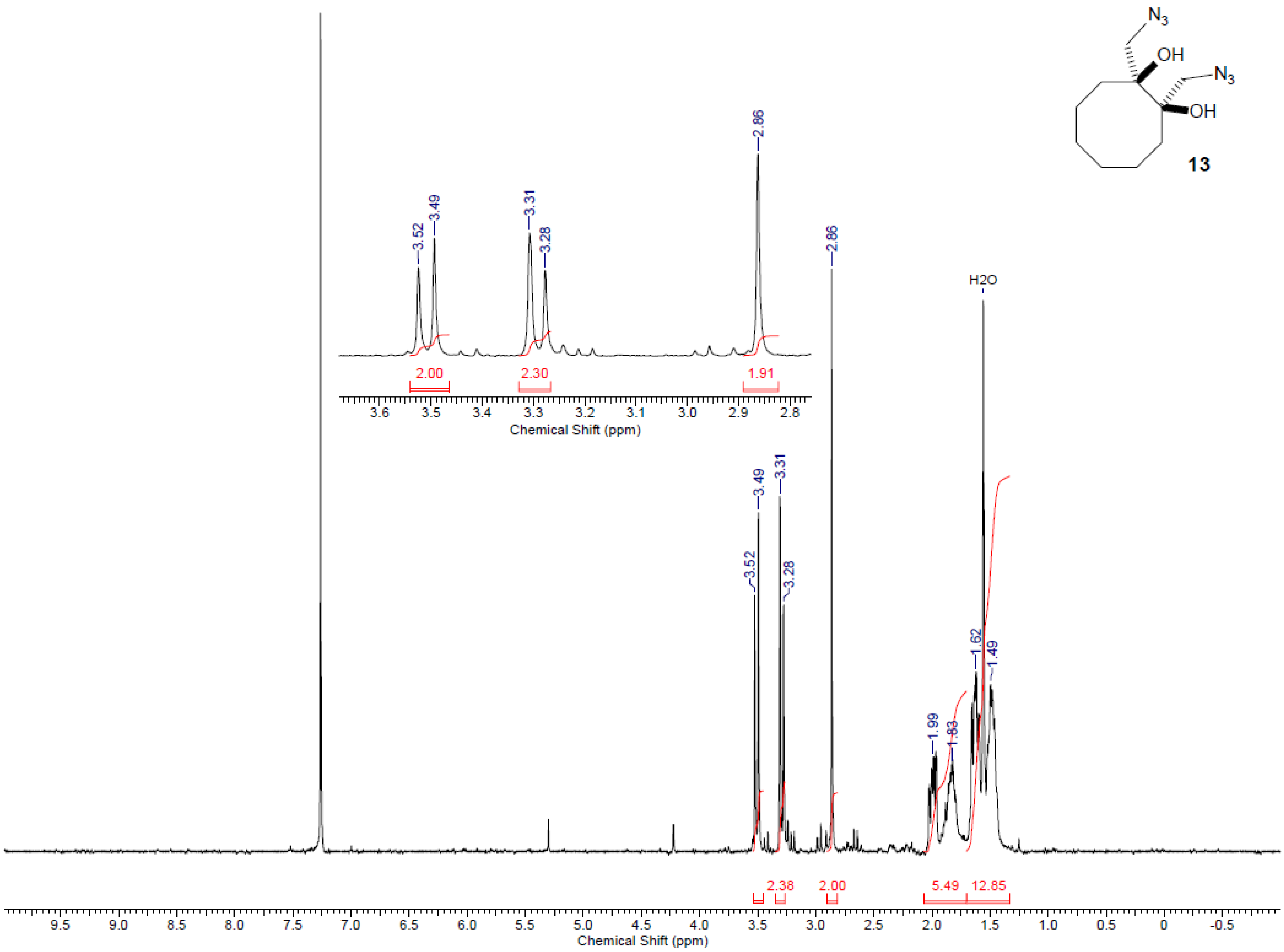

13

^{13}C NMR ($CDCl_3$) spectrum of compound **13**

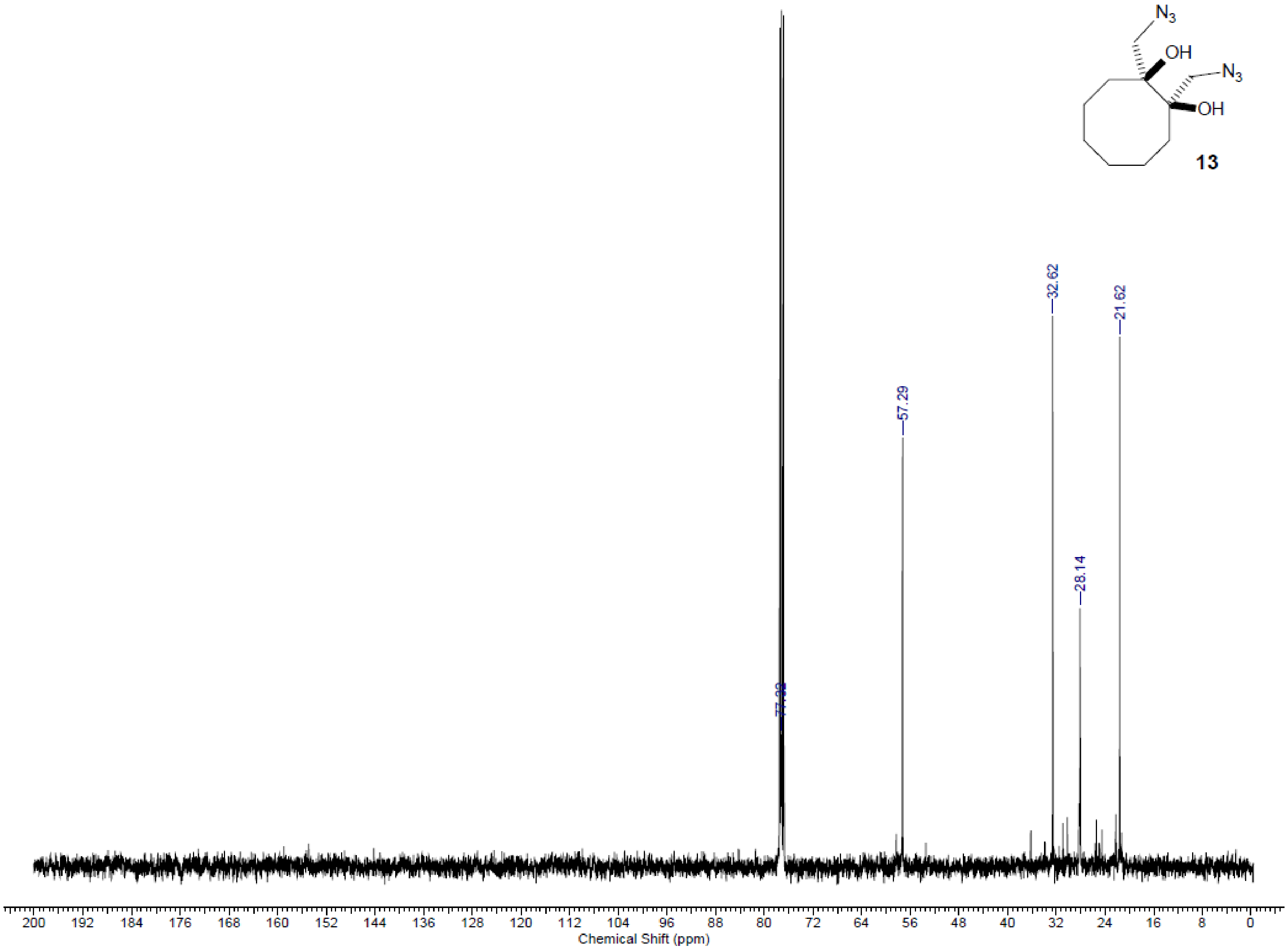

HSQC NMR ($CDCl_3$) spectrum of compound **13**

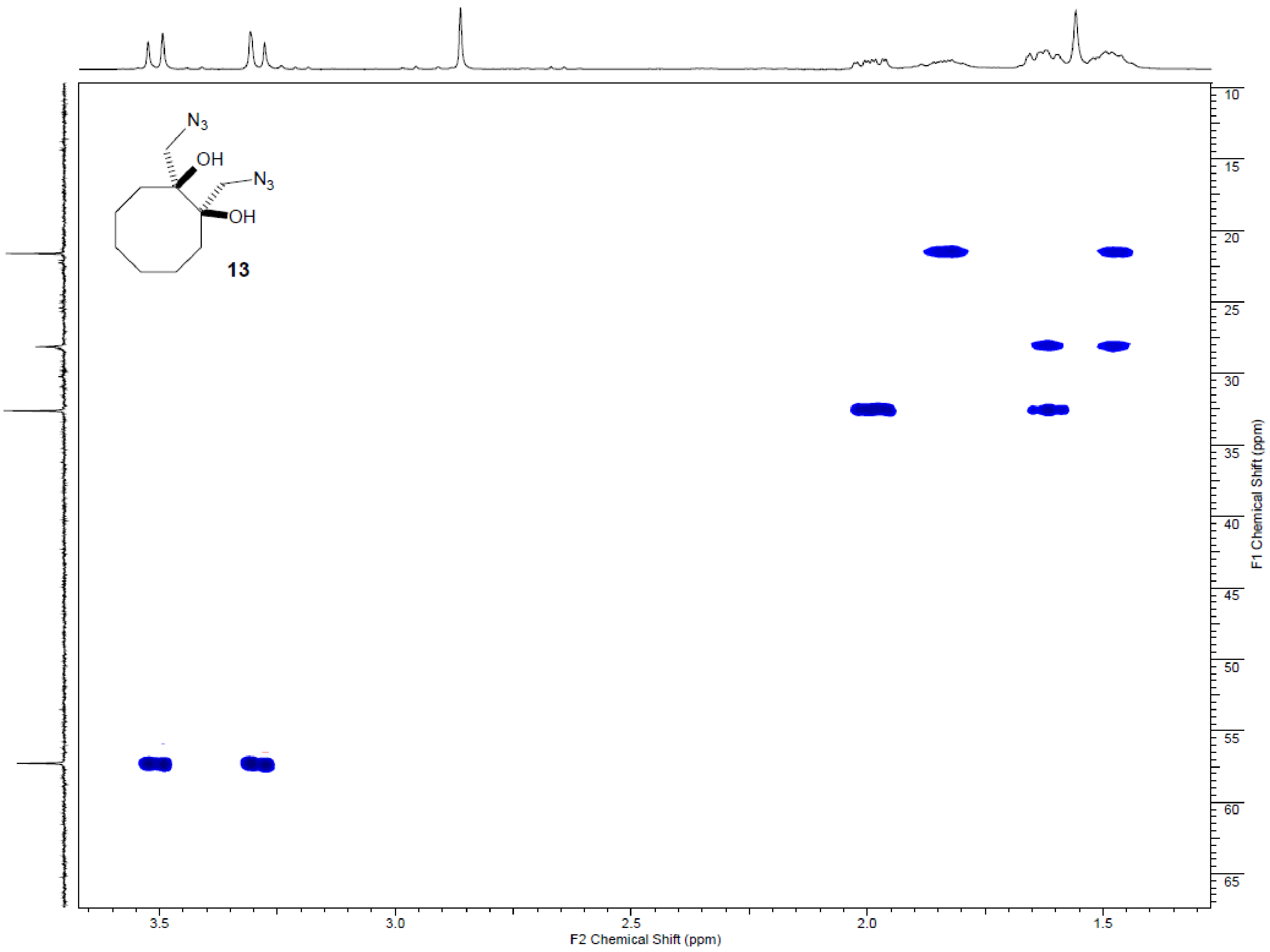

HSQC NMR ($CDCl_3$) spectrum of compound **13**

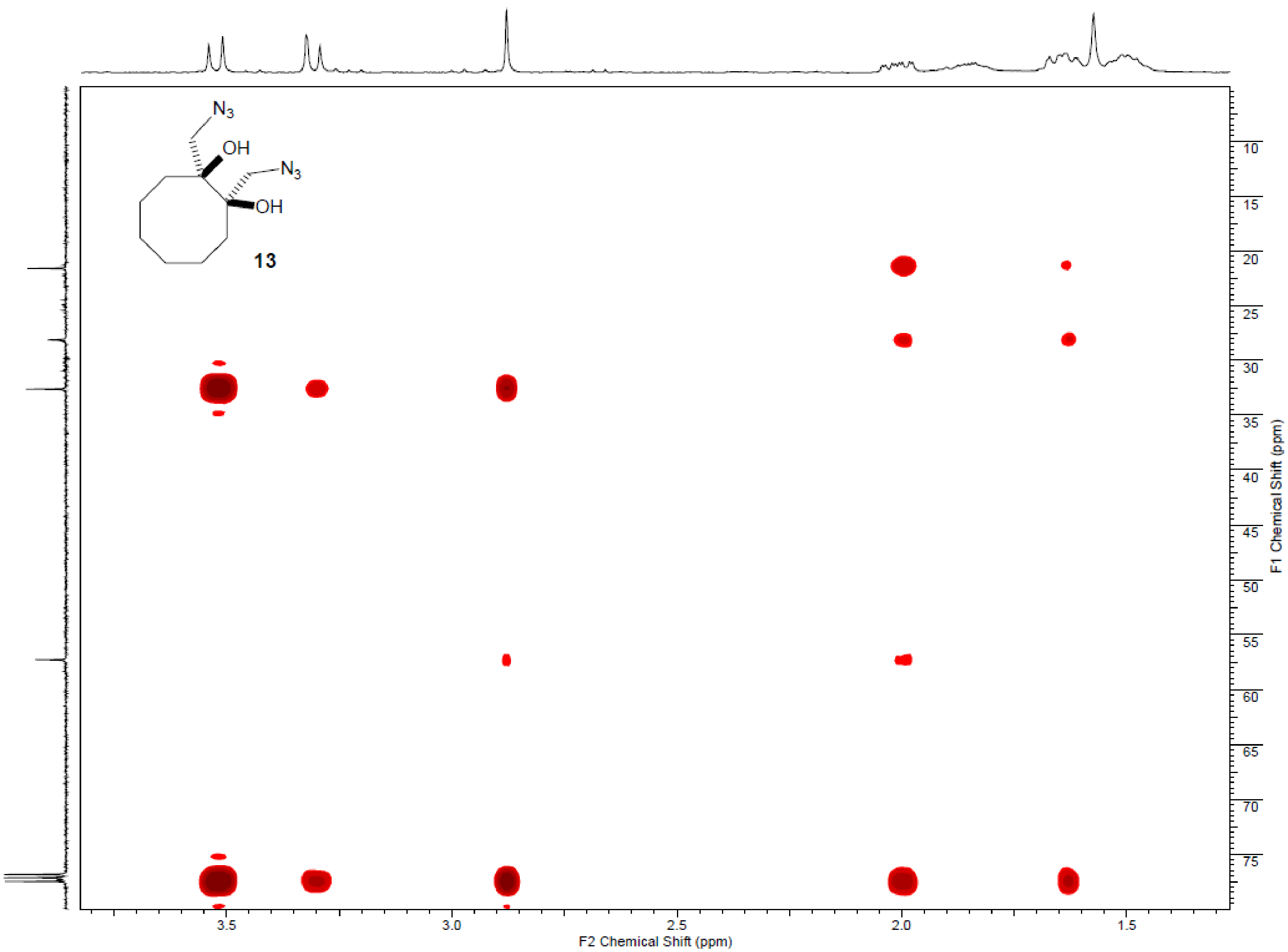

^1H NMR ($CDCl_3$) spectrum of compound **14**

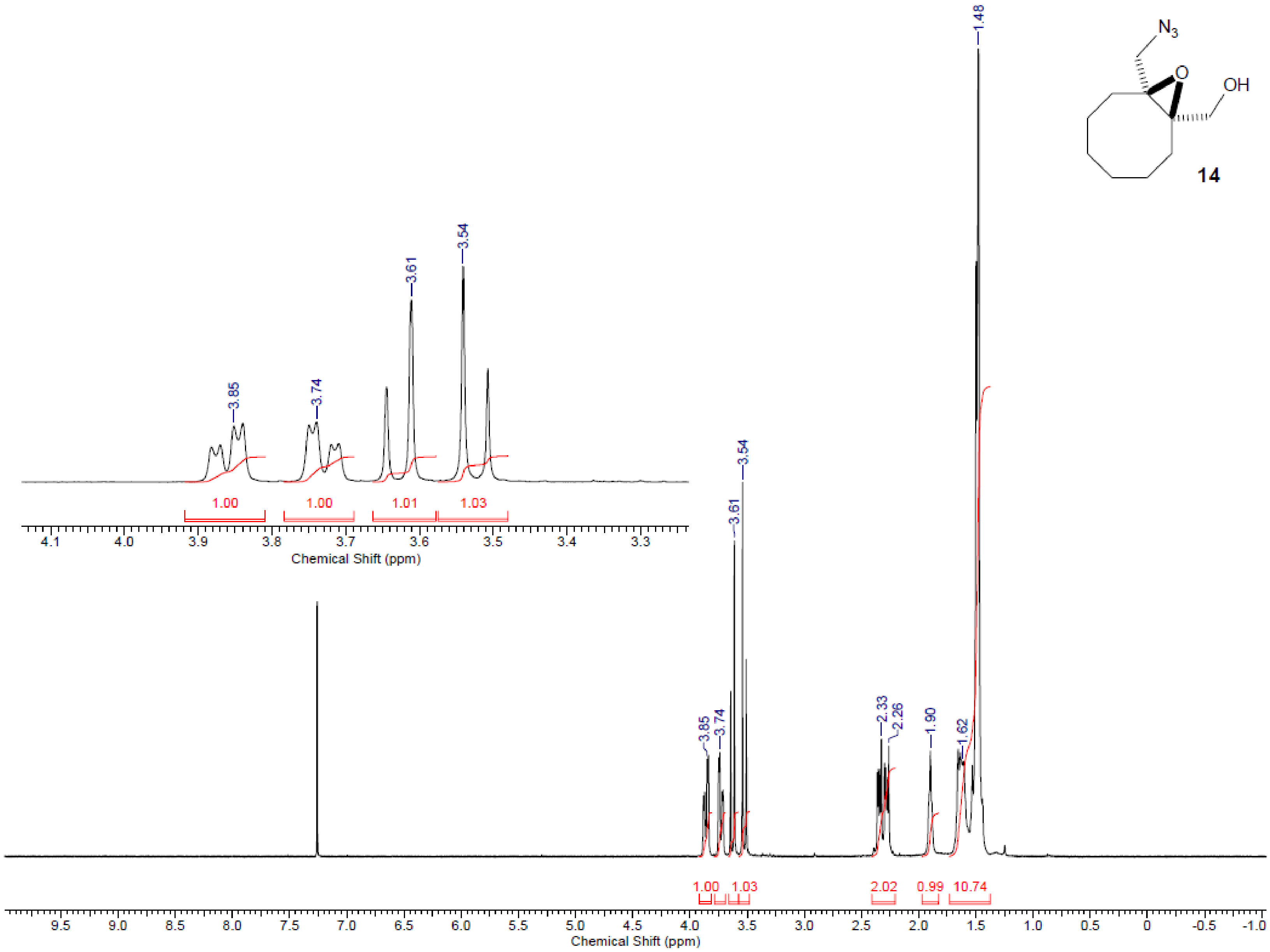

^{13}C NMR ($CDCl_3$) spectrum of compound **14**

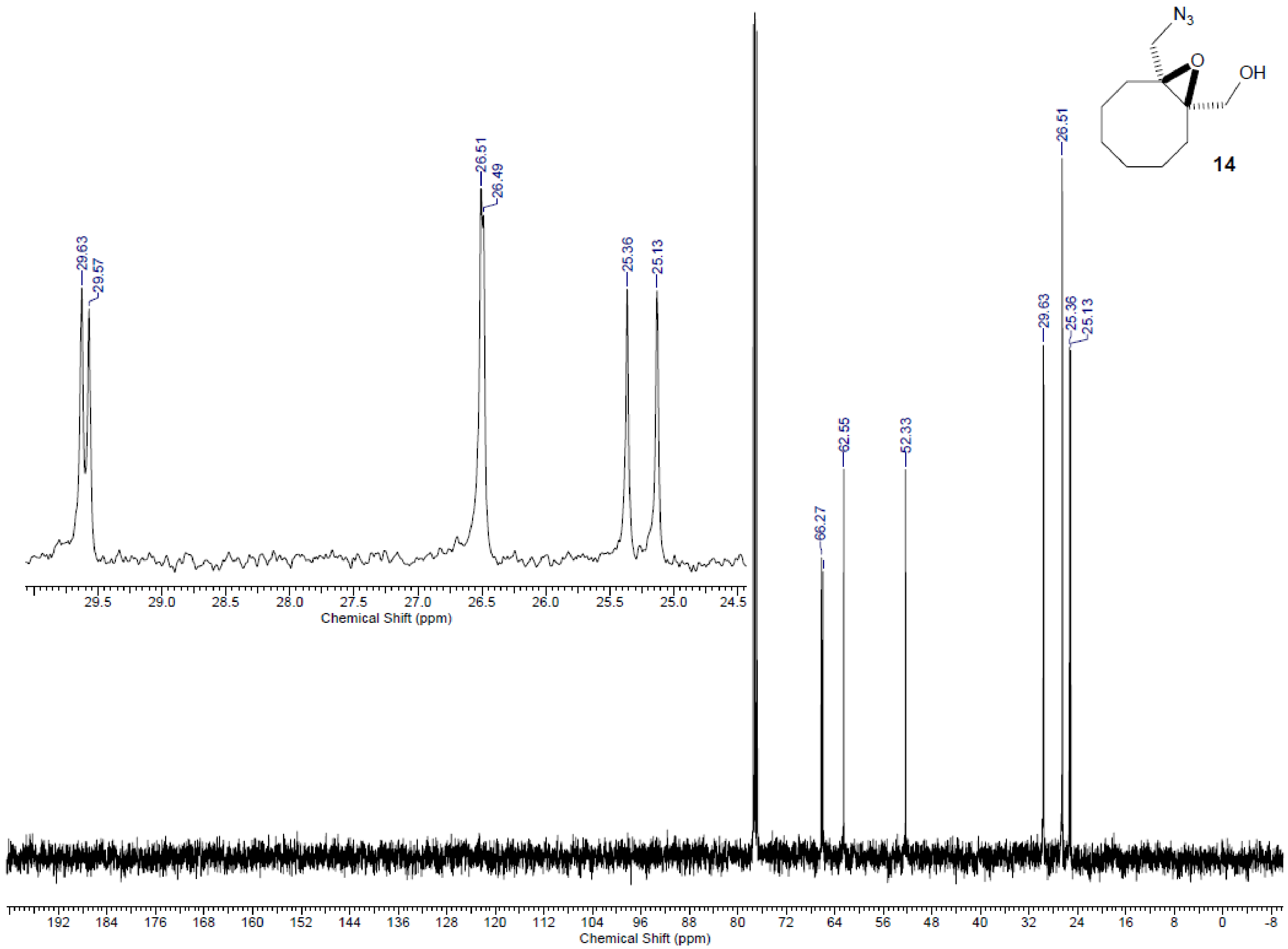

COSY H-H NMR ($CDCl_3$) spectrum of compound **14**

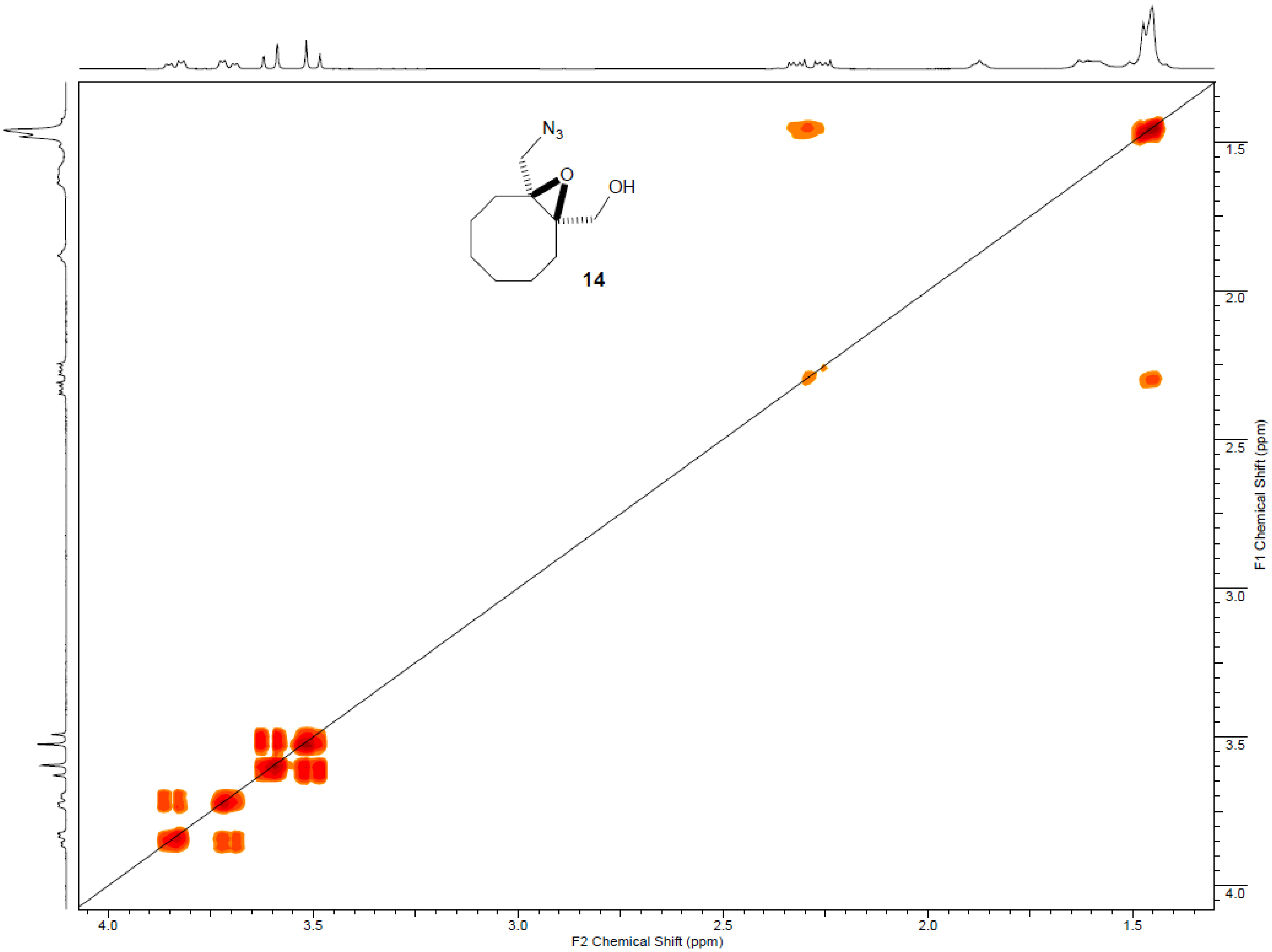

HSQC NMR ($CDCl_3$) spectrum of compound **14**

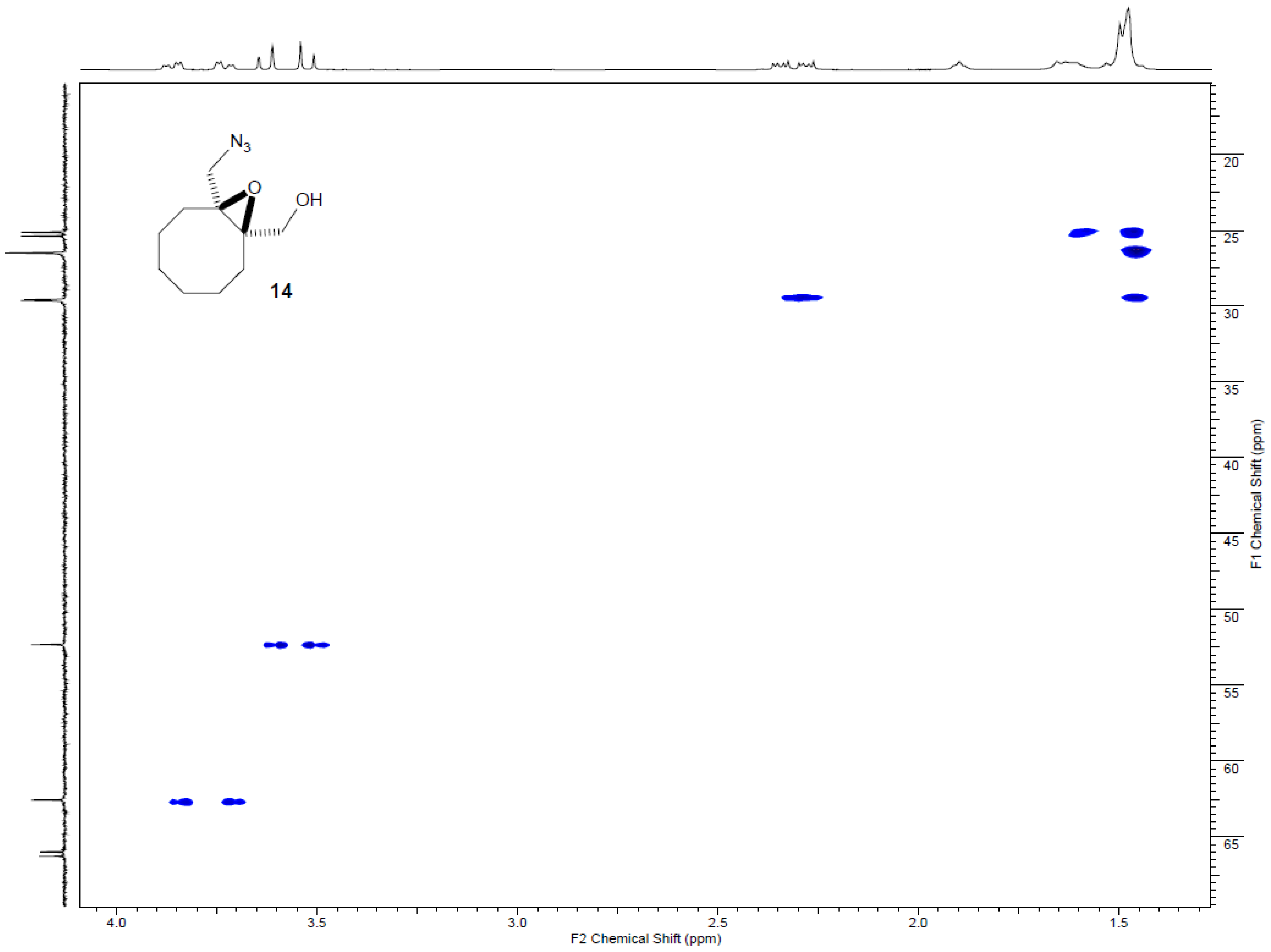

HMBC NMR ($CDCl_3$) spectrum of compound **14**

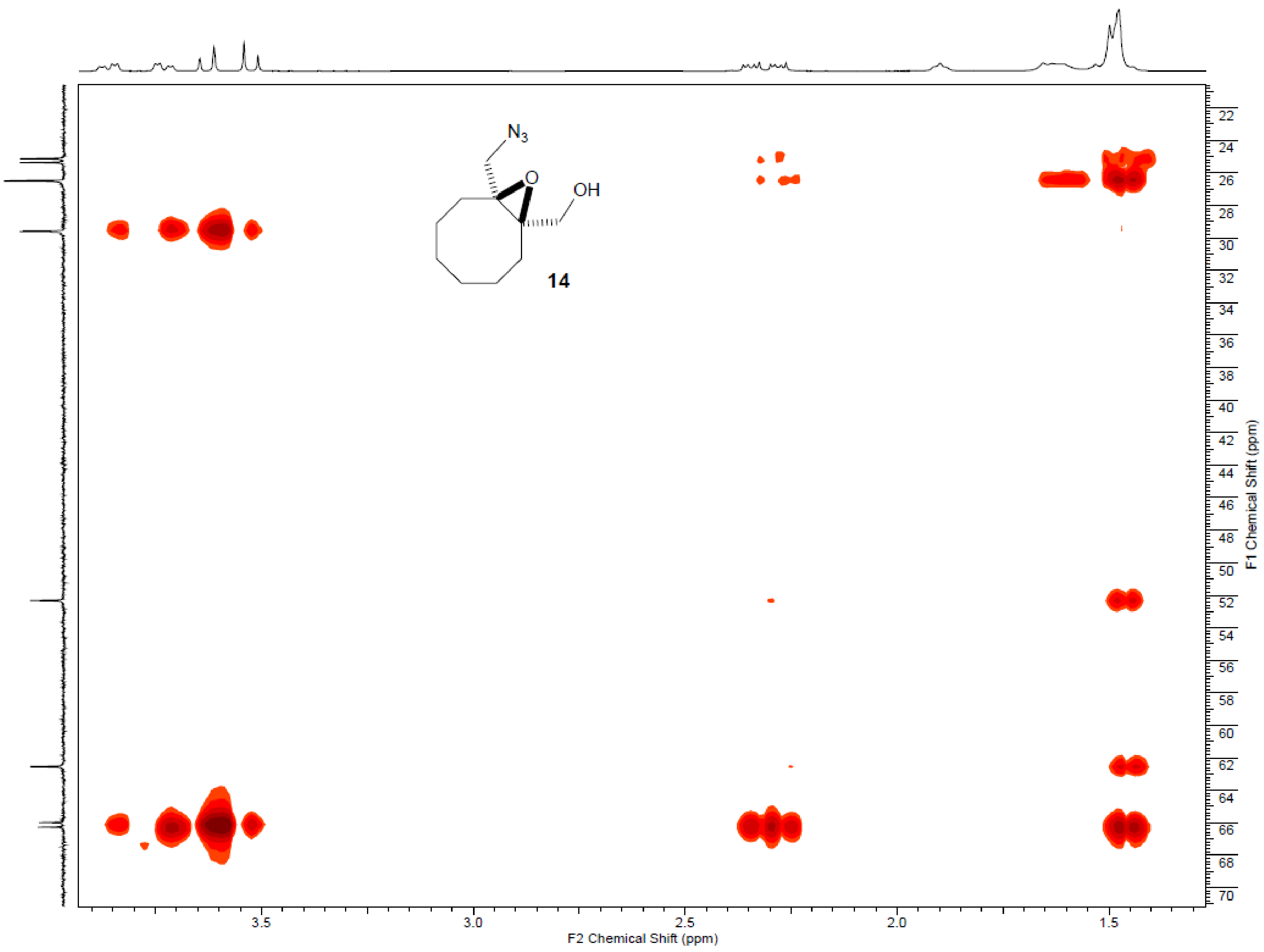

^1H NMR ($CDCl_3$) spectrum of compound **15**

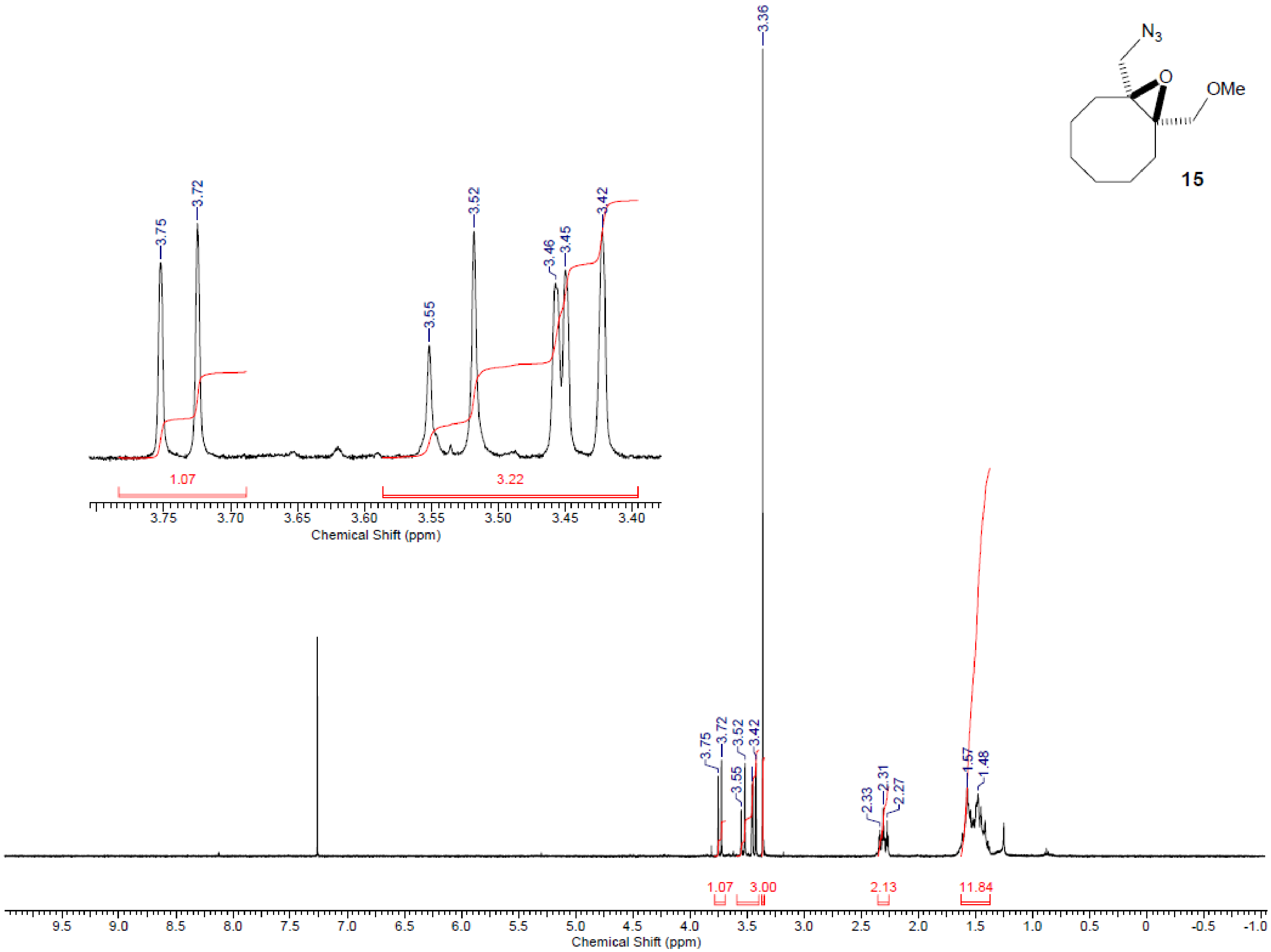

^{13}C NMR ($CDCl_3$) spectrum of compound **15**

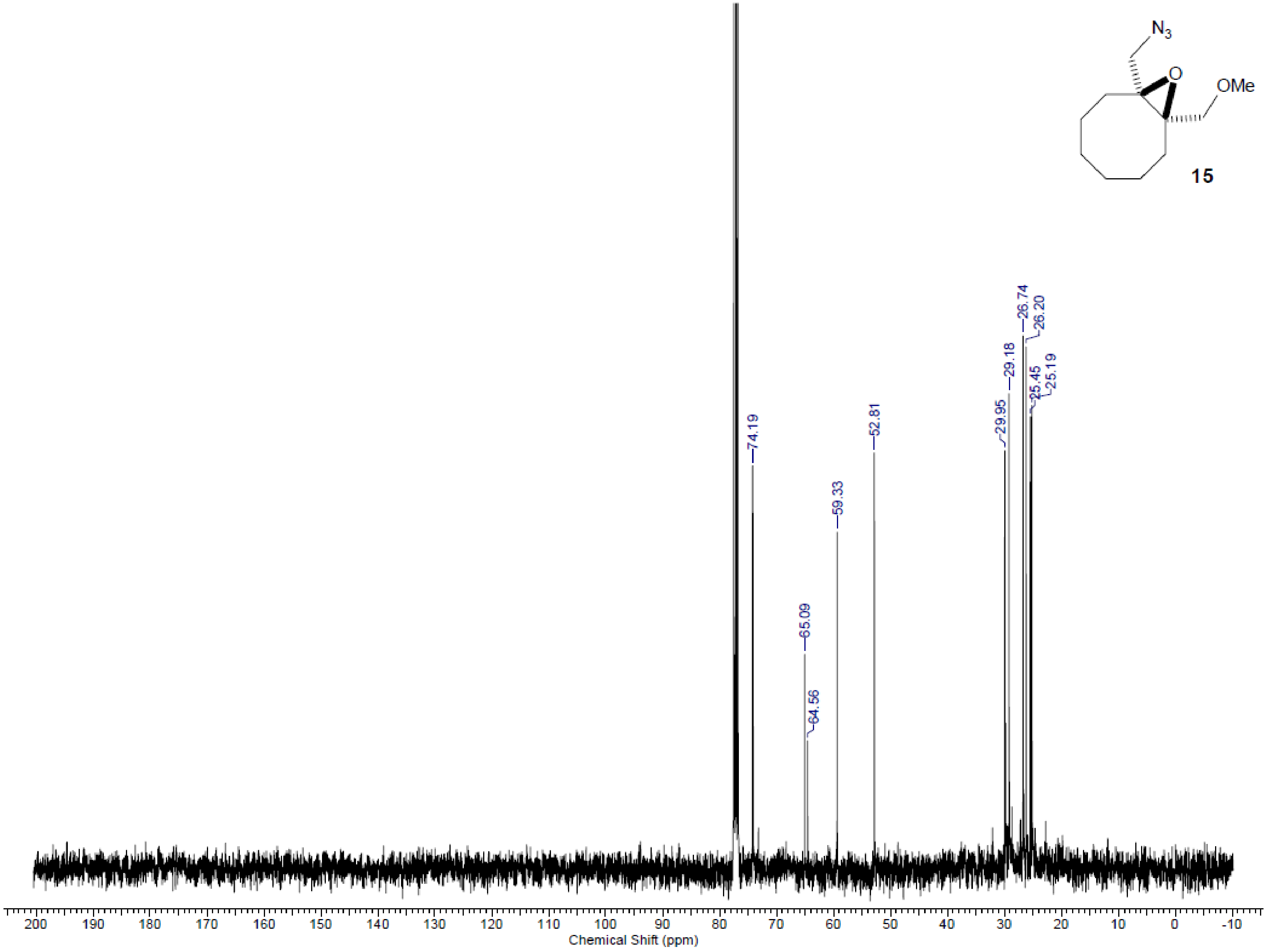

HSQC NMR ($CDCl_3$) spectrum of compound **15**

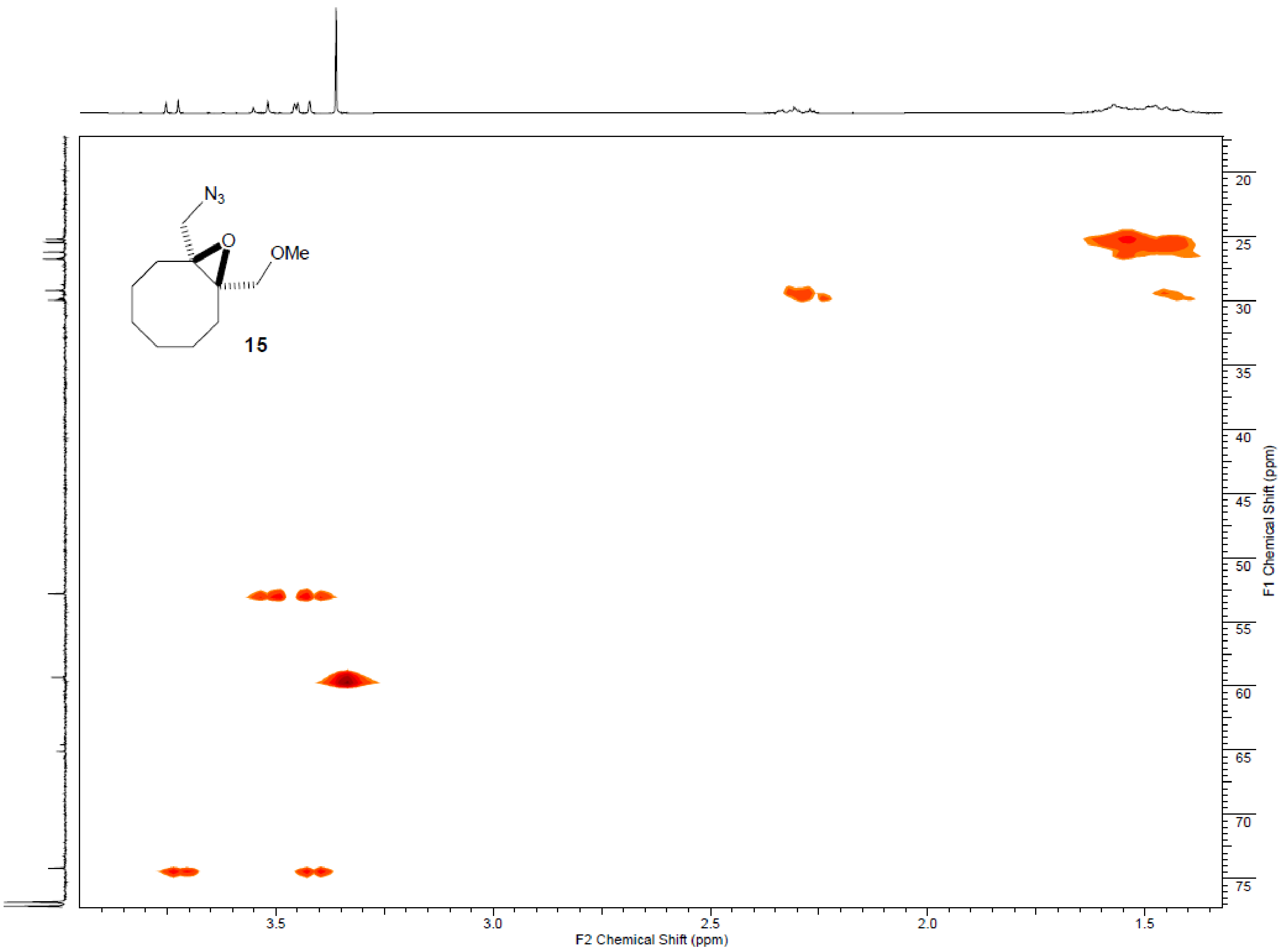

^1H NMR ($CDCl_3$) spectrum of compound **16**

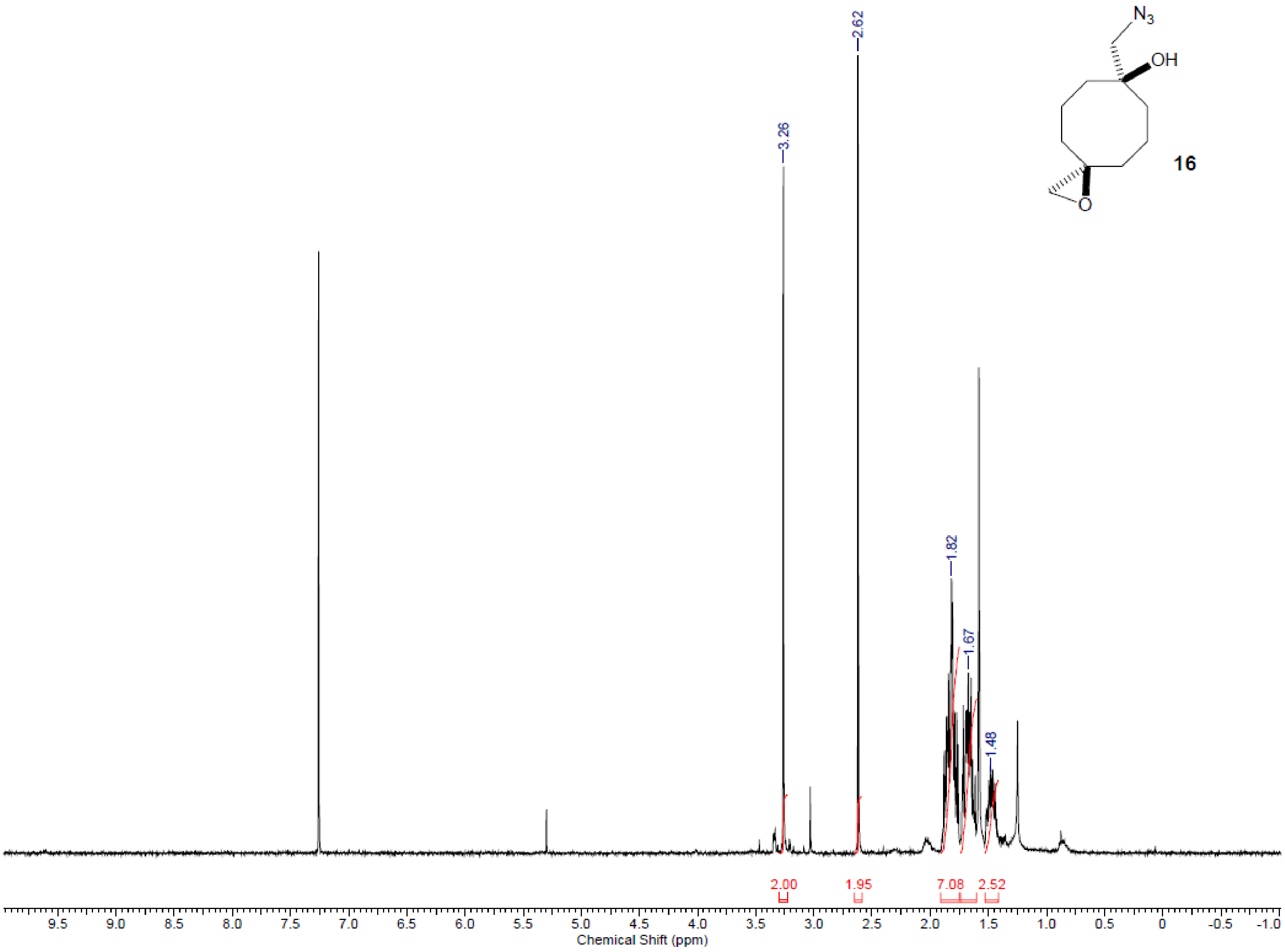

16

^{13}C NMR ($CDCl_3$) spectrum of compound **16**

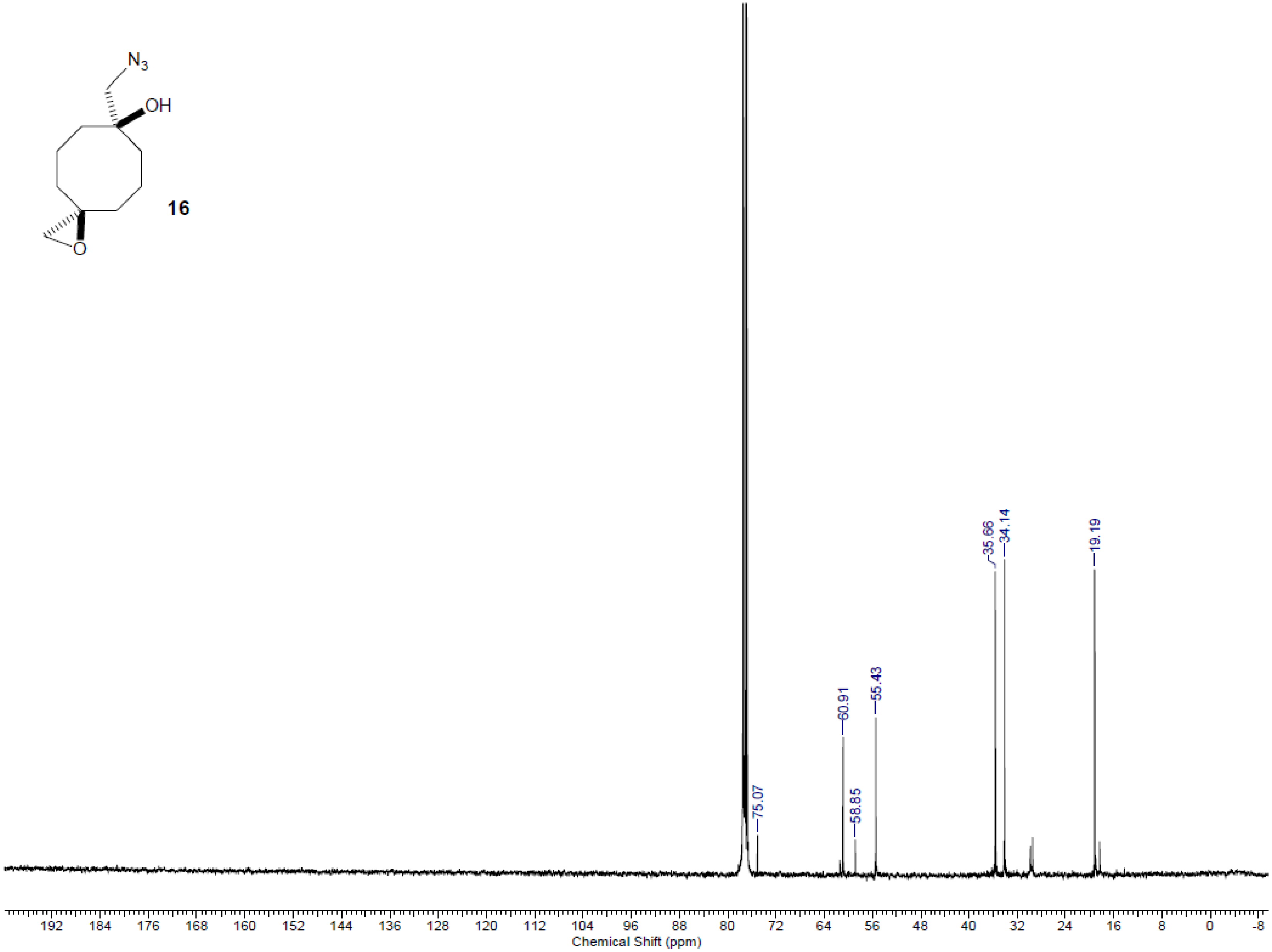

^{1}H NMR ($CDCl_3$) spectrum of compound **17**

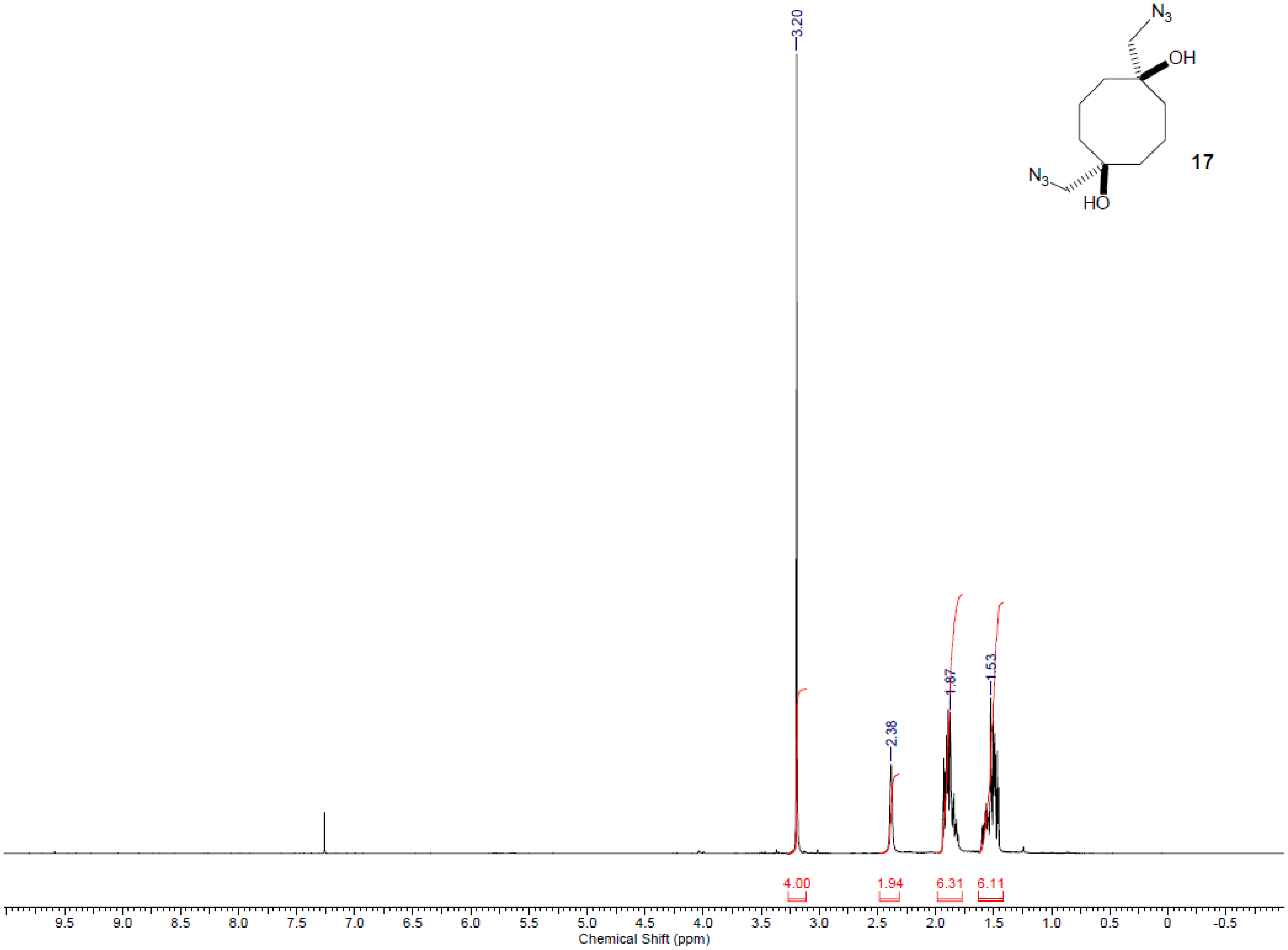

17

^{13}C NMR ($CDCl_3$) spectrum of compound **17**

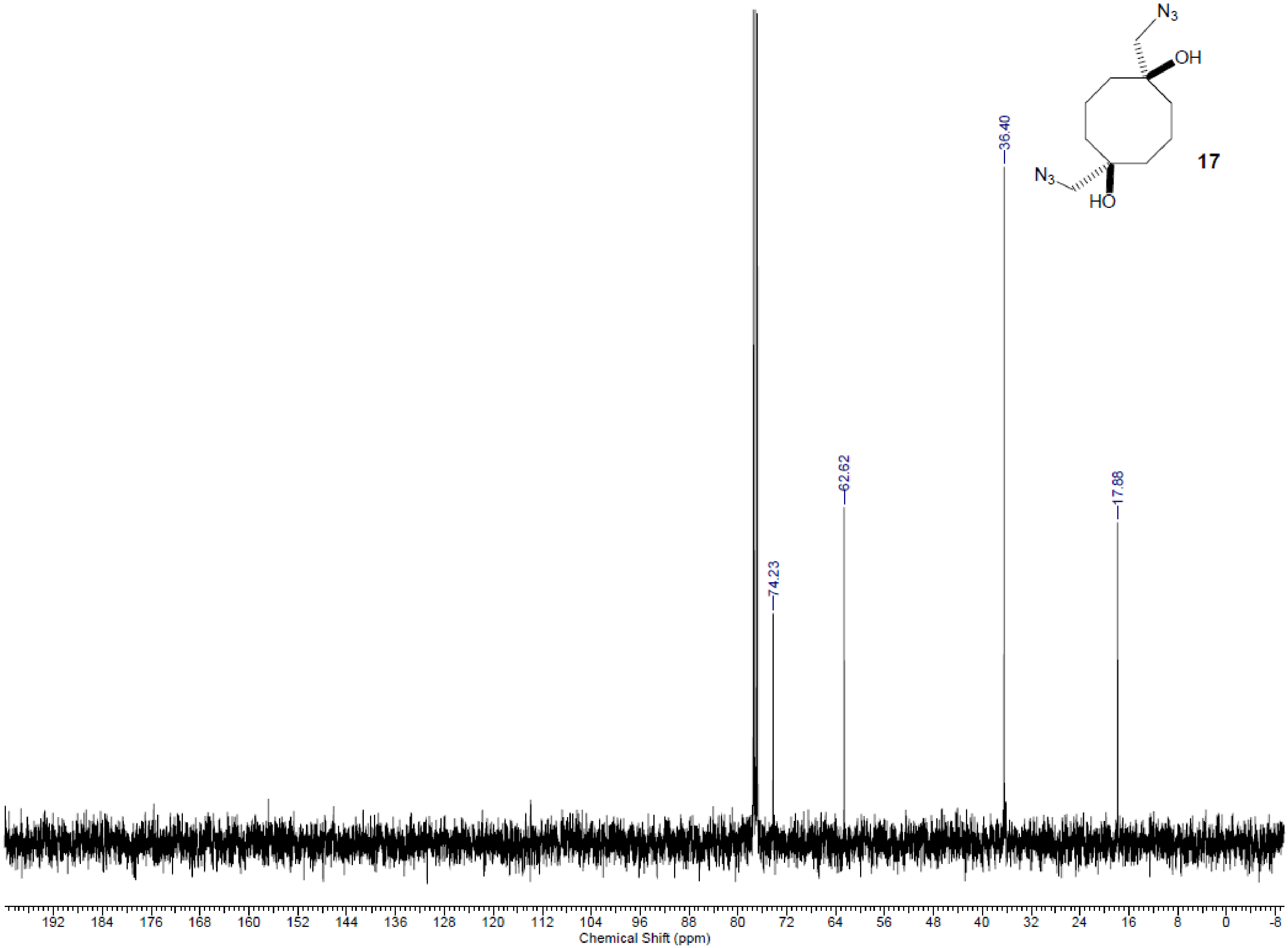

^{1}H NMR ($CDCl_3$) spectrum of compound **18**

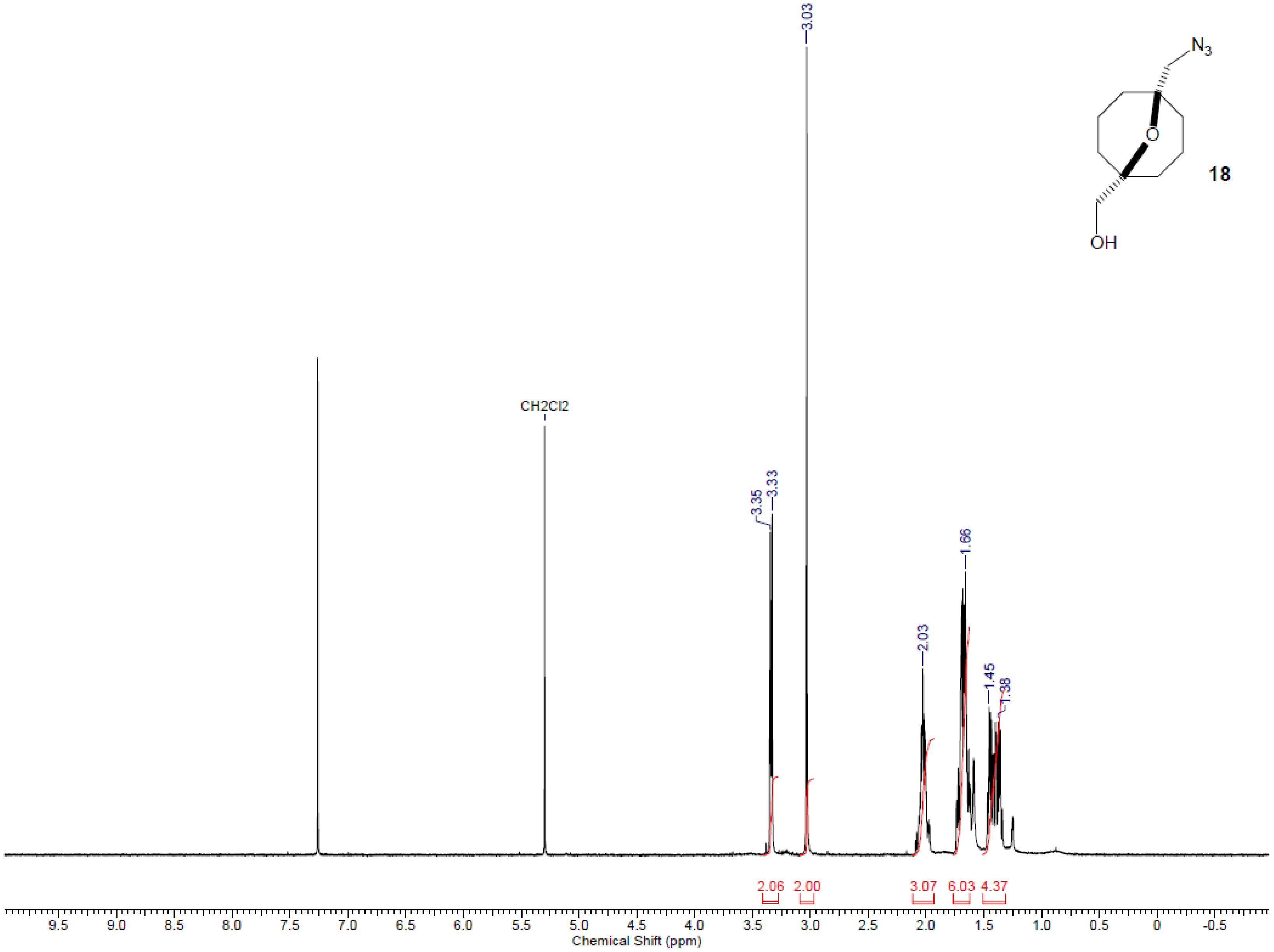

^{13}C NMR ($CDCl_3$) spectrum of compound **18**

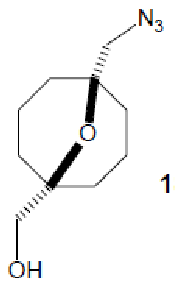

18

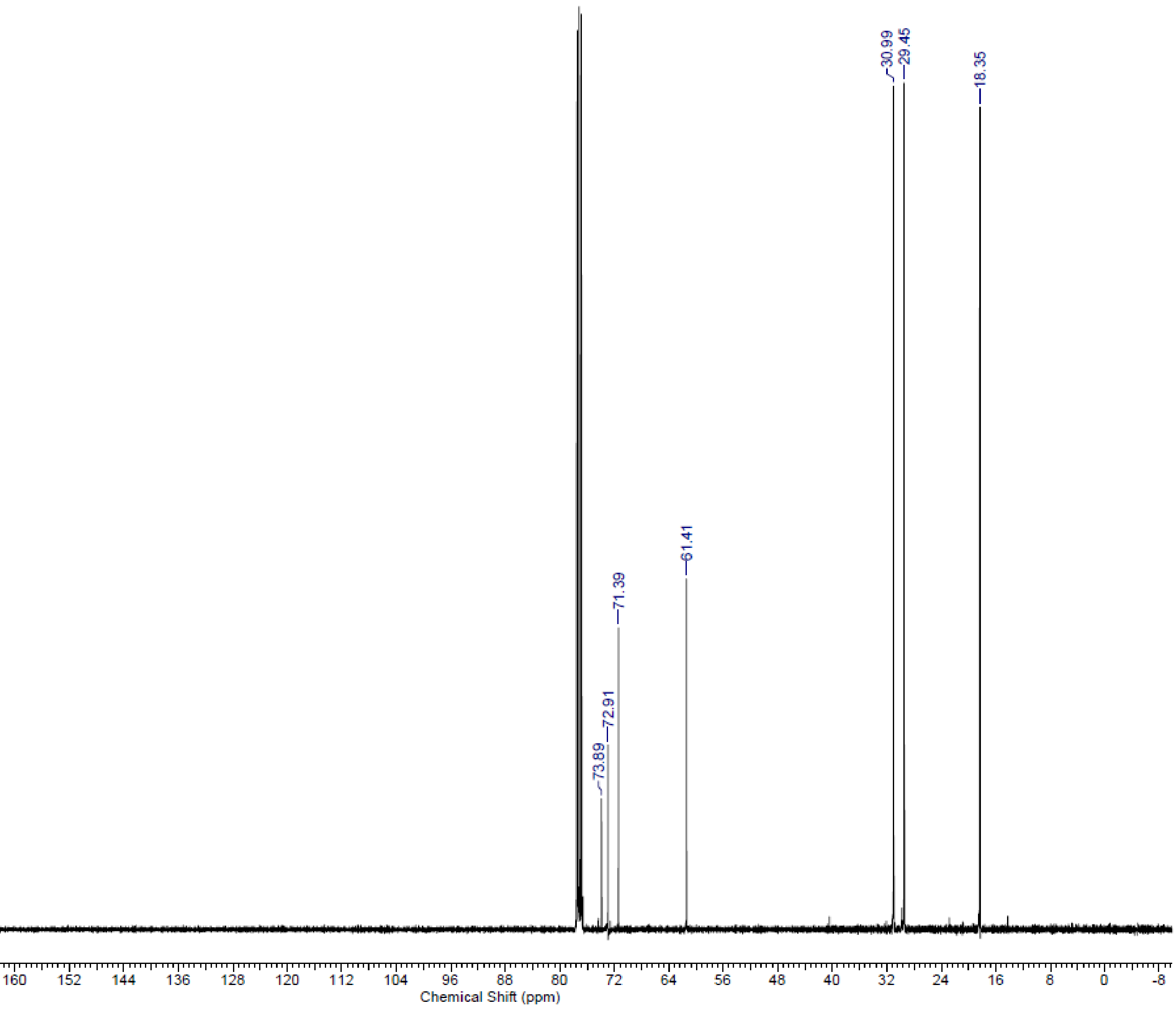

HSQC NMR ($CDCl_3$) spectrum of compound **18**

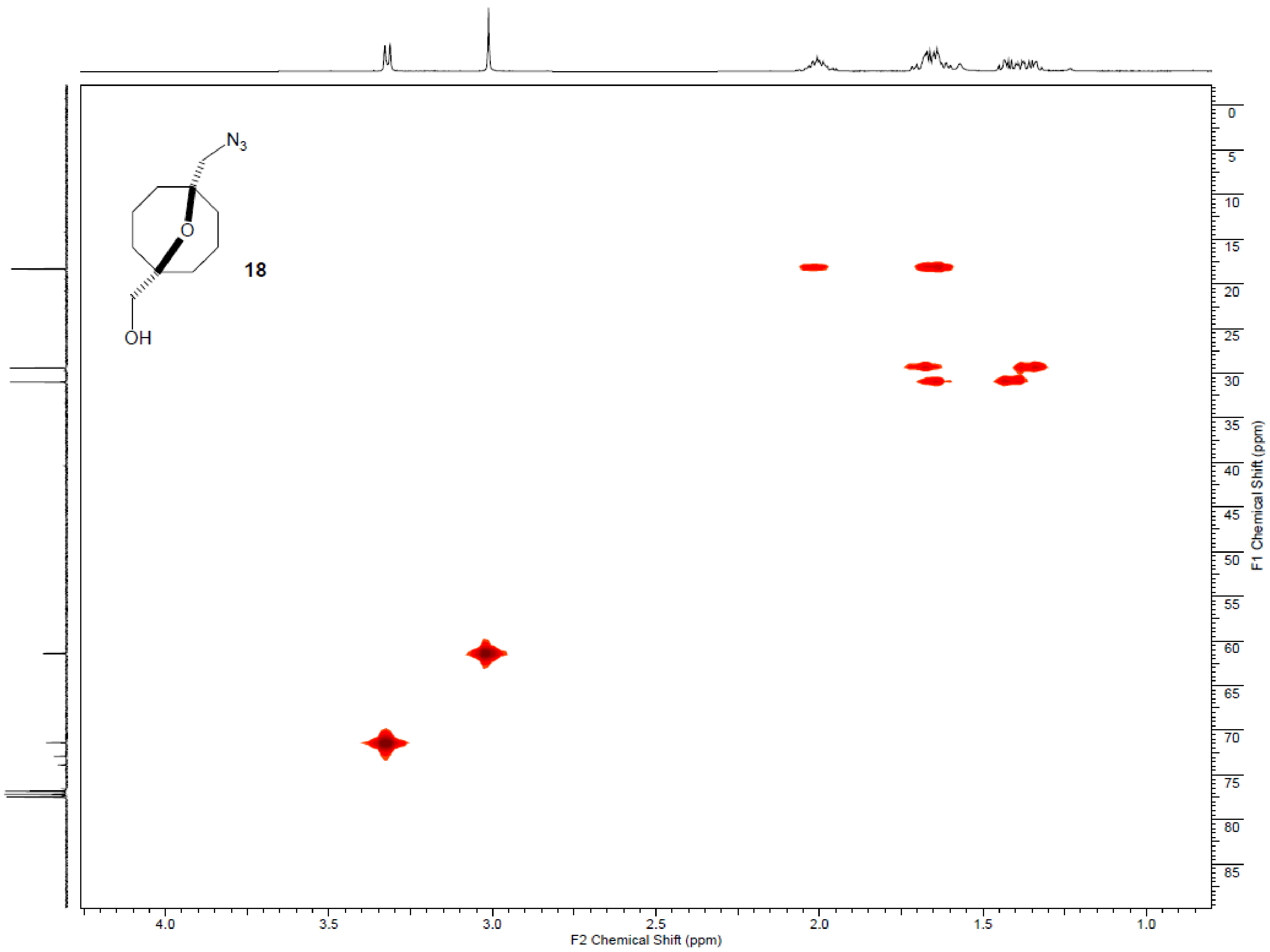

HMBC NMR ($CDCl_3$) spectrum of compound **18**

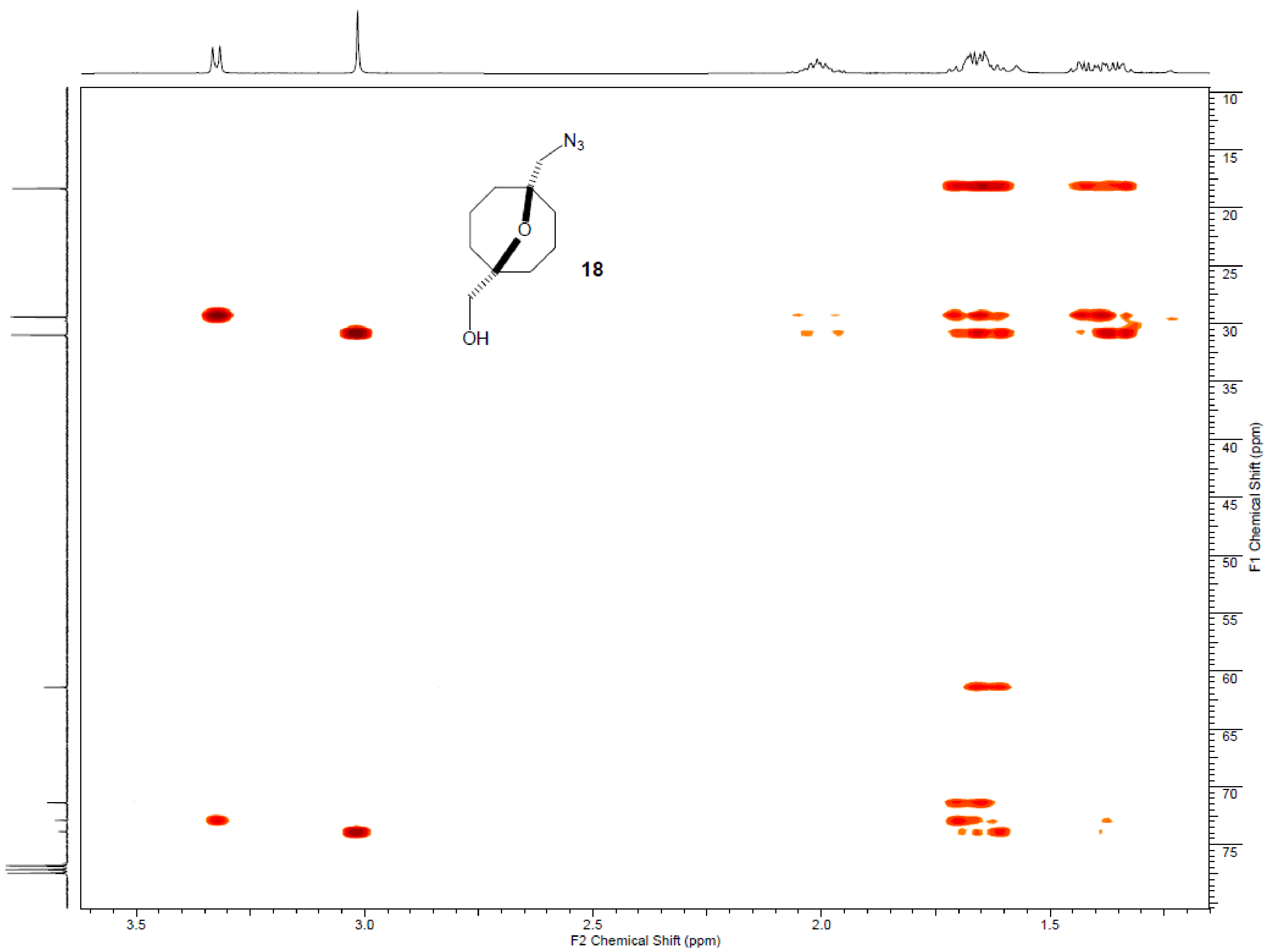

4. DFT optimized structures

6a-1

cartesian

set=L1

6	1.79963260	0.15878287	-1.08597469
6	1.89093158	-1.25880544	-0.49018592
6	1.50555596	-1.45234631	0.98802497
6	0.01683008	-1.61566962	1.33844543
6	-0.83943121	-0.34070610	1.45503467
6	-1.40698903	0.20617975	0.14870925
6	-0.57105541	1.18305640	-0.65585175
6	0.43886476	0.62048797	-1.64046427
1	2.50731202	0.20091718	-1.93245090
1	2.17214286	0.89454685	-0.34807031
1	-0.03437108	-2.12204180	2.31816051
1	-0.47063328	-2.29496099	0.61620487
1	2.94349335	-1.57887240	-0.58875160
1	1.30427004	-1.95722427	-1.11664628
1	1.93810965	-0.62884520	1.58938181
1	2.01429230	-2.37044751	1.33266223
6	-2.87260224	0.26810271	-0.03247538
8	-2.10413356	-0.77295767	-0.66137079
1	-3.28498447	1.02800083	-0.70437603
1	-3.51822430	-0.05384196	0.79619450
6	-0.39129501	2.54625509	-0.11919515
8	-1.31479808	2.31600432	-1.19025760
1	0.51552293	3.10976587	-0.37966782
1	-0.83915828	2.80743946	0.85004135
1	-0.04657081	-0.20688666	-2.18825582
1	0.60976535	1.42878182	-2.37109306
1	-0.26587291	0.45616832	1.96201585
1	-1.69660383	-0.57088350	2.11021194

$end

Energy = -540.52504326

6a-2

cartesian

set=L1

6	1.72334779	-0.26165436	-1.11651828
6	1.34105429	-1.57593357	-0.39567061
6	1.33304690	-1.49448697	1.14240204
6	0.56443461	-0.31808711	1.76122226
6	-0.93957908	-0.24842665	1.45568188
6	-1.33828710	0.08994151	0.02243619
6	-0.48970804	1.09316714	-0.74420784
6	0.54432059	0.53397336	-1.70877541
1	2.39174556	-0.49794599	-1.96212312
1	2.31467576	0.38458767	-0.44129566
1	1.03571642	0.63387107	1.45764086
1	0.68234612	-0.37079843	2.85816679
1	2.05909105	-2.36492752	-0.67800274
1	0.35741970	-1.91631407	-0.75893116
1	2.37827463	-1.43119347	1.49824413
1	0.92808187	-2.44211512	1.54360447
6	-2.75826056	-0.03906826	-0.35869122
8	-1.80105937	-1.01996550	-0.79276272
6	-0.33786551	2.45630259	-0.19648571
8	-1.22343035	2.22094807	-1.29730211
1	0.57493998	3.02413067	-0.42513881
1	-0.82014705	2.71658140	0.75593352
1	-1.38776069	0.51931770	2.11333993
1	-1.42766430	-1.20697206	1.70797307
1	-0.00255799	-0.11752729	-2.41337933
1	0.93038933	1.38965964	-2.28820358
1	-3.15299040	0.62571921	-1.13502405
1	-3.47957416	-0.38278368	0.39586722

$end

Energy = -540.52718472

6a-3

cartesian

set=L1

6	1.66079907	-0.12806939	-1.36748532
6	1.39294258	-1.40568013	-0.55756574
6	1.66452634	-1.35106940	0.95327735
6	0.79675670	-0.39261515	1.79439088
6	-0.71941943	-0.41559614	1.48550674
6	-1.20512632	0.71134253	0.58321969
6	-0.72573790	0.77131462	-0.85566349
6	0.75355898	1.08545060	-1.06824215
1	1.55701345	-0.38606354	-2.43658069
1	2.71327691	0.18156876	-1.23233101
1	1.15750409	0.64532187	1.70929080
1	0.94053055	-0.66375445	2.85401944
1	2.01978765	-2.21069494	-0.98137615
1	0.34880029	-1.72453205	-0.72986606
1	2.72863683	-1.10355921	1.12406776
1	1.52378985	-2.37486834	1.34687783
8	-1.19747845	2.01058302	1.23002073
6	-2.46462270	1.40352855	0.91668695
1	-3.04868748	1.04942770	1.77725862
1	-3.02514660	1.88221129	0.10675970
8	-1.62551529	1.47257670	-1.75332664
6	-1.50038812	0.05214657	-1.88893580
1	-1.27805661	-0.31493965	2.43010385
1	-1.01201466	-1.39005945	1.05265305
1	0.80188804	1.78669327	-1.91762864
1	1.11391000	1.64334367	-0.18994552
1	-2.37869845	-0.53619155	-1.58746337
1	-0.99282932	-0.29781576	-2.79772280

$end

Energy = -540.5245888600

6b-1

cartesian

set=L1

6	1.80409115	0.03277034	-1.25762373
6	1.76322997	-1.24220672	-0.39292783
6	1.55966952	-1.04693816	1.13367062
6	0.16745816	-1.38810503	1.69918513
6	-0.91820241	-0.30423443	1.58392410
6	-1.29073128	0.06356109	0.15895424
6	-0.41558880	1.11156849	-0.54010237
6	0.45242387	0.62830859	-1.68788183
1	2.36237523	-0.19224347	-2.18370141
1	2.37994496	0.81239532	-0.72739024
1	0.27517480	-1.61807471	2.77410765
1	-0.20350910	-2.31099430	1.21833523
1	2.72893930	-1.75585719	-0.53822171
1	0.99299449	-1.93152354	-0.78518845
1	1.83050488	-0.01262403	1.41518512
1	2.28223924	-1.69914060	1.65338043
6	-2.68568050	-0.08735658	-0.30991341
8	-1.68515028	-1.04790951	-0.68274601
1	-3.05197383	0.55456927	-1.12297233
1	-3.45371030	-0.44431546	0.39000479
8	0.21600485	2.06054352	0.35430012
6	-0.85682453	2.52234358	-0.48147813
1	-0.12815527	-0.12318305	-2.24937605
1	0.64408603	1.47750806	-2.36573774
1	-0.58403247	0.61837879	2.08804447
1	-1.82397016	-0.65992716	2.10390140
1	-1.79024399	2.77239819	0.04146990
1	-0.57136352	3.21028869	-1.28920198

$end

Energy = -540.52480541

6b-2

cartesian

set=L1

6	1.79567815	-0.24308076	-1.02401611
6	1.45542962	-1.54148291	-0.25464791
6	1.42074360	-1.40708177	1.28124406
6	0.64309259	-0.21390742	1.85605810
6	-0.86325799	-0.15498451	1.55506193
6	-1.26515595	0.05999505	0.10160318
6	-0.44285103	1.05709652	-0.71077276
6	0.59583289	0.48536749	-1.66231559
1	2.48733466	-0.48486824	-1.84928700
1	2.34246764	0.46025438	-0.37033545
1	1.10280394	0.73135627	1.52398548
1	0.75800395	-0.23142701	2.95466145
1	2.20913425	-2.31027201	-0.49762108
1	0.49286677	-1.93678593	-0.61725117
1	2.46042587	-1.32501075	1.64931038
1	1.01856799	-2.34601776	1.70550710
6	-2.67156977	-0.19245851	-0.28122644
8	-1.64244408	-1.12387450	-0.64765485
8	-0.03568575	2.23164905	0.03487002
6	-1.02182798	2.38902601	-0.99608458
1	-1.29168786	0.67973636	2.13826186
1	-1.36295172	-1.08055155	1.89316505
1	0.06868462	-0.21133400	-2.33793509
1	0.97443901	1.31923175	-2.27809455
1	-3.11726509	0.37260767	-1.11025017
1	-3.37945190	-0.54167964	0.48356125
1	-2.03651350	2.62320270	-0.64835008
1	-0.69484292	2.93529403	-1.89144705

$end

Energy = -540.52609296

6b-3

cartesian

set=L1

6	1.72471573	-0.12326188	-0.99802466
6	1.34871280	-1.45201485	-0.30311868
6	1.28662451	-1.37454889	1.23304628
6	0.46903058	-0.21238902	1.81151095
6	-1.02637827	-0.19728135	1.46419109
6	-1.41340647	0.11566340	0.02101602
6	-0.57332660	1.14947739	-0.73152228
6	0.55072558	0.64703439	-1.62982269
1	2.43455012	-0.33194705	-1.81655992
1	2.26875047	0.53012967	-0.29164850
1	0.91954454	0.74849268	1.50557827
1	0.55571506	-0.24388246	2.91187117
1	2.08785517	-2.22682349	-0.56968491
1	0.38293031	-1.80916571	-0.70129338
1	2.31863877	-1.28724246	1.62074814
1	0.89070295	-2.33010248	1.62424841
8	-2.84149602	0.25590488	-0.12989814
6	-2.20085887	-0.85019875	-0.77420235
1	-2.40886133	-1.84388001	-0.35209498
1	-2.17173563	-0.80412740	-1.87170967
6	-0.53773577	2.53482041	-0.21751632
8	-1.33849775	2.20261270	-1.35726547
1	0.34745099	3.15656959	-0.41284819
1	-1.09584616	2.78614733	0.69464850
1	-1.51261506	0.58167978	2.07986343
1	-1.49401781	-1.15547894	1.75296142
1	0.08730801	0.00251962	-2.40021338
1	0.94152013	1.53129292	-2.16226017

$end

Energy = -540.5218889
